# AhR as a Common Denominator in Immunity and Inflammation in Chronic Lung Diseases: Molecular and Clinical Insights

**DOI:** 10.3390/diseases14070224

**Published:** 2026-06-23

**Authors:** Maria L. Perepechaeva, Alevtina Y. Grishanova, Valentin A. Vavilin

**Affiliations:** Institute of Molecular Biology and Biophysics, Federal Research Center of Fundamental and Translational Medicine, Timakova Str. 2, Novosibirsk 630060, Russia; aiugrishanova@frcftm.ru (A.Y.G.); valentin.vavilin@frcftm.ru (V.A.V.)

**Keywords:** AhR signaling, AhR ligands/agonists, aryl hydrocarbon receptor, respiratory system, chronic lung diseases, inflammatory lung diseases, immune response, inflammation

## Abstract

The respiratory system is directly exposed to various environmental factors, and specifically allergens and environmental pollutants, which are ligands/agonists of the aryl hydrocarbon receptor (AhR) and promote chronic lung diseases in humans. AhR, a ligand-activated transcription factor, is involved in the metabolism of xenobiotics, assigning their carcinogenic and toxic effects, and is also involved in normal homeostasis, organogenesis, and immune system function. Exogenous and endogenous AhR ligands are both high-molecular-weight compounds with a planar structure and low-molecular-weight compounds of diverse chemical structures. After entering the cell, the ligands bind to AhR and induce the activation of signaling cascades. The lung immune system responds to pathogens and environmental toxins first with a pro-inflammatory innate immune response, and then with an anti-inflammatory adaptive immune response. An imbalance between these immune systems may have an effect on the course of the disease. Activation of AhR by exogenous or endogenous ligands can affect this balance and lead to dysregulation of the immune response, leading to inflammatory complications in the lungs. Individual features of AhR expression or components of the AhR-dependent signaling pathway may also play a role in the superposition of the functions of these two links of immunity. This review summarizes advances in the comprehension of AhR’s role in immunomodulation and inflammatory responses in the lungs following data in experimental rodent models, in vitro studies utilizing lung structural cells and isolated immune cell lines, and humans. The molecular mechanisms of AhR’s regulation of immunity and inflammation and the potential of AhR as a therapeutic target for inflammatory lung disease are also considered.

## 1. Introduction

The lungs, as the primary organs of the respiratory system, are exposed to a large number of diverse infectious pathogens and different groups of toxins, allergens, and other air pollutants. Lung physiology is balanced by the coordinated action of different cells, including the respiratory epithelium, alveolar epithelial cells, and interstitial cells. Under healthy conditions, a balance between the functions of these cells provides the maintenance of lung function. Conflicts in lung physiology, arising from exposure to pathogens, toxins, allergens, air pollutants, etc., are accompanied by immunological and inflammatory processes. In many cases, inflammation is the starting point in a chain of events leading to dysfunction in lung physiology.

The impact of damaging factors on the lungs entails an initiation of pro-inflammatory response involving components of the innate immune system, in particular, the mucociliary clearance mechanism, humoral factors, and cellular elements (macrophages, dendritic cells, monocytes, neutrophils, etc.). Then an anti-inflammatory reaction of the adaptive links of the immune system gets activated with the involvement of T and B lymphocytes. An imbalance between pro- and anti-inflammatory immunity contributes to the onset and progression of disease and affects its outcome [1,2,3].

One of the factors disrupting the balance between the innate and adaptive components of the immune system is related to the activation of the aryl hydrocarbon receptor (AhR). AhR functions as a transcription factor affecting the regulation of gene signaling networks involved in a variety of physiological and pathological processes. This is associated with the role of AhR in the metabolism of environmental pollutants [4]. Furthermore, AhR plays an immunoregulatory role in inflammatory processes, controlling the expression of a number of cytokines in immune cells and participating in the regulation of the innate and adaptive immune response [5,6,7,8].

Chronic respiratory diseases are one of the five areas of non-communicable inflammatory diseases which contribute most to global mortality and morbidity and include chronic obstructive pulmonary disease (COPD), asthma, bronchiectasis, interstitial lung diseases, occupational lung diseases, and pulmonary hypertension [9]. COPD, which includes conditions such as chronic bronchitis and emphysema, is a serious global health threat, as it currently ranks fourth among the leading causes of death [10,11,12]. According to the World Health Organization, it will become the third leading cause of death globally by the end of 2030 [13,14]. Asthma is characterized by airway inflammation, bronchial hyperreactivity, and structural airway remodeling. Asthma manifests as an abnormal bronchospastic response to allergens and irritants, in which several cellular components of the innate and adaptive immune systems, along with epithelial cells, cause bronchial hyperreactivity [15]. Pulmonary fibrosis is a characteristic feature of a number of lung diseases, including cystic fibrosis, idiopathic pulmonary fibrosis, and chronic allergic diseases. Fibrosis is manifested by excessive accumulation of extracellular matrix components in tissues as a result of chronic inflammation and impaired tissue regeneration. The pathogenesis of fibrosis depends on complex interactions between different immune cell subpopulations, mesenchymal cells, and parenchymal cells [16].

In this review, we analyze the data on the immunobiology of the AhR signaling pathway related to chronic inflammatory lung diseases based on data obtained from experiments and clinical studies.

## 2. The Immunological System in Inflammatory Processes in the Lungs

Inflammation is a critical physiological and pathophysiological process characterized by reactions with the participation of immune cells, blood vessels, and molecular and cellular mediators to protect the body from injury and pathogen invasion [3,17,18]. Acute and chronic inflammation are mediated by different mechanisms [19].

An acute inflammatory response is initiated by exposure to an inflammatory agent and is aimed at eliminating the cause of the damage, removing damaged cells, and initiating the recovery process [20]. Chronic inflammation can be a consequence of acute inflammation, but in some cases, it is an independent response [20]. Chronic inflammation can be caused by prolonged exposure to low-intensity irritants and constant stimulation by inflammatory factors. Furthermore, chronic inflammation can be caused by factors such as chronic infections, toxins, and foreign bodies, as well as by the development of autoimmune and allergic processes; the presence of genetic defects in cells mediating inflammation; and the action of chemical inducers, which cause oxidative stress and mitochondrial dysfunction with increased production of free radicals, oxidized lipoproteins, etc. [20,21].

The inflammatory response is regulated by a complex network of innate and adaptive immunity, which induces an immune response depending on the pathogen’s nature and ensures immune homeostasis of the lungs [22]. The activity of the immune response is modulated by key immune effector cells of innate immunity, such as macrophages, certain subgroups of dendritic cells (DCs), myeloid-derived suppressor cells (MDSCs), natural killer (NK) cells, and innate lymphoid cells (ILCs), as well as by cells of adaptive immunity, including T-regulatory cells (Tregs) and B lymphocytes [23,24,25,26,27,28]. The immune mechanisms of lung defense are regulated by small signaling glycoprotein molecules named cytokines [23,24,27,28], which enable each immune cell type to perform a specific role in the immune system and interact with other immune cells. Pro-inflammatory cytokines (TNF-α, IL-1, IL-6, IL-12, IFN-γ) stimulate inflammation, activate immune cells, and enhance the immune response. Anti-inflammatory cytokines (IL-1, IL-4, IL-10, IL-11, IL-13, TGF-β) regulate the pro-inflammatory cytokine response. Cytokines act in association with specific cytokine inhibitors and soluble cytokine receptors.

Innate immune molecules detect pathogens using pattern recognition receptors (PRRs) expressed in macrophages, neutrophils, and DCs [29]. PRRs detect various microbial components, known as pathogen-associated molecular patterns (PAMPs), and molecules secreted by necrotic cells and damaged tissues, known as damage-associated molecular patterns (DAMPs) [30].

Macrophages are a large family of innate immune cells involved in the detection, phagocytosis, and destruction of bacteria and other harmful organisms. They are capable of detecting the waste products of bacteria and other microorganisms using a system of recognition receptors, such as Toll-like receptors (TLRs). These receptors specifically bind to various components of pathogens, such as sugars (LPS), RNA, DNA, and extracellular proteins. Macrophages play a key role in a wide range of physiological functions, including development, tissue repair, immunity, and maintaining a stable internal environment [23].

Under different pathophysiological conditions, activated macrophages can differentiate into phenotypically distinct states, including classically activated (M1) and alternatively activated (M2) macrophages [31]. The division into these two subclasses of macrophages is based on the production of nitric oxide and arginine [32,33]. M1 macrophages are characterized by the production of pro-inflammatory cytokines (IL-1, IL-6, IL-12, TNF-α, IFN-γ) and chemokines and participate in various inflammatory processes. They also promote the differentiation of inflammatory T cells, including Th1 and Th17 cells, which, in turn, mediate inflammation [31]. In contrast, M2 macrophages produce anti-inflammatory cytokines (IL-10 and IL-13) and play an important role in the resolution of inflammation and mediation of wound healing [34].

Alveolar and interstitial macrophages in the lungs respond to a wide range of stimuli [35]. Alveolar macrophages are involved in phagocytosis and the production of cytokines, in particular TNF-α and IFN-γ. Interstitial macrophages are more prone to release cytokines associated with the adaptive immune response [36,37].

DCs are professional antigen-presenting cells, which link innate and adaptive responses, inducing tolerance or immunity [24,38]. They capture endogenous and exogenous antigens and transport them into secondary lymphoid organs to activate T cells [39]; in this way, they initiate adaptive immune responses and function as “sentries” of the immune system. Depending on the cytokines and maturation stages, DCs can affect the T cell response by inducing their differentiation into T helper 1/2 (Th1/Th2) cells and Tregs, or by stimulating an energetic response. For instance, tolerogenic DCs, which have the capacity of producing anti-inflammatory cytokines and reducing the expression of costimulatory molecules and the expression of inhibitory molecules, can increase the number of Treg cells [40]. Tregs produce IL-10 and TGF-β to suppress the immune response. Meanwhile, Th1 cells are responsible for cellular immunity, and Th2 cells are essential for humoral and antiparasitic immunity, as well as allergic reactions.

MDSCs are a population of myeloid cells that represent a pathological state of activation of monocytes and relatively immature neutrophils [41].

NK cells are cytotoxic lymphoid cells of the innate immune system, which defend the body against pathogens and certain cancers. NK cells play a key role in the regulation of immunity [27,42] by interacting with macrophages, T lymphocytes, DCs, and endothelial cells. In addition, NK cells can exhibit adaptive properties, such as antigen-induced clonal expansion and the induction of long-term memory, which attests to their regulatory role in both innate and adaptive immunity [27,42].

ILCs are analogs of CD4^+^ T lymphocytes (CD4^+^ T helpers) that regulate immune responses and maintain immune homeostasis in mucosal surfaces, including the lungs. ILCs lose rearranged antigen receptors and respond to pathogens primarily without cell-contact-based signals from antigen-presenting cells [43,44]. ILCs are divided into three main groups based on their transcriptional patterns and effector functions: ILC1 cells, which resemble Th1 cells; ILC2 cells, which are characterized by the expression of the transcription factor GATA3, a master regulator of Th2 cells; and ILC3 cells, which mimic Th17 cells and express the transcription factor RORγt [43,45,46,47,48,49,50,51].

The epithelium is also involved in the immune architecture of the lungs, serving as a physical barrier between the lumen and the underlying submucosa and protecting lung tissue from pathogens. In addition to functioning as a physical barrier, alveolar epithelial cells play a key role in the immune response via producing cytokines and chemokines responsible for the activation and differentiation of immune cells and acting as antigen-presenting cells for specific T cells [52,53].

Mast cells, neutrophils, basophils, and eosinophils also function as part of the innate immune system.

CD4^+^ T helper cells (Th cells) are mediators of adaptive cellular immunity since they are involved in the activation of other immune cells, such as antibody-producing B cells and cytotoxic T cells, as well as in the polarization of macrophages toward an inflammatory phenotype [41].

CD4^+^ T helper cells are divided into subgroups based on the characteristics of the cytokines they secrete and their effector function: Th1, Th2, Th17, and Treg. Each subgroup is activated by a specific set of cytokines and transcription factors [54].

Uncommitted CD4^+^ T cells can differentiate into Th1 or Th2 cells depending on the prevailing pro-inflammatory or anti-inflammatory environment. Th1 and Th2 cells have different cytokine production patterns and functions: Th1 cells participate in cellular immunity, while Th2 cells participate in humoral immunity [54].

Th17 is a subgroup of effector T cells that express two orphan nuclear receptors, namely Retinoic Acid-Related Orphan Receptors (RORs) RORα and RORγ, which are essential for Th17 cell differentiation [54]. Th17 cells are a critical component of the immune system, performing both protective and pathogenic roles. They participate in the body’s defense against certain pathogens and contribute to the development of autoimmune diseases. Th17 cells include various subtypes associated with specific functions and molecular phenotypes depending on the cytokine environment that determines pathogenic or nonpathogenic states [55,56].

Regulatory T cells (Treg) are CD4^+^FoxP3^+^ T cells, which participate in regulating or suppressing the activity of other immune cells. Tregs control the immune response to self and foreign antigens and help prevent autoimmune diseases. Tregs express the transcription factor Forkhead Box 3 (FoxP3), which plays a key role in maintaining immunological homeostasis and preventing the impairment of peripheral self-tolerance [57,58].

CD8^+^ T cells are cytotoxic cells involved in immune defense against intracellular pathogens, including viruses and bacteria, and are the end effectors of cancer immunity.

B cells are at the center of the adaptive humoral immune system and are responsible for mediating the production of antigen-specific immunoglobulins (Ig) [28]. B cells undergo a genetic process that makes it possible to modify the constant regions of the antibody molecule. These modifications facilitate the production of antibodies of different classes, switching from IgM to IgG, IgA, or IgE while maintaining antigen specificity [59].

The initial pro-inflammatory immune response is provided by epithelial cells, DCs, and alveolar macrophages, which coordinate the production of lysozyme, lactoferrin, antileukoproteinases, pro-inflammatory cytokines and chemokines, secretory IgA, defensins, and cathelicidins [60,61,62]. Then mediators of the innate immune system trigger and activate a complex of adaptive immune responses with participation of Th1, Th2, and Th17, and their regulation involves the activation of transcription factors via various signal transduction pathways [60].

The inflammatory response of innate immune cells is triggered by PAMPs. Monocytes/macrophages coordinate immune responses. Activated macrophages produce pro-inflammatory mediators, such as nitric oxide (NO), prostaglandin E_2_ (PGE_2_), and cytokines, along with mast cells, which secrete cytokines, chemokines, histamine, and prostaglandins [21,63]. These events promote lymphocyte infiltration, exacerbating inflammation [21].

The inflammatory process is synchronized with the activation of signaling pathways mediated by the NLRP3 inflammasome, nuclear factor κB (NF-κB), JAK/STAT, MAPK, and PI3K-AKT [21], as well as Kruppel-like factor 6 (KLF6) [64]. Resolution of acute inflammation involves programmed cell death and elimination of activated inflammatory cells [65]. The disruption of this process leads to chronic inflammation and the development of various non-infectious lung diseases [17,18].

## 3. AhR

AhR is a ligand-activated transcription factor and a member of the basic, helix–loop–helix motif-containing Per-ARNT-Sim (bHLH/PAS) family. The bHLH/PAS proteins sense a variety of extra- and intracellular signals and transform them into a cellular response, forming molecular signal transduction pathways [66,67,68].

The structure of human AhR includes the bHLH domain, PAS A and PAS B domains, and transactivation domains (TADs), the activity of which is mediated by a number of coactivators [67,69,70,71]. The bHLH domain contains signals of nuclear localization and nuclear export, and participates in AhR binding to DNA and in protein dimerization processes [72,73,74]. PAS domains are involved in ligand binding and protein–protein interactions [67,75,76,77,78].

AhR is regulated through a multi-layered process, beginning with epigenetic and basal transcription, moving through ligand-activated transcription complexes, and concluding with strict post-translational control via the 26S proteasome [79,80,81]. Basal AhR transcription is driven by promoter regions rich in GC-boxes that bind the Sp1/Sp3 family of transcription factors [82]. The AhR promoter is heavily influenced by epigenetic modifications. Hypermethylation can silence AhR expression and histone acetylation acts as an on/off switch for constitutive AhR expression [81]. Transcription factors like nuclear factor erythroid 2-related factor 2 (NRF2) directly bind to Antioxidant Response Elements (AREs) in the AhR promoter to upregulate its transcription. The transcription of the AhR promoter is tightly controlled by a newly discovered molecular axis termed the AhR-NRF2-JDP2 gene battery. Jun Dimerization Protein 2 (JDP2) acts alongside nuclear factor erythroid 2-related factor 2 (NRF2) to direct chromatin control at the nucleosome level. This complex determines the baseline rate of AHRAhR transcription during periods of high oxidative stress [83].

AhR is activated by a wide range of exogenous and endogenous ligands. Exogenous (anthropogenic) ligands are also called “classical” because they were the first identified as AhR ligands. These are planar, hydrophobic molecules with a polycyclic structure similar to polycyclic aromatic hydrocarbons, halogenated aromatic hydrocarbons, polyphenols, and dioxins [76,84,85]. AhR-dependent genes metabolize these compounds to highly reactive toxic metabolites [76,86,87,88]. Later, a variety of small-molecular-weight compounds were identified as AhR ligands/agonists [89,90]. These are endogenous AhR ligands, which are physiological molecules including tryptophan metabolites [84,91,92,93,94], tetrapyrroles [76], arachidonic acid (LXA4) metabolites [76,95], and heme metabolites [96]. Depending on the compound binding to its allosteric site, ligands are classified as agonists or antagonists [97]. Because AhR signaling is diverse, many ligands do not fit strictly into the binary agonist/antagonist category. Instead, they act as selective AhR modulators, exhibiting tissue-specific or gene-specific combinations of agonist and antagonist activity [98].

**Figure 1:**
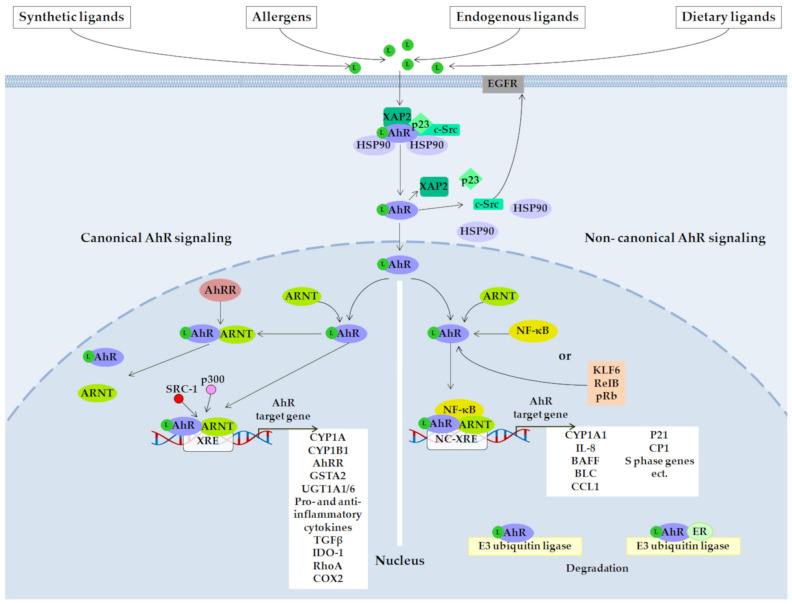
AhR signaling pathways. AhR ligands, including synthetic substances, food compounds, allergens, and endogenous ligands, enter the cell and bind to AhR. Intact AhR exists in the cytoplasm as a complex with proteins (hepatitis B virus X-associated protein 2 (XAP-2), heat shock protein 90 (HSP90), co-chaperone p23, and additional proteins, including c-Src kinase). Ligand binding alters the conformation of AhR, the complex dissociates, and AhR translocates to the cell nucleus, forming a dimer with its classical partner protein, ARNT. In this case, it forms a dimer either with the AhR nuclear transporter or with other proteins (transcription factors of the nuclear factor kappa B (NF-κB) family, the transcription factor Krüppel-like factor 6 (KLF6), the retinoblastoma protein (pRb), or nuclear receptors (for example, the estrogen receptor α). In the first case, the so-called canonical pathway is realized, and in the second, the non-canonical pathway. The AhR dimer with a partner protein binds to xenobiotic-responsive elements (XREs) in DNA, which induces transcription of AhR target genes. Adapted from Ref. [119], licensed under CC BY 4.0.

The trigger of the AhR activation is the binding of an inactive receptor to a ligand molecule (Figure 1). In the absence of a ligand, AhR is localized in the cytoplasm and bound by a chaperone complex, which consists of two HSP90 molecules, single molecules of the co-chaperone p23 and X-associated protein-2 (XAP2) [70,99,100], and Src tyrosine kinase [101]. The AhR chaperone complex stabilizes AhR in the cytoplasm and maintains it in a conformation that has high affinity for its ligands [102]. Ligand binding to cytosolic AhR triggers a conformational change that releases AhR from the co-chaperone complex and exposes the region of nuclear localization signal, enabling the AhR–ligand complex to be imported into the nucleus. After ligand binding to AhR, XAP2 dissociates from the complex, exposing the N-terminal nuclear localization signal of AHR and the adjacent nuclear export signal. The AhR–ligand complex then moves into the nucleus in a transportin- and importin-β-dependent manner [103]. It is not entirely clear which AhR chaperone complex members translocate to the nucleus together with AhR. This co-translocation is considered ligand-specific and cell-type-specific [75,104]. In the nucleus AhR dimerizes with its partner protein, ARNT [70,76,105]. The AhR-ARNT heterodimer binds to the consensus sequence of the xenobiotic-responsive element (XRE), which functions as an enhancer; the TA domains of AhR, via transcriptional coactivators, transmit a signal from the enhancer to the promoter, initiating transcription of target genes [76,77,106,107,108,109].

The specific structure of the bound ligand alters the DNA topology and the repertoire of responsive target genes, indicating that AhR binding is not a simple on/off state [110,111,112]. AhR defies classical transcription factor logic in several key ways [113]. Unlike classical methylation readers (which target modified DNA), AhR functions by selectively binding to the unmethylated form of its target XREs [113]. AhR binding does not just read DNA; it remodels the epigenome. It actively recruits coactivators and alters local histone modifications, rendering previously silent chromatin accessible for transcription [114,115]. Rather than a simple on/off switch, AhR activation leads to graded, reciprocal epigenetic regulation, such as simultaneously repressing inflammatory genes while inducing regulatory or adaptive genes [116,117]. Activated genes include CYP1A1, CYP1A2, CYP1B1, members of subfamily 1 of the cytochrome P450 family, and a number of other xenobiotic metabolism genes, the activation of which causes a wide range of physiological and toxic effects [76,106,107,108,109,118].

The termination of AhR signaling is tightly controlled by several interconnected feedback mechanisms [81,120]. After AhR-ARNT binds to DNA, the receptor undergoes a conformational change, and is then exported from the nucleus to the cytoplasm, where E3 ubiquitin ligases label it with multiple ubiquitin molecules. Ubiquitinated AhR is recognized and degraded by the 26S proteasome [120]. The aryl hydrocarbon receptor repressor (AHRR) regulates signaling by competitively inhibiting AhR. This action prevents AhR from binding to the ARNT protein, halting the transcription of AhR-regulated genes. AHRR can bind directly to XREs, actively blocking AhR access to these regions. AHRR recruits corepressor complexes to DNA, locking target genes in an inactive state [121]. PARP7 (also known as TiPARP) is a mono-ADP-ribosyltransferase that acts as a critical negative regulator of AhR. AhR directly stimulates PARP7 gene expression. The resulting PARP7 protein carries out mono-ADP-ribosylation of the AhR molecule. This modification marks the AhR protein for proteolytic degradation [122,123]. The described mechanism of AhR activation is considered classical (canonical). Additionally, there are non-classical (non-canonical) AhR signal transduction pathways where the AhR–ligand complex heterodimerizes with proteins other than ARNT [124,125], such as Krüppel-like factor 6 (KLF6) and RelB [126,127] (Figure 1).

AhR is widely known as a ligand-dependent transcription factor, but it also possesses vital intrinsic, ligand-independent functions. AhR operates through significant ligand-independent mechanisms, collaborating with various cellular factors and non-canonical signaling pathways to regulate cellular proliferation, development, and baseline homeostasis [128,129,130]. Unliganded AhR regulates baseline cell growth. It achieves this by interacting with proteins that control the stability of mRNA. For instance, AhR can inhibit the nuclear export of an mRNA-destabilizing factor tristetraprolin (TTP), which consequently regulates cell growth rates and Transforming Growth Factor β (TGF-β) signaling [128]. Chaperone and co-chaperone proteins of the AhR multi-protein complex, including HSP90, p23, c-Src and XAP2, can themselves crosstalk with other signaling pathways [131].

AhR can be involved in other signal transduction pathways. These include the nuclear factor erythroid 2-related factor 2 (NRF2) signaling pathway, which is a master regulator of the body’s endogenous antioxidant defense system [132], as well as the estrogen receptor signaling pathway [67,89]; the hypoxia-inducible factor (HIF-1α) pathway [133,134]; and the Wnt pathway, which initiates a cascade of reactions regulating normal embryonic development [119]. The AhR signaling pathway also interacts with the NF-κB pathway, which is a crucial intracellular signaling mechanism in the regulation of inflammatory responses [4,110,135,136]. AhR promotes NF-κB activation by interacting with the RelA and RelB subunits of NF-κB [137,138,139]. AhR and NF-κB are involved in the formation of feedback loops within the framework of immune regulation, modulating each other’s signaling [140,141].

Acting as a transcription factor and affecting expression regulation networks, AhR is involved in a variety of physiological and pathological processes. One of its applications is the immune system and the regulation of inflammation. This is related to the role of environmental pollutants in the development of immunological diseases, including autoimmune disorders [4]. Furthermore, AhR controls the expression of several cytokines in immune cells and is involved in the regulation of innate and adaptive immune responses, as well as inflammation [5,6,7,8].

The AhR signaling pathway is associated with inflammatory markers such as cyclooxygenase-2 (COX2), tumor necrosis factor α (TNFα), matrix metalloproteinases (MMPs), early growth response protein 1 (EGR1), prostaglandin E2 (PGE2), microsomal PGE2 synthase (mPGE2S), NF-κB components including RelB and RelA, inducible nitric oxide synthase (iNOs), and IL8 [142].

## 4. Involvement of AhR in the Regulation of Immunity and Inflammation

The respiratory system is in direct contact with the environment, reacting with exogenous compounds and microorganisms in the inhaled air. The immune system in the lungs is designed, on the one hand, to resist harmless components of the air entering the lungs and, on the other, to mount an adequate response to invading pathogens. Among the exogenous compounds coming from the air are environmental pollutants that are ligands/agonists of AhR [143].

### 4.1. AhR-Dependent Regulation of Immune Cells

AhR is a key regulator of both links of the immune system in the respiratory tract, participating both in the development of innate and adaptive immune cells and in the regulation of their functions [144].

AhR is expressed constitutively or inducibly in most immune cells [4,73,145]. Thus, Th cell subpopulations differ in AhR expression. Specifically, Th17 cells express the highest levels of AhR; regulatory T cells, including FOXP3^+^ Tregs and type 1 regulatory T cells (Tr1), express AhR at lower levels, and naive CD4^+^ Th1 and Th2 T cells show virtually no AhR expression [146,147,148].

AhR has numerous effects on cells of the innate immune system, including macrophages, mast cells, and neutrophil granulocytes, as well as cells of the adaptive system, namely, B cells, antigen-presenting cells, Th1/Th2, Th17 cell subtypes, and Tregs [1,89,149,150,151].

#### 4.1.1. The Role of AhR in the Development of Immune Cells

AhR mediates mast cell differentiation and growth [152,153], DC functions [154], and intraepithelial lymphocyte functions [155]. In DCs, AhR activation operates monocyte differentiation into DCs. DC activation, costimulation, and antigen presentation are enhanced by activation by various AhR ligands [156,157].

In B cells, AhR activation inhibits the differentiation and development of murine B cells in a manner of impaired B cell lineage, preventing the differentiation of mature B cells into plasma cells and the production of early B cells and pro-B cells. AhR is highly expressed in Bregs and is involved in the regulation and immunosuppressive function of Bregs [156,157].

AhR plays a key role in regulating T cell differentiation. In CD4^+^ T cells, ligands from various sources enhance the differentiation of Tregs and Th17 cells via various activation mechanisms of AhR [1,158], Tr1 [159], and Th9 [160].

AhR activation modulates immune cell differentiation in favor of Tregs, which provides an immunosuppressive microenvironment [161]. AhR maintains a balance between Th17 and Tregs, including Tr1 and CD4^+^CD25^+^Foxp3^+^ Tregs [162]. AhR may be involved in the development of Th2 cells [163,164].

AhR controls the differentiation of monocytes into macrophages [25]. AhR promotes the transformation of macrophages into either an anti-inflammatory or pro-inflammatory phenotype [5,6]. These outlined effects of AhR ultimately lead to the disruption of the M1/M2 polarization balance, as well as the balance between Tregs and Th17 cells [5,6].

The differentiation of effector and regulatory T cells is controlled by antigen-presenting DCs, which are subject to AhR-mediated modulation. AhR controls DC differentiation and function by arresting progenitor differentiation and regulating antigen presentation in DCs [165,166].

AhR mediates MDSC mobilization in a CXCR2-dependent manner and promotes differentiation and immunosuppression of the MDSC subgroup polymorphonuclear myeloid-derived suppressor cells (PMN-MDSCs) [26]. AhR has also been described as a transcription factor critical for ILC2 differentiation [167].

Various AhR ligands are involved in immune cell development. These are dioxins (e.g., 2,3,7,8-tetrachlorodibenzo-p-dioxin and TCDD), which are present in the environment as a byproduct of industrial processes; endogenous ligands, such as prostaglandin G_2_ (PGG_2_), which is produced during inflammation from arachidonic acid; indole-3-carbinol, obtained by hydrolysis of sulfur-containing compounds from cruciferous plants; and kynurenine, an immunosuppressive molecule produced during tryptophan metabolism [158,168,169,170].

#### 4.1.2. The Role of AhR in the Regulation of Immune Cell Functions

AhR signals affect the formation of various types of immune responses.

In macrophages, AhR is involved in their transformation into a pro- or anti-inflammatory phenotype and activates macrophage-dependent cellular pathways, leading to the generation of anti-inflammatory cytokines IL-10 or IL-1β and TNF-α [4], and the inhibition of pro-inflammatory IL-6, IL-12, and TNF-α [171].

AhR activation in T cells stimulates the expression of the transcription factor FoxP3, which is critical for the differentiation and function of Tregs. Furthermore, AhR activation negatively regulates the expression of the transcription factor RORγt, which stimulates the differentiation of pro-inflammatory Th17 cells [158].

In Th17 cells, AhR also stimulates the production of IL-17A, IL-17F, IL-10, and IL-22 [4,5,6,7]. In Treg cells, AhR reduces the production of pro-inflammatory cytokines [4]. In addition, AhR signaling enhances the ability of Tr1 cells to produce IL-10 and IL-21 [171]. In Th2 cells, AhR can be involved in the production of cytokines such as IL-4, IL-5, and IL-13 [163].

In antigen-presenting DCs, AhR activation attenuates the induction of IL6 and IL12 cytokines, which promote the polarization of the pathogenic T cell subpopulation, and increases the DC transcription signature by activating CD80, CD86, and major histocompatibility complex II (MHC II) [4,89]. AhR signaling also stimulates IL-10 and IDO expression in DCs [171]. DCs can suppress Th1/Th2 effector responses and promote immune tolerance in response to signaling activated by IL-10, IL-27, or AhR ligands [40].

In B cells, AhR regulates an important transcriptional element in the immunoglobulin heavy chain gene, so that the production and secretion of immunoglobulins are impaired in an AhR-mediated manner [172]. For instance, activation of AhR by dioxin inhibits antibody production by B cells [171].

Previous research has identified species differences in the transactivation potential of AhR in the immunoglobulin heavy chain (IgH) locus [173]. A role of AhR has been demonstrated in the B cell subtype of CD5^+^ Innate-Like B Cells, which link adaptive and innate immunity [174]. However, some other B cell subtypes may be refractory to modulation by AhR [174]. Ligand-dependent AhR is thought to play a role in fine-tuning antibody production [175].

AhR controls the development and function of adult RORγt^+^ILCs [176,177,178]. Under certain pathological conditions, AhR plays a role in ILC2 effector function in the lungs by regulating IL-17A expression [179,180,181]. AhR is required for intestinal ILC3 maintenance and IL-22 production [176,178]. AhR prevents the differentiation of the innate ILC3 subpopulation into NK cells [182].

Thus, AhR participates in the regulation of signaling pathways involved in the differentiation and development of various immune cells (Table 1). AhR activation promotes the development and/or function of immune cells such as Th17, Tregs, ILCs, and monocytes (macrophages). Conversely, AhR activation suppresses the development and/or function of B cells, DCs, and NK cells.

## 5. Mechanisms of AhR Involvement in Immune Reactions and Inflammation

AhR can interact with other signaling pathways involved in regulating immune cell function, such as the genomic NF-κB, JAK-STAT, and HIF-1α pathways, and the non-genomic Src-STAT pathway. AhR also regulates immune cell development and function by direct binding to downstream target genes, such as FOX3, RORγt, and c-Maf. These interactions can lead to synergistic or antagonistic effects on the expression of genes critically important and required for immune and inflammatory responses.

### 5.1. AhR–NF-kB-Mediated Inflammation

AhR regulates the transcription and expression of inflammatory molecules, as evidenced by certain studies demonstrating AhR’s involvement in upstream inflammatory processes [183,184]. In this context, AhR interacts with endogenous molecules such as the transcription factor NF-κB.

NF-κB is currently considered to be one of the most influential factors in immune responses and inflammation control. NF-κB is a central mediator of pro-inflammatory gene induction and operates in both innate and adaptive immune cells [183,184]. NF-κB is induced by many pro-inflammatory ligands, such as inflammatory cytokines, bacterial products, reactive oxygen species (ROS), stress inducers, and a wide range of drugs [183,184].

In turn, it affects the expression of multiple target genes, including inflammatory cytokines (TNF-α, IL-1β, IL-2, IL-6, IL-8, IL-12), chemokines, and additional inflammatory mediators in various types of innate immune cells. These inflammatory mediators can directly participate in the induction of inflammation or act indirectly by promoting the differentiation of inflammatory T cells [184,185].

NF-κB affects inflammation not only by directly increasing the production of inflammatory cytokines, chemokines, and adhesion molecules, but also via regulating cell proliferation, apoptosis, morphogenesis, and differentiation [184]. In addition, NF-κB is involved in the regulation of inflammasomes as a central mediator of the priming signal for the activation of the NLRP3 inflammasome and induces transcriptional expression of NLRP3 and pro-IL-1β in response to various ligands of PRRs and cytokines [184,185].

The AhR-dependent signaling pathway actively interacts with NF-κB via signal transduction [4,110,135,136]. AhR signaling promotes NF-κB activation due to AhR interactions with the subunits RelA and RelB [137,138,139]. The RelB-AhR dimer binds to the RelBAhRE response element on the promoter of genes such as IL-6 or IL-8 [135,141,186].

RelB/AhR complexes also target NF-κB binding sites, which are preferentially recognized by RelB/p52, as well as XREs, a consensus sequence for the AhR-ARNT dimer, and NF-κB consensus sequences [135,141]. Interaction with RelB synergistically induces the transcription of genes such as IL-6 and IL-8 in DCs or macrophages [135]. AhR may be involved in NF-κB signaling, which is activated by classical inducers such as LPS [141].

AhR and NF-κB can be involved in regulatory feedback loops, which are important in immune regulation processes. NF-κB induces AhR expression, but AhR can also regulate NF-κB signaling [140,141]. Experiments in AhR (^−/−^) mice have shown that AhR can control macrophage polarization via regulating NF-κB, and AhR depletion increases NF-κB-dependent macrophage activation in vitro [187].

Thus, NF-κB trans-represses AhR activity, and, concurrently, AhR interferes with the binding between I-κB and NF-κB sequences, thereby affecting their function in resolving inflammation.

### 5.2. AhR and Signal Transducer and Activator of Transcription (STAT)

#### 5.2.1. AhR and JAK-STAT

Differentiation of Th17 cells involved in inflammation is triggered by an intracellular signaling cascade including cytokine-dependent phosphorylation and activation of STAT3 [188,189,190]. This occurs as a result of the binding of the cytokine to its receptor, followed by the homodimerization of the signaling β-receptor gp130 [189] and phosphorylation of the Signal Transducer and Activator of Transcription (STAT3) via the activation of gp130-associated Janus kinases (JAKs), in particular JAK1 [190]. After phosphorylation, STAT3 is translocated to the nucleus and induces the transcription of genes responsive to pro-inflammatory cytokines (e.g., IL-6) [190].

The JAK-STAT signaling pathway is considered one of the central communication nodes in cellular functioning. The JAK/STAT pathway is involved in processes of cell division, cell death, tumorigenesis, and immunity [191,192]. More than 50 cytokines and growth factors have been identified within the JAK/STAT signaling pathway, activating the pathway [191]. Activated AhR can modulate the JAK/STAT signaling by regulating the gene expression of the cytokines IL-2, IL-10, IL-21, and IL-22 [131,192].

The JAK/STAT pathway also triggers transcription of the IDO gene, which encodes the key enzyme indoleamine 2,3-dioxygenase (IDO) in the kynurenine pathway of tryptophan degradation [131,192]. Tryptophan metabolism via the kynurenine pathway results in the formation of kynurenine (KYN) and its metabolite kynurenic acid (KYNA), which act as AhR agonists [193,194].

#### 5.2.2. AhR and Src-STAT3

Indirect interaction between STAT3 and AhR can be accomplished non-genomically, involving the co-chaperone complex AhR-Src. As described previously, Src is a cytosolic kinase within the latent AhR protein complex. Upon ligand binding to AhR, Src dissociates from the complex and gets activated. Then, Src exerts its kinase activity, stimulating different signaling pathways and phosphorylating a wide range of target proteins, including IDO1 [195].

#### 5.2.3. Direct Interaction of AhR with STAT Proteins

Ligand-activated AhR can directly interact with STAT3 proteins to induce or repress the transcriptional activity of the AhR and IDO genes [192]. Following JAK-mediated phosphorylation, the activated STAT3 translocates to the nucleus and can interact first with a putative STAT motif in the AhR promoter region, thereby maintaining AhR gene activation and expression, and, second, with the IDO promoter [193,196].

Furthermore, AhR can attenuate lipopolysaccharide (LPS)-induced expression of pro-inflammatory IL-6 in macrophages by forming a protein complex with STAT1. This AhR-STAT1 complex suppresses IL-6 promoter activity and thereby reduces IL-6 production [197].

### 5.3. AhR and Hypoxia-Inducible Factor 1-Alpha (HIF1α)

HIF1α is a transcription factor from the bHLH/PAS class I protein superfamily, which is active under low-oxygen conditions and mediates the cellular response to hypoxia. HIF-1α expression depends on cellular oxygen levels. Under normal oxygen conditions, HIF-1α gets rapidly ubiquitinated and degraded via the proteasomal pathway [133].

Under hypoxic conditions, HIF-1α is stabilized or protected from proteasomal degradation; this enables it to translocate into the nucleus and form heterodimeric complexes with ARNT, regulating the expression of multiple hypoxia-responsive genes [133,171]. In addition to activation under low-oxygen conditions, HIF1α activity can also be induced by cytokines, growth factors, ROS, or microbial products [171]. HIF-1α is able to control the balance between CD4^+^ T cell differentiation into Th17 cells and Tregs under both normoxic and hypoxic conditions, promoting Th17 cell differentiation through transcriptional activation of RORγt and, conversely, suppressing Treg cell development through FoxP3 binding [133].

AhR is also a class I bHLH/PAS factor. AhR and HIF-1α, having structural similarities, heterodimerize with ARNT and bind to similar DNA response elements, which promotes direct interaction between the proteins [133]. Furthermore, a growing body of research suggests crosstalk between the AhR and HIF-1α pathways, both of which control many biological functions, including influences on innate and adaptive immunity [133,192].

An example of direct crosstalk between the AhR and HIF-1α pathways is their involvement in Tr1 cell differentiation. Under conditions where HIF1α is metabolically activated, HIF1α controls the early stages of Tr1 cell development, while hypoxically activated HIF1α suppresses Tr1 cell differentiation. AhR controls later stages of Tr1 cell development [133]. Under these circumstances, AhR controls metabolic reprogramming of Tr1 cells by directly affecting metabolism-related target genes and promoting HIF1α degradation, thereby inhibiting HIF1α-operated metabolic reactions [159].

Depending on the context, HIF1α and AhR can either positively interact or exert opposing effects on the differentiation and activity of certain T cell types [133,171]. Both AhR and HIF-1α pathways can promote Th17 cell lineage differentiation. DCs expressing HIF-1α can promote Th1 differentiation, whereas AhR blocks Th1 cell differentiation [133].

### 5.4. AhR and Forkhead Box Protein 3 (FOXP3)

FOXP3 is the major transcription factor in Tregs, controlling the expression of multiple genes that determine the differentiation and functions of Tregs [198]. However, only a small fraction (<10%) of Treg-associated genes are directly linked to FOXP3, and FOXP3 alone is insufficient for the complete specification of the Tregs program [198].

AhR, a FOXP3 agonist, acts as one of the additional transcription factors controlling Tregs’ identity. The AhR-regulated module promotes the generation of Tregs and enhances their suppressive function [198].

AhR stimulates the expression of Foxp3^+^ Tregs through several molecular mechanisms. Some of these mechanisms are mediated by synergistic effects with TGFβ and recruitment of additional transcriptional regulators, while others operate directly [198]. First, AhR activation enhances Aldh1a1 expression in DCs, which stimulates the secretion of retinoic acid (RA), and in T cells, RA binds to RAR and RXR, which, in turn, bind to the FOXP3 promoter. Second, AhR activation induces KYN production in DCs in an IDO-dependent manner. KYN also acts as an endogenous AhR ligand and stimulates FOXP3 expression [146].

Furthermore, AhR activation directly affects FOXP3 expression by triggering FOXP3 demethylation and transactivating its promoter in FOXP3^+^Treg cells [199,200].

The interaction of Foxp3 with AhR determines the role of AhR in regulating the differentiation and function of FOXP3^+^Treg cells, which are involved in maintaining immunological tolerance and preventing excessive immune responses [5,201], as well as in the control of Th1 and Th17 effector cells [202].

IL-6-dependent STAT3 activation suppresses TGF-β-induced FOXP3 expression and promotes the differentiation of naive CD4^+^ T cells into Th17 cells, simultaneously suppressing the development of Tregs [188]. AhR, through the c-Maf signaling pathway, modulates Tr1 cell functions which operate Tr1 cell differentiation and IL-10 and IL-21 expression [146,203,204]. AhR inhibits STAT1 activation, which mediates the inhibitory effect of interferon-γ (IFN-γ) on FOXP33^+^ Tregs [199,200].

### 5.5. AhR and Musculoaponeurotic Fibrosarcoma Transcription Factor c (c-Maf)

Transcription factor c-Maf plays an important role in the immune system, affecting almost all types of immune cells [205]. The c-Maf controls the differentiation of Th2 cells and affects the polarization and function of macrophages [206]. In Th17 cells, c-Maf is induced by TGFβ, stimulating the production of IL-17 and suppressing the secretion of IL-22 [207].

AhR, via the c-Maf signaling pathway, modulates Tr1 cell function by regulating Tr1 cell differentiation and IL-10 and IL-21 expression [146,203,204]. First, AhR ligands bind to AhR and transactivate the promoter of the corresponding cytokine via c-Maf, providing Tr1 cell proliferation. Second, the same AhR and c-Maf complex can transactivate the promoter of the corresponding cytokine and mediate the suppressive function of Tr1 cells. In addition, AhR triggers transactivation of the *Gzmb* promoter, which stimulates granzyme B expression, thereby promoting the suppressive activity of Tr1 cells [146,203].

### 5.6. AhR and Retinoic Acid-Related Orphan Receptor γt (RORγt)

The RORγt isoform is one of the RORγ isoforms, and is expressed in certain lymphoid cell types and detected in immune cells during thymopoiesis and lymphopoiesis [208,209]. RORγ is encoded by *RORC*. RORγt is typically expressed by ILCs (primarily by group 3) and CD4^+^ T cells, including Th17 cells and Tregs. In Th17 cells, RORγt is a key transcription factor controlling their differentiation and IL-17 expression [206,210]. To stimulate differentiation of naive CD4^+^ T cells into Th17, RORγt interacts with a cocktail of cytokines (IL-6, IL-21, IL-23, IL-1β, TGF-β), as well as with STAT3 and some other transcription factors [198,206].

AhR has been shown to be required for IL-22 production by T cells and ILC3 [208,209]. Synergistic action between AhR and RORγt to activate IL-22 expression was observed in the EL4 thymoma cell line and in primary T cells [176]. Physical association between AhR and RORγt was found in HEK293T cells [176].

AhR itself does not bind to the *Il22* locus. RORγt is required for AhR recruitment to the *Il22* locus [176]. The precise contribution of the interaction between AhR and RORγt to the regulation of *Il22* transcription is not fully understood. RORγt is thought to facilitate AhR binding at the chromatin level. With the assistance of RORγt, AhR can achieve enhanced DNA binding activity; as a result, it directly binds to AhRE at the *Il22* locus, inducing transcription. Alternatively, AhR can indirectly bind to ROR response elements (ROREs) through interaction with RORγt [211].

The mechanisms by which AhR stimulates IL-22 expression in Th17/22 cells and ILC3 are not yet fully understood. In pulmonary ILC2, AhR interacts with RORγt to operate IL-22 expression. Unlike Th17 cells, STAT3 does not stimulate AhR expression in ILC3 but can interact with AhR to increase *IL22* expression [212]. AhR activation can negatively regulate RORγt transcription factor expression [158,213] via the JAK 1-STAT3 pathway; in this manner, activation of this pathway, mediated by the IL-6–IL-6R axis, stimulates RORγt transcription [214].

### 5.7. AhR and TGF-β

TGF-β is a multifunctional cytokine that regulates crucial cellular processes, including proliferation, differentiation, and immune function. TGF-β1 can upregulate AhR expression in naive T cells [215]. TGF-β signaling can disrupt the AhR/ARNT complex (essential for AhR target gene transcription) by utilizing downstream effectors like Smad3 and HIF-1α [216]. AhR often acts as a physiological repressor of TGF-β activity [128,217,218]. AhR and TGF-β signaling pathways form a complex, reciprocal regulatory network that heavily dictates immune responses, tissue inflammation, and fibrosis. Together, they maintain immune homeostasis but can become pathological when unbalanced [219,220,221,222,223]. The AhR and TGF-β pathways interact closely in the lungs, controlling the balance between lung tissue repair and destructive inflammation [224,225]. TGF-β and AhR can drive inflammation or exert immunosuppressive and tissue-protective effects [215,220,226].

The AhR and TGF-β axis is a major focus in fibrotic diseases. Ligand-activated AhR can antagonize TGF-β1-induced fibroblast activation, effectively dampening excessive collagen and gene expression [227,228]. The crosstalk between the AhR and TGF-β signaling pathways is a primary mechanism for the induction, stabilization, and suppressive function of FoxP3+ Tregs [198]. Naive CD4^+^ T cell activation triggers the release of TGF-β, which initiates the expression of the *Foxp3* gene and simultaneously upregulates AhR expression on the cell surface [211,229,230,231]. AhR activation by ligands such as TCDD or ITE in the presence of TGF-β promotes the differentiation and stability of immunosuppressive Tregs [203,232,233]. A critical transcriptional network that regulates T cell differentiation from AhR is TGF-β and c-Maf. Together, they drive the generation of immunosuppressive and anti-inflammatory cells [229]. AhRThis combined pathway drives the differentiation of Tr1 cells and maintains Foxp3^+^ Treg cells, ultimately resulting in the suppression of excessive inflammatory responses [204,234]. AhR, TGF-β and RORγ or RORγt form a critical regulatory axis in Th17 cell differentiation and fibrosis control [192,215,218,235].

### 5.8. The IL-6-STAT-3-IDO-Kyn-AhR Autoinflammatory Cycle

IL-6 is a frequent partner of AhR in inflammatory responses. Activated AhR primarily induces expression of pro-inflammatory IL-6 via the canonical pathway [150]. In the inflammatory environment induced by IL-6, the IL-6 receptor (IL6R) is activated. Following JAK-mediated phosphorylation, STAT3 becomes active and translocates into the nucleus, where it interacts either with a putative STAT motif in the AhR promoter region, thereby maintaining AhR gene activation and expression, or with the IDO promoter [236].

IDO is a key enzyme in the kynurenine pathway of tryptophan degradation [131,192]. Tryptophan metabolism via the kynurenine pathway results in the formation of KYN and its metabolite KYNA, which act as AhR agonists [193,194]. Thus, AhR induces the formation of its ligands through a positive feedforward loop involving IDO1. Activated AhR translocates to the nucleus and binds to the XRE in the *IL-6* gene promoter. As a result, IL-6 is synthesized and released from the cell. The binding of IL-6 to IL-6R terminates this autoinflammatory cycle maintained by AhR [193,194].

IDO1 can maintain its own expression via the AhR–IL-6–STAT3 autocrine signaling loop. IDO1 is also activated by pp60src, which dissociates from the AhR-inactivating complex and phosphorylates IDO1, terminating the inflammatory cycle [237]. In parallel, STAT3 maintains AhR expression, thereby stimulating IL-6 expression and, consequently, creating a self-perpetuating autoinflammatory cycle.

Furthermore, STAT3 activation can stimulate the non-canonical NF-κB pathway and lead to the induction of IDO transcription through direct binding to the IDO promoter region in DCs and MDSCs [238,239]. In addition, the non-canonical AhR signaling pathway may be involved in NF-κB-mediated inflammation [191,193,194].

KYN and KYNA, acting as AhR agonists, promote T cell differentiation and thereby affect immune system activity and inflammation [158]. The importance of these connections to tryptophan metabolism is evidenced by their key roles in differentiating naive CD4^+^ T cells into Th17 cells or Tregs. KYN induces the expression of FoxP3, which is important for the differentiation and function of immunosuppressive Tregs. Furthermore, KYN inhibits RORγt, which promotes the differentiation of pro-inflammatory Th17 cells [240]. Kynurenic acid also has significant anti-inflammatory effects due to its ability to inhibit the differentiation of CD4^+^ T cells into the Th17 phenotype and suppress the release of IL-4 and IL-23 from activated monocytes [92,241].

## 6. Regulatory Role of AhR in Lung Inflammation Models of Experimental Animals

Inflammation in the lungs is a tightly regulated process involving complex interactions between cells imported from the blood, including platelets, neutrophils, eosinophils, mast cells, and lymphocytes; lung cells, including type I and II pneumonocytes, endothelial cells, and Clara cells; alveolar and intravascular macrophages; stromal interstitial cells such as mast cells; interstitial macrophages; fibroblasts; and myofibroblasts. Acute pneumonia begins with the migration of neutrophils, followed by the production of chemokines to organize the formation of granulation tissue based on cellular matrix, fibroblasts, endothelial cells, and leukocytes.

Chronic inflammation occurs when acute inflammation is incompletely resolved. It primarily affects macrophages and lymphocytes. The development of a pathological immune response in the airways begins with the initiation of immune responses by DCs in the airway epithelium, which capture and process pathogens, toxins, allergens, and other substances, presenting them on MHC class II molecules. Activated DCs then migrate to the lymph nodes, where they stimulate allergen-specific naive T cells, leading to clonal expansion and differentiation into Th2 cells, which secrete inflammatory cytokines. In chronic lung inflammation, pro-fibrotic and immunoregulatory cytokines of Th2 cells (IL-4, IL-5, IL-9, and IL-13) [242] predominate, playing a key role in the organization of chronic inflammation and structural changes in the airways. Cytokines such as IL-10 and TGF-β, produced by lymphocytes, play an active role in suppressing the production of inflammatory cytokines and the immune response in the lungs [243,244].

Experimental studies illustrate the potential differences that can arise in the immune and inflammatory systems of the lungs and other respiratory organs after activation of AhR by different agonists.

Activation of AhR by the prototypical agonist TCDD increased the expression of inflammatory cytokines (TNF-α, IL-1β, MCP-1), mucin 5AC, and several matrix metalloproteases (MMP-12, MMP-13) in mouse lungs, which could have been associated with increased lung infiltration by neutrophils and macrophages [245]. These effects were found to be dependent on AhR activation via XREs in the promoter/enhancer regions of these genes [245].

In mice with hyperoxic lung injury, omeprazole, a proton pump inhibitor with affinity for AhR, attenuated lung inflammation by increasing the expression of cytochrome P450 enzymes, particularly CYP1A1. Both neutrophil infiltration and MCP-1 expression were reduced compared to animals receiving placebo [246]. This protective effect was AhR-dependent, since similar effects were not revealed in C57Bl/6 mice expressing low-affinity AhR [246].

Activation of AhR by TCDD reduces airway inflammation, for example, in rodents with asthma, by regulating the production and secretion of Th2 cytokines such as IL-4, IL-5, and IL-15 [245,247].

AhR expression was shown to limit pro-inflammatory cyclooxygenase-2 (COX-2) and prostaglandin production induced by cigarette smoke in mice by maintaining RelB expression [248]. However, treatment of human lung fibroblasts with AhR antagonists in the presence of cigarette smoke extract inhibited AhR nuclear translocation, as well as COX-2, mPGES-1, and prostaglandin E production [249].

Exposure of experimental mice to cigarette smoke for 2 weeks or 4 months significantly increased lung inflammation, and AhR-deficient mice had higher neutrophilia after smoke exposure [250]. In mice exposed to cigarette smoke, AhR suppressed pulmonary neutrophilia in response to acute [250,251,252] and subchronic exposure [253].

In AhR knockout mice in a cigarette smoke-induced COPD model, lack of AhR expression resulted in reduced lung cell survival, primarily due to mitochondrial dysfunction and decreased levels of antioxidant regulatory proteins (MnSOD and CuZn-SOD) [254]. This suggests that cigarette smoke-induced structural cell loss in the lung, caused by changes in AhR expression, may significantly promote the development of inflammatory lung diseases in smokers [254].

The role of AhR in the Th17/Treg cytokine imbalance has been demonstrated in an experimental COPD model in C57BL/6 mice exposed to cigarette smoke and lipopolysaccharides during COPD exacerbation [255]. Herein, it was shown that the inflammatory response was characterized by an increase in Th17 levels and a decrease in the number of IL10-positive cells, despite the differentiation of Tregs [255].

AhR may be involved in the regulation of inflammation through its interaction with the p65 subunit of NF-κB. However, the results of this interaction are ambiguous. Some studies show that AhR has pro-inflammatory activity, while others suggest that AhR may have anti-inflammatory effects. A study examining the effects of AhR and ARNT on the activation of p65 and two differentially regulated chemokines, IL-8 and CCL5, in human bronchial epithelial cells revealed that constitutively active AhR, in the unbound state, suppressed p65 activation, while ARNT apparently affected the functioning of activated p65. Ligand-activated AhR suppressed the IL-8 and CCL5 response by other agents, but AhR ligands alone induced IL-8 response when given at sufficiently high concentrations, highlighting the duality of AhR in regulating inflammation [256].

## 7. Regulatory Role of AhR in Lung Inflammatory Allergic Disease Models of Experimental Animals and In Vitro Studies

Allergic diseases are closely linked to inflammatory processes. Experiments on mice have shown that allergic airway inflammation is more active in *AhR*^−/−^ and CYP1B1^−/−^ mice than in wild-type mice [257]. In *AhR*^−/−^ mice, cockroach allergen causes more acute lung inflammation compared to wild-type mice, and this inflammation is suppressed by the AhR agonist TCDD [258].

In a murine *asthma* model induced by cockroach extract, AhR signaling is enhanced, as evidenced by increased AhR, CYP1a1, and CYP1b1 mRNA expression. The AhR antagonist CH223191 inhibits AhR signaling and increases the expression of M1 macrophage markers [259]. Deficient AhR signaling affects immune and non-immune cells, such as neutrophils, macrophages, and fibroblasts in the lung, leading to increased lung inflammation upon exposure to tobacco smoke or lipopolysaccharide in rodents [250,260].

Allergic diseases are typically described as abnormal conditions primarily initiated by immunoglobulin E (IgE)-dependent mechanisms. However, IgE binding to primary allergens triggers allergic reactions in approximately 50% of patients. Other non-primary allergens can also trigger allergic reactions and activate various immune cells [261,262].

Allergic inflammatory responses represent a complex organization of immune cells such as eosinophils, epithelial cells, and T lymphocytes, and are operated via Th2 cell-dependent signaling pathways [150]. The allergen-induced immune response begins when the allergen via antigen-presenting cells is recognized as an antigen by naive T cells through the MHC class II receptor. After initial allergen exposure, naive T cells differentiate into Th2 cells producing IL-4 and IL-13, which activate B cells, promoting their differentiation into plasma cells, followed with the activation of IgE production. IgE binds to mast cells, activating their degranulation and promoting antigen presentation to other immune cells [150]. Re-exposure to the allergen triggers cross-linking of laden high-affinity IgE receptors on the mast cell surface, inducing an acute-phase immune response [164].

Afterwards, in the late-response phase, Th2, eosinophils, and mast cells secrete pro-inflammatory mediators that can activate bronchial hyperreactivity and allergic inflammation [263].

The lung epithelium serves as a barrier against environmental factors and responds to allergens with a cascade of immune responses, which can lead to inflammation. Activation of lung epithelial cells by an allergen triggers the production of Th2 cells through the release of specific cytokines and chemokines [164]. Th2-mediated allergen-specific responses regulate Treg cells, suppressing them through the secretion of IL-10 and TGF-β [164,264,265,266].

Numerous studies have proven the role of IgE and eosinophils in the pathogenesis of allergic inflammation, but the role of other immune cells in allergic reactions has not yet been sufficiently studied [267].

The AhR signaling pathway, involved in immune regulation, contributes to the regulation of allergic airway inflammation. AhR is associated with allergic airway inflammation induced by environmental pollutants [268,269,270,271].

The functional linkage between AhR and barrier defense/inflammatory signaling is also observed during allergen provocation. Inhalation of allergens such as pollen, dust mites, animal dander, and mold triggers an immune reaction leading to a pathological immune response in the airways. Potential inhalant allergens include numerous AhR ligands. These are components of organic and inorganic dust as well as chemicals, which pollute the environment, partly owing to industrial production [272]. Among such chemical compounds are known PAHs (air pollutants, particulate matter in the environment and particles from diesel engine exhaust gases, cigarette smoke, etc.), and dioxins (TCDD) [273].

In response to potential allergens (AhR ligands), AhR expression itself and the downstream AhR signaling pathway play different roles in diverse immune cells. This has been demonstrated in various experimental models of allergic diseases using various potential allergens (AhR ligands).

In a model of allergic asthma, exposure to cockroach extract (CRE B46) resulted in M1/M2 macrophage polarization [259], while exposure to urban dust particles (SRM 1649b) resulted in increased Th17 polarization [274]. In a model of dust mite-induced allergic asthma, exposure to BP or 2.5 μm particulate matter (PM 2.5) resulted in increased secretion of cytokines in Th2, but not Th1 or Th17 [275,276].

In an ovalbumin-sensitized allergic asthma model, indoxyl-3-sulfate (I3S) regulated Th2 differentiation [277], while 4-nonylphenol Indeno [1,2,3-cd]pyrene increased Th2 cytokine secretion [269]. In an ovalbumin allergy model, ovalbumin aerosol exposure resulted in T cell activation by DCs [163]. In a non-eosinophilic asthma model, TCDD increased Th17 differentiation and decreased Treg differentiation [278].

In a cockroach-sensitized mouse model, PM2.5 exposure increased Th17 differentiation and decreased FOXP3^+^ regulatory T cell differentiation [279]. In a peanut allergy model, indole-3-carbinol (I3C) increased the number of CD11c^+^ and CD103^+^ MHC-II^+^ cells [280]. In mast cells derived from the bone marrow of *AhR*^−/−^ mice, FICZ exposure resulted in maturation and activation of mast cells and secretion of IL-6 and IL-17 [277,281], while in a peanut allergy model, FICZ had no effect on Tregs [282].

In a cockroach extract-induced murine asthma model, AhR signaling is enhanced, as evidenced by increased AhR, CYP1a1, and CYP1b1 mRNA expression. The AhR antagonist CH223191 inhibits AhR signaling and increases the expression of M1 macrophage markers [259]. In *AhR*^−/−^ mice, cockroach allergen induces more acute lung inflammation compared to wild-type mice, and this inflammation is suppressed by the AhR agonist TCDD [258].

Experiments in mice also demonstrated that allergic airway inflammation was more active in *AhR*^−/−^ and CYP1B1^−/−^ animals than in wild-type mice. CYP1B1 deficiency resulted in increased expression and activity of CYP1A1 in lung epithelium cells and increased availability of KYNA, the AhR ligand, after allergen exposure. Thus, there is a functional link between the AhR signaling pathway and barrier protection, as well as inflammation following allergen provocation. Differential expression of CYP1 and AhR signaling in lung epithelium plays an immunoregulatory role in protecting against allergic airway inflammation [257].

The lipocalin family of proteins, found in cow and goat milk, act as potential respiratory allergens [283,284]. The lipocalin beta-lactoglobulin, acting as an allergen, primes human mast cells for degranulation, promotes quercetin-dependent activation of AhR, and affects Cyp1A1 expression in the lungs [284]. The authors demonstrated a previously unknown role of the milk protein beta-lactoglobulin in the transport of AhR ligands to immune cells and suggested that beta-lactoglobulin is necessary for efficient ligand transport and AhR pathway activation in vivo. This may exert an immunoregulatory effect and lead to robust immune system activation and protection against allergic sensitization [284].

In a mouse model of atopic asthma, targeting AhR with appropriate ligands has been shown to inhibit Th2 cell differentiation, reduce the Th2 response, and suppress disease progression [277,278,285].

A recent study in a mouse model of asthma induced by co-exposure to BaP and the major dust mite allergen Der f 1 revealed that the AhR signaling pathway got activated and regulated oxidative stress and cytokine release caused by allergen co-exposure in epithelial cells of the airway [275,286]. Activated AhR enhances inflammation via the RhoA-TGFβ1 pathway. Concurrently, the combination of BaP and Der f 1 significantly increases ROS production in bronchial epithelial cells and induces increased secretion of inflammatory cytokines in Th2 cells (IL-25, IL-33, TSLP), leading to increased airway hyperreactivity and excessive mucus production. Thus, BaP and Der f 1 act synergistically, exacerbating allergic airway inflammation [275,286].

Another study demonstrated that in alveolar epithelial cells, AhR played an important role in protecting against ROS production, NLRP3 inflammasome activation, and Muc5ac expression induced by cockroach allergen [287]. Cockroach allergen can induce autophagy, which is critical for controlling the release of pro-inflammatory mediators, Th2-associated airway inflammation, and, subsequently, the development of allergic asthma. AhR regulates allergen-induced autophagy either through controlling the release of TGF-β1 or through AhR-regulated autophagy-associated genes, and thus promotes the protective mechanism of the AhR signaling pathway against allergic airway inflammation [288]. However, the exact mechanisms of AhR signaling in the regulation of allergic airway inflammation and its role are not fully understood [288].

Concluding the review of data on the role of AhR signaling in lung inflammatory diseases obtained in models of experimental animals, it is necessary to point out that AhR signaling can vary significantly between rodents and humans. There are differences in ligand binding affinity associated with differences in the interaction of the ligand binding domain (LBD) with chemical agents [289]. The prototypical AhR ligand TCDD is highly toxic in mice. The mouse AhR (*m*AhR) binds TCDD with up to a 10-fold higher affinity than the human AhR (*h*AhR) [290,291]. The *h*AhR often displays greater relative affinity for specific dietary and endogenous molecules compared to the *m*AhR [290].

Even when a ligand successfully activates both receptors, the functional output often differs. The transactivation domains (TADs) of mice and humans share relatively low sequence identity. This disparity leads to variations in the recruitment of essential coactivator proteins and differences in the subdomains utilized for gene activation [292]. Besides this, the metabolic clearance of AhR agonists varies wildly. For instance, the half-life of TCDD in rodents is roughly 12 to 31 days, whereas its clearance in humans ranges from 6 to 14 years [293]. Therefore chronic, low-level AhR activation produces distinct physiological consequences in humans that are difficult to model in short-lived, fast-metabolizing murine models [293].

Profound structural and functional differences in AhR signaling between mice and humans pose major translational hurdles in toxicology, immunology, and pharmacology. These species-specific disparities necessitate careful interpretation when extrapolating animal data to human physiology and drug development [289,294].

## 8. Regulatory Role of AhR in Inflammatory Lung Diseases in Humans

Clinical studies have shown that the main non-infectious inflammatory lung diseases, in which the role of AhR has been proven, are chronic obstructive pulmonary disease (COPD), asthma, idiopathic pulmonary fibrosis, interstitial lung diseases (ILDs), and occupational lung diseases.

Lung inflammation is often triggered by AhR activation by specific environmental factors. Key factors causing chronic lung disease in humans include air pollution and PM, environmentally resistant free radicals (ROS), diesel exhaust particulates, cigarette smoke, industrial chemicals, TCDD, and heavy metals [271].

Other important risk factors for the development of inflammatory lung diseases include exposure to organic and inorganic dust, chemicals and fumes, and indoor air pollution in areas used for cooking or biomass heating [10,295].

Cigarette smoke contains thousands of dangerous toxic compounds, such as carbon monoxide, carbon dioxide, hydrogen cyanide, ammonia, nitrogen oxides, formaldehyde, benzene, arsenic, acrolein, and many others; in addition, the tar emitted from tobacco combustion products contains large amounts of PAHs, such as BaP, and halogenated aromatic hydrocarbons, which are potent AhR agonists. Similar to cigarette smoke, burning biomass as fuel produces smoke rich in PAHs. Air pollution is associated with PM, namely fine PM (PM2.5), and traffic-related air pollution, which adsorb PAHs and dioxin-like compounds, allowing them to activate AhR upon inhalation. PM-mediated induction of AhR-dependent genes *CYP1*, *AKR*, *NOX*, and *COX-2* can be a source of ROS, leading to excessive ROS production and oxidative stress. Environmentally resistant free radicals are produced during the incomplete combustion of organic materials. They activate AhR in Clara cells of the airways, causing Th17-mediated lung injury and airway hyperresponsiveness. Diesel exhaust particles contain high levels of PAHs, which activate AhR receptors. Industrial chemicals, namely dioxin-like compounds (TCDD), such as Agent Orange, also activate AhR. Heavy metals, including cadmium, arsenic, lead, and chromium, can cause oxidative stress, leading to the production of endogenous AhR ligands and, consequently, receptor activation [1,150,271,272,295,296,297,298,299,300].

Enhancement of AhR signaling by such ligands may play a role in systemic inflammation, potentially contributing to the development of inflammatory diseases [301,302].

### 8.1. Chronic Obstructive Pulmonary Disease (COPD)

Some of the risk factors for COPD are cigarette smoking, inhalation exposure to organic and inorganic dust, and chemicals, which pollute the environment or which are formed during industrial production [272,295,303].

Chemicals found in cigarette smoke are major contributors to lung inflammation and related pathological processes, in part due to mediating the effects of AhR on the immune system [143].

The pathogenesis of COPD is determined by diverse cell populations that express AhR. Cigarette smokers have elevated levels of CD8^+^ T lymphocytes [304]. CD8^+^ and CD4^+^ T cells are found in the airways and lung parenchyma in COPD; in this regard, disease severity is associated with an increase in both of these cell populations [305,306].

In patients with COPD, the number of these cell types is even greater compared to smokers without COPD [307]. Th17 cells and IL-17 in COPD have been described as directly involved in disease progression and in the development of bacterial infections, which lead to COPD exacerbation [255]. Th1, Th2, and Th17 cells, as well as AhR-expressing Tregs, are also associated with COPD pathogenesis [306].

Tregs, as well as the anti-inflammatory interleukins they contain, have been shown to be important in preventing the development and progression of COPD [308,309]. In patients with mild to moderate COPD, a comparison of local and systemic responses revealed that intracellular signaling for the Th17 response was manifested in the early disease stages. In mild COPD patients, Th17 markers were detected in lung samples, while in moderate COPD patients, they were revealed in blood samples. Despite increased levels of Treg differentiation markers at various stages of COPD, both in the lungs and in blood samples, patients in the late stages of the disease showed decreased IL10 levels [310].

AhR is a major regulator of the macrophage inflammatory response, which is critical for the development of COPD, as there is a positive correlation between macrophage recruitment to the airways and disease severity [1]. Neutrophils, mast cells, lung epithelial cells, and lung fibroblasts expressing AhR [1] are also involved in the pathogenesis of COPD.

AhR activation does not directly affect neutrophils, but they are recruited to the inflamed lung after exposure to cigarette smoke [245]. Cigarette smokers have increased levels of pulmonary neutrophils and macrophages [304]. In patients with COPD, the number of pulmonary neutrophils and macrophages increases even more compared to smokers without COPD [307]. Increased numbers of mast cells are detected in the lavage and lung tissue of smokers, and AhR activation in mast cells leads to IL-17 production [281,311].

Activation of AhR in lung epithelial cells by TCDD or BaP results in changes in immunomodulatory genes, including IL-8 and certain genes involved in interferon and retinoic acid signaling, as well as mucin 5AC (MUC5AC) [312,313].

Finally, AhR regulates the inflammatory response in lung fibroblasts by attenuating tobacco smoke-induced COX-2 and prostaglandin production via the regulation of RelB [248].

AhR exerts ligand-specific effects on the lungs, which can both exacerbate and attenuate COPD. On the one hand, AhR activation can exacerbate COPD. Dioxins and PAHs in tobacco smoke contribute to the pathogenesis of COPD, causing inflammation, exerting toxic effects on the lungs, increasing the expression of mucin 5AC and matrix metalloproteinases, and eventually damaging ciliated cells, Clara cells, and alveolar macrophages [245,314,315,316,317].

Symptoms of chronic bronchitis have been observed in smokers and patients with Yusho oil disease (Japan) caused by the consumption of rice bran oil contaminated with dioxins and polychlorinated biphenyls [318,319,320]. On the other hand, AhR acts as a critical negative regulator, suppressing pulmonary neutrophilia and inflammation, particularly in response to environmental stressors such as cigarette smoke or inhaled pollutants [250,251,252,253,288].

AhR deficiency or reduction entails an increase in susceptibility to cigarette smoke-induced damage and promotes the development of COPD and emphysema in humans due to the inability to regulate a complex network of interconnected pathogenic mechanisms that lead to irreversible lung injury. Clinical studies of COPD patients have shown a significant reduction in pulmonary and systemic AhR gene expression in COPD patients compared to healthy individuals. Moreover, systemic AhR mRNA levels are positively correlated with lung function; patients with lower expression levels often have worse respiratory outcomes [254,321].

Thus, AhR ligand-independent functions critically dictate the pathogenesis, progression, and suppression of COPD [1,131,316,321]. Baseline AHR expression acts as a primary genetic checkpoint protecting the lungs. When AhR expression drops, multiple interdependent biological pathways fail simultaneously, driving alveolar destruction [1,131,321,322]. COPD and emphysema are largely caused by exposure to cigarette smoke, which causes severe alveolar damage. In the absence of an active chemical ligand, the basic expression of AhR acts as the main post-transcriptional buffer [316,321]. AhR protects the pulmonary microenvironment from degeneration via several key pathways: microRNA modulation, transcription factor cascades, protease/anti-protease imbalance, and apoptosis suppression. Without ligand binding, AhR suppresses pathological microRNAs like miR-96, which otherwise blocks protective tissue responses [323].

In the absence of basal AHR expression, uncontrolled miR-96 suppresses critical survival factors such as FOXO3a and the antioxidant gene Srxn1. This ligand-independent loss triggers a key hallmark of emphysema and COPD—airway dilation—causing increased oxidative stress, decreased SOD2 activity, and increased apoptosis [321,323,324].

Basal AhR expression maintains the balance between matrix metalloproteinase-9 (MMP-9) and Tissue Inhibitor of Metalloproteinases-1 (TIMP-1), preventing structural degradation [321,325,326]. AhR presence represses cleaved caspase-3 expression, preventing premature structural lung cell death and emphysema development [316,321,327,328]. Furthermore, a critical factor regulating cell growth and apoptosis, as well as promoting inflammation and cell death in the lungs, is KLF6, which interacts with AhR through a non-canonical signaling mechanism [64,83,329]. Finally, AhR promotes inflammation resolution through efferocytosis, and the absence of AhR leads to the accumulation of apoptotic neutrophils, which exacerbates chronic inflammation [330]. This often occurs via a non-genomic pathway involving IL-10/JAK/STAT3 signaling [330,331].

Considering that AhR suppresses pulmonary neutrophilia and inflammation, AhR deficiency enhances cigarette smoke-induced RelB degradation. Unliganded AHR is required to maintain the baseline structural stability of key anti-inflammatory proteins [251,324]. In resting lung cells, cytosolic AhR physically associates with RelB, preventing its premature degradation [324]. When AhR expression is lowered (as observed systemically in human COPD patients, RelB is rapidly degraded. This ligand-independent degradation unleashes an overproduction of the intercellular adhesion protein 1 (ICAM-1), resulting in chronic, damaging neutrophilia and worsening tissue damage in COPD [321,324]. Increased expression of ICAM1 and increased neutrophil infiltration, as well as elevated levels of the pro-inflammatory enzyme COX-2, ultimately lead to significant enhancement of lung inflammation [248,250,251,252,253,260,314,332,333]. Thus, AhR may attenuate cigarette smoke-induced neutrophilization via non-canonical interaction with RelB and may also regulate COX-2 overexpression.

AhR deficiency also leads to decreased levels of the antioxidant enzymes SOD2 and NQO1, which increases ROS production during cigarette smoke exposure [316,334,335,336]. There is also a genetic predisposition to a higher risk of developing severe COPD associated with the genetic polymorphism rs2066853 (A allele and GA genotype) in the AhR gene, resulting in lower AhR expression [322].

The translation of acute AhR modulation results to chronic COPD progression is limited by temporal paradoxes, the dual nature of AhR ligands, and dynamic receptor desensitization over time. While transient AhR activation exerts a short-term protective effect, chronic activation can accelerate irreversible tissue damage and the development of emphysema [337]. In the short term or at low doses, acute AhR activation by natural ligands (or therapeutic compounds) triggers adaptive, protective responses. It induces xenobiotic metabolism, controls cellular antioxidants, and temporarily dampens neutrophilic inflammation [252,321].

Conversely, chronic, decades-long AhR hyperactivation by agonists (like the polycyclic aromatic hydrocarbons in cigarette smoke) transitions from physiological protection to profound toxicity. Sustained AhR activity drives mitochondrial dysfunction, oxidative stress, tissue remodeling, mucus hypersecretion, and structural skeletal muscle atrophy in patients continuously exposed to tobacco smoke [1,251,323,337,338,339]. In acute AhR modulation, direct reactions of signaling pathways are observed. However, long-term exposure induces negative feedback loops such as the upregulation of the AHRR or TIPARP which attenuate the intended downstream effects and alter the AhR homeostatic functions over time [232,324]. The physiological response to AhR ligands varies drastically depending on the cell type and the micromilieu. Acute, localized responses cannot account for the systemic, multi-organ pathophysiology seen in COPD over decades [324,338,340].

Thus, AhR controls immune, inflammatory, homeostatic, and toxic cellular responses to endogenous and exogenous stimuli. On the one hand, AhR may play a role in the pathogenesis of COPD, when COPD is caused by cigarette smoke and industrial pollutants due to impaired immune homeostasis in the lungs. On the other hand, AhR activation may attenuate inflammation caused by the disruption of immune homeostasis.

### 8.2. Asthma

Many immune cell types, including eosinophils, T cells, B cells, and epithelial cells, which are involved in the asthmatic response, express AhR, and its coactivation by ligands can regulate the outcome of the response.

Regulation of T cell differentiation is important in the control of airway inflammation in asthma. CD4^+^ T cell subpopulations (Th9, Th17, and Th22) producing IL-9, IL-17, and IL-22 are implicated in the pathogenesis of asthma, while IL-9, IL-17, and IL-22 are considered important mediators of asthma pathogenesis [341,342]. Furthermore, asthma is associated with the differentiation and activation of Th2 cells and their cytokines (IL-4, IL-13, and IL-5) [343], and Th17 cells [343,344] with subsequent eosinophilic and neutrophilic infiltration of the airways, respectively. Neutrophilic airway inflammation occurs in more severe asthma [345,346,347,348].

An important negative regulator of the allergic reaction is Tregs, the functional impairment of which enhances the activation of effector T lymphocytes through the production of pro-inflammatory DCs [349,350], which entails the generation of various Th2/Th17 subpopulations [351].

A clinical study of patients with allergic asthma revealed an increase in the expression of IL-22 and IL-17, as well as the AhR target gene CYP1A1, in peripheral mononuclear cells compared to healthy controls. Diesel exhaust stimulation was shown to increase AhR-dependent IL-22 production by CD4^+^ cells with a simultaneous reduction in IL-17 [352].

A clinical study of asthma patients identified a key link between AhR and asthma severity. Patients with severe allergic asthma often exhibit a high level of AhR nuclear translocation in bronchial epithelial cells, which correlates with more severe bronchial inflammation [341]. This study demonstrated that AhR activation by diesel exhaust particulates mediated increased expression of IL-33, IL-25, and epithelial thymic stromal lymphopoietin (TSLP) with Th2 activation, which potentially linked environmental pollution and severe allergic asthma. In this regard, bronchial biopsies from 28 patients with severe allergic asthma and 6 patients with mild asthma from highly polluted areas were analyzed for AhR nuclear translocation (NT), cytokine expression, and gene activation. During the cultivation of primary epithelial cells stimulated with diesel exhaust particles, increased expression of IL-33, IL-25, and TSLP was detected. These effects were abrogated by suppressing AhR expression using siRNA. In addition, increased AhR/ARNT binding to the IL-33, IL-25, and TSLP promoters was detected. Patients with severe allergic asthma and high AhR/ARNT levels showed higher expression of the genes and proteins of IL-33, IL-25, and TSLP in the bronchi [341].

A study of lung fibroblasts from patients with asthma demonstrated a significant reduction in AhR expression compared to healthy individuals, which directly promoted the structural changes inherent in chronic asthma [353,354]. AhR deficiency or low levels typically correlate with increased airway inflammation, which worsens asthma symptoms and leads to increased collagen deposition, bronchial wall remodeling, and airway hyperresponsiveness. Myofibroblast accumulation is a key feature of airway remodeling, leading to subepithelial fibrosis, which significantly contributes to irreversible airway obstruction in asthma [353].

There is also a genetic predisposition to a higher risk of developing bronchial asthma in children, associated with a genetic polymorphism of the AhR target gene CYP1A1 (Valine allele) [355].

### 8.3. Pulmonary Fibrosis

The role of AhR in pulmonary fibrotic diseases depends on the context and cell type. Chronic smoking and environmental pollutants are known risk factors for pulmonary fibrotic diseases. Exposure to these factors activates AhR in lung epithelial cells (Clara cells) and immune cells (Th17), leading to exacerbation of Th17-mediated lung injury and fibrosis [302,356]. However, in patients with idiopathic pulmonary fibrosis, clinical studies show a significantly reduced AhR expression compared to healthy individuals [357]. These data were obtained using a human interstitial lung fibrosis tissue microarray—a microarray with 24 tissue samples.

Studies of AhR in pulmonary fibrosis indicate that AhR signaling may influence the severity of fibrosis, particularly through immune regulation. Currently, the most compelling evidence for AhR’s role in pulmonary fibrotic diseases comes from animal models rather than human clinical trials. In an animal model, knockout of the AhR receptor has been shown to result in more severe pulmonary fibrosis, while its activation with specific ligands can reduce scarring. This suggests that AhR exerts a protective effect against pulmonary fibrosis [357].

Furthermore, AhR deficiency triggers the pro-fibrotic insulin-like growth factor 1 (IGF1R) signaling cascade in bleomycin-induced pulmonary fibrosis and causes alterations in epithelial–mesenchymal transition and cellular permeability. The IGF1R level is often elevated in patients with idiopathic pulmonary fibrosis and promotes fibrosis, while AhR levels are decreased. It is assumed that restoring AhR activity may suppress the pro-fibrotic signaling induced by IGF1R [357].

The pro-inflammatory cytokine IL-17 plays a pathogenic role in chronic lung diseases, including idiopathic pulmonary fibrosis. It affects epithelial cells and fibroblasts, promoting epithelial–mesenchymal transition, myofibroblast differentiation, and increased collagen production. IL-17 attracts inflammatory neutrophils to the site of injury, accelerating structural lung damage. In patients with idiopathic pulmonary fibrosis, IL-17A levels are significantly elevated in the serum [358].

Specific activation of AhR by certain ligands has been shown to attenuate pulmonary fibrosis by enhancing the response of Tregs and suppressing inflammatory T cell subpopulations which produce IL-17A. One key study revealed that AhR activation with the FICZ ligand reduced fibrosis and improved survival in a bleomycin-induced mouse model, which was associated with an increase in Tregs and a decrease in inflammatory T cell subpopulations. In this study, AhR stimulation reduced Ashcroft fibrosis scores and lung collagen levels, simultaneously increasing the number of CD4^+^FOXP3^+^ regulatory T cells [359]. The same study reported lower numbers of pro-inflammatory T cells CD4^+^IFNγ^+^ and γδ^+^IL-17A^+^ in the lungs after AhR activation.

Experimental data suggest several molecular mechanisms for AhR’s role as a protective factor in regulating pulmonary fibrosis. First, it increases the level of Tregs and suppresses inflammatory cell subpopulations, as well as influences TGF-β1, EMT signaling, and fibroblast activity [224]. Second, AhR activation reduces the TGF-β1-induced transition of fibroblasts to myofibroblasts, which are the main cells responsible for the formation of excess scar tissue, and promotes lung repair by enhancing alveolar cell regeneration [235,359].

Thus, AhR can exacerbate pulmonary fibrosis in response to environmental toxins or potentially attenuate it through therapeutic intervention with ligands. AhR in pulmonary fibrosis can be considered a pathway, which under activation can inhibit inflammation and scarring, but the ultimate role depends on the model, ligands, and cellular context.

### 8.4. Interstitial Lung Disease (ILD)

ILD is a group of over 200 chronic lung diseases that cause progressive scarring (fibrosis) or inflammation of the tissue between the alveoli. Common causes include occupational/environmental factors (asbestos, silica dust, coal dust, or mold), autoimmune diseases (e.g., rheumatoid arthritis), side effects of certain medications (chemotherapy, heart medications) or radiation therapy, and smoking, although many cases are idiopathic. Fibrosis (the replacement of normal tissue with scar tissue) is a common outcome of ILD [360,361,362].

AhR acts as a key mediator in various forms of ILD [363,364]. AhR plays a crucial role in the regulation of pulmonary inflammation and fibrosis in patients with ILD [359,365]. In patients with systemic sclerosis-associated ILD, AhR mRNA expression levels in peripheral blood mononuclear cells are significantly higher compared to those without ILD [366]. In addition to increased AhR expression in interstitial lung diseases, AhR mRNA expression levels negatively correlate with lung function [366].

AhR links the influence of environmental factors to biological mechanisms of diseases such as rheumatoid arthritis-associated interstitial lung disease (RA-ILD) [362,365,367,368].

AhR activation leads to immune dysfunction through affecting T cell differentiation, which promotes the inflammatory environment and autoimmune processes inherent in rheumatoid arthritis. Studies show that AhR expression and activation (e.g., CYP1A1 expression) are higher in peripheral blood mononuclear cells in patients with rheumatoid arthritis compared to healthy controls [369]. Patients with higher serum AhR activity are more susceptible to severe clinical consequences [361,370]. RA-ILD shares common inflammatory pathways (TGF-β//SMAD, JAK/STAT, PI3K–Akt, Wnt/β-catenin) with other fibrotic lung diseases, which may be influenced by AhR activation [361,365].

Silicosis is one of the ILDs that causes progressive fibrosis or inflammation of the tissues between the alveoli. AhR links exposure to silica dust to the biological mechanisms of this disease. In patients with silicosis, AhR acts as a key regulator of pulmonary inflammation and fibrosis, as well as the body’s immune response to silica particles, which leads to disease progression [371]. AhR involvement in the development of silicosis is characterized by features such as immune regulation and the balance between Th17 and Tregs.

Activation of the AhR has been shown to attenuate silica-induced inflammation by reducing the recruitment of neutrophils and inflammatory cells to the lungs [372,373]. Studies suggest that regulation of the AhR pathway is critical for controlling Th17 cells, which promote inflammation and fibrosis, and Tregs, which help suppress excessive immune responses [374,375,376].

Patients with silicosis often exhibit better lung function at high serum mannan-binding lectin (MBL) levels [371]. MBL has been shown to directly bind to AhR, limiting its nuclear translocation, and to constrain AhR/STAT3 pathway activation by suppressing STAT3 phosphorylation that, in turn, reduces Th17 cell-mediated lung inflammation and improves lung function [371].

In conclusion, it is important to emphasize that in biomedical research, there are several terms that describe the hierarchy of evidence, which denotes the transition from simple correlation to specific, targeted causation. The terms “associated,” “proven,” “mechanistically driven,” and “therapeutically validated” reflect different stages of understanding of the role of a gene, protein, or signaling pathway in disease development—from simple observations to a solid evidence base. “Associated” refers to a statistical or observational association. This is a starting point that requires further verification. “Proven” implies a higher level of certainty. The association goes beyond simple correlation and demonstrates causality in different contexts. “Mechanistically implicated” means that specific molecular or cellular mechanisms are identified. “Therapeutically validated” means that not only has the role been proven and the mechanism understood, but also that the targeted intervention actually produces a clinical effect and has an acceptable safety profile. The data presented in this review reflect the understanding of the role of AhR in inflammatory lung diseases as a causative factor, an inflammation modifier, or a biomarker at stages that are “associated,” but in specific cases “proven” and “mechanistically determined”.

Table 2 summarizes data indicating the dual role of AhR in the immune and inflammatory systems depending on the lung cells involved and the AhR ligands, based on data from experimental and clinical studies.

## 9. Conclusions

In inflammatory non-infectious lung diseases, AhR functions as a critical environmental sensor that orchestrates immune and inflammatory responses. AhR plays a context-dependent dual role, acting either as a pathogenic and pro-inflammatory trigger or as a protective and anti-inflammatory regulator.

The dual functionality of AhR is governed by the five pillars—ligand class, receptor affinity, cell type, tissue microenvironment and disease stage [130,377,378,379,380]. The mechanism of AhR dualism is realized primarily due to the ability of AhR to recognize a wide range of ligands, ranging from environmental toxins to beneficial nutrients and microbial metabolites, as well as the duration of AhR activation by ligands and the specific cell type expressing AhR [378,381].

The functional consequences of AhR activation depend entirely on the type of AhR ligand, dictating whether the resulting response is protective and anti-inflammatory or promotes tissue damage and chronic inflammation. The diversity of AhR ligands drives distinct functional consequences in the lung [1,164,382,383,384]. Environmental pollutants and xenobiotics cause high-affinity, prolonged activation of AhR, resulting in the excessive transcription of cytochrome P450 enzymes (CYP1A1, CYP1B1). This excessive metabolism generates massive amounts of ROS and oxidative stress [322].

This pathway promotes airway remodeling, mucus hypersecretion, and the release of inflammatory alarmins from epithelial cells. Ultimately, this exacerbates conditions like asthma and COPD [164]. Endogenous and dietary molecules bind to AhR with lower, transient affinity. They promote regulatory Treg differentiation, regulate Th17/Th22 subsets, and promote anti-inflammatory M2 macrophage polarization [130,233]. These ligands generally protect the lung barrier. They suppress excessive pulmonary neutrophilia, resolve inflammation following injury, and combat bacterial infections by driving coordinated immunity [1,251,316,385].

Thus, high-affinity persistent ligands, e.g., cigarette smoke, urban pollutants, and TCDD, often exert pathogenic effects, while rapidly metabolized nontoxic ligands, e.g., dietary indoles, microbial metabolites, and FICZ, can exert protective effects. Transient (short-term) AhR activation can promote tissue repair and inflammation resolution, whereas persistent, chronic activation promotes tissue damage [131,156,232,379,382,386]. AhR signaling can be canonical or non-canonical depending on the cellular context. In regulatory Tregs, AhR activation suppresses inflammation, while in certain other immune or tumor cells, it can promote cellular exhaustion, migration, or proliferation [131,232,379,382].

Activated AhR can guide T cell differentiation, causing different immunological changes. AhR promotes Th17/Th2 (key mediators include IL-17, ROS, and Th2 cytokines), stimulating inflammation and exerting a pathogenic effect. A shift in the immune balance toward Th17 cells leads to high levels of IL-17, which induces severe neutrophilic inflammation and tissue damage. Th2 cell differentiation leads to increased production of IL-4 and IL-5, which exacerbate asthma symptoms. Activated AhR can exert a protective effect by promoting Tregs (key mediators IL-10 and IL-22) and suppressing inflammation. The common denominator contribution is the resolution of inflammation without scarring, while suppressing excessive effector responses and supporting tissue repair via IL-22 [1,387,388,389].

In lung dendritic cells, activated AhR reduces their ability to stimulate effector T cells, alternatively enhancing Treg or Th17 differentiation, depending on the context. AhR modulates T cell differentiation, ultimately promoting immune tolerance, reducing allergen-specific Th2 responses, and redirecting the immune response from an allergic to a protective one. In mast cells, activated AhR can enhance IgE-dependent mast cell degranulation, increasing the release of histamine and pro-inflammatory cytokines. Furthermore, in pulmonary smooth muscle, AhR regulates proliferation and contractility, which affects bronchial constriction and chronic wall thickening. Thus, pulmonary dendritic cells prevent inappropriate activation of the adaptive immune system, preventing naive T cells from producing aggressive responses to harmless antigens [390]. In lung epithelial cells, AhR operates as a regulator of mucosal barrier function and controls mucin production. The functional consequences of AhR activation are that by clearing pathogens and maintaining integrity, it prevents DAMPs from leaking into deeper tissues and triggering systemic immune reactions [232,391]. In epithelial cells, AhR reduces inflammation by suppressing pro-inflammatory responses (TNF-α, IL-6, CXCL2). In lung macrophages and Clara cells, AhR promotes the production of anti-inflammatory mediators and regulates Notch signaling, which is essential for tissue repair. AhR attenuates neutrophilia and early inflammation induced by cigarette smoke via inhibiting pro-inflammatory NF-κB/RelB signaling and reducing COX-2 expression.

The collective AhR signaling across lung epithelial cells, dendritic cells and T cells establishes a master regulatory network [125]. Rather than independent responses, these cell types coordinate to create a “common denominator” effect: robust mucosal tolerance, barrier integrity, and the suppression of overzealous inflammation [203].

In the tissue microenvironment, the local environment of cytokines, growth factors, and metabolites significantly influences the interaction of the AhR complex with coactivators. AhR interprets these local data to adapt gene expression to the immediate physiological or inflammatory context [378,381,382]. The effect of AhR signaling shifts as pathologies progress. The stage-specific, dynamic shifting of AhR signaling is a critical determinant in the progression of lung inflammatory diseases, evolving from being protective in early stages to pathological in advanced or chronic phases [392].

AhR acts as a “common denominator” across heterogeneous lung diseases such as asthma, COPD, fibrosis, and ILD. Though these conditions have diverse triggers, they all share dysregulated immune–inflammatory pathways and tissue remodeling.

In asthma and allergic reactions, targeting AhR in airway smooth muscle may help modulate transcription factors, which operate asthma-associated inflammation. Using specific AhR ligands to restore immune tolerance in allergic diseases could potentially improve the efficacy of allergen-specific immunotherapy [150,271]. In COPD and smoking-induced damage, AhR agonists may help protect against parenchymal destruction by suppressing pulmonary neutrophilia and oxidative stress caused by chronic exposure to tobacco smoke. In interstitial lung diseases and associated fibrotic conditions caused by chronic smoking, AhR activation may also help protect the lungs from damage, as AhR activation suppresses pro-inflammatory T cell subpopulations, creating an environment conducive to tissue regeneration rather than scar formation. In idiopathic pulmonary fibrosis, AhR agonists may help slow down the progression of fibrosis by suppressing EMT via targets such as the TGF-β/Smad pathway, which is a major factor inducing fibrosis [235,393]. In silicosis, AhR activation may help reduce inflammation by suppressing the production of the inflammatory cytokine IL-1β [372,373], and AhR inhibition may help halt or slow the irreversible fibrosis, which is inherent in advanced silicosis, by modulating the AhR/STAT3 pathway in which STAT3 is a fibrosis-promoting factor [371].

In the context of inflammatory lung diseases, AhR is primarily proposed as a vital modifier of inflammation, a protective gatekeeper, and a promising therapeutic node. It is less frequently characterized as a “central causal driver,” and its direct utility as a standalone biomarker is still emerging compared to its role in targeted treatments.

The primary role of AhR is as an inflammation modifier. Significant studies emphasize that AhR acts as a key negative regulator and mitigator of lung inflammation. In response to irritants or pathogens, AhR suppresses pro-inflammatory cascades (for example, by limiting activation of the NF-kB pathway). AhR also maintains respiratory homeostasis by balancing immune cells. AhR helps maintain and restore the lung’s physical barrier, preventing pathogens and pollutants from penetrating into surrounding tissues.

AhR as a central causal factor plays a secondary context-dependent role. AhR is only rarely considered as a direct, primary “driver” of pathogenesis, usually in the context of extreme exogenous toxicity. However, in most chronic inflammatory lung diseases, the function of AhR is protective and modulating, rather than causative and destructive.

AhR plays an emerging role as a biomarker. AhR is increasingly being studied as an indicator of disease susceptibility and severity. For example, studies have shown that patients with COPD have reduced expression of pulmonary and systemic AhR. Changes in AhR pathway gene expression (such as CYP1A1 levels) may also serve as markers for monitoring exposure to environmental pollutants and lung inflammation.

The therapeutic node is a major role of AhR. The widespread expression of AhR in various lung cells and its role in regulating the inflammatory effects caused by environmental pollutants and allergens make it an attractive therapeutic target, which can be effectively regulated in a cell-type-specific manner. The AhR signaling pathway offers numerous opportunities for the development of new treatments for inflammatory lung diseases.

Targeted AhR agonists (e.g., dietary indoles, gut microbiome metabolites, and specific pharmaceutical compounds) are being actively explored. They are used to enhance the receptor’s anti-inflammatory and barrier-protective functions in diseases such as asthma and cystic fibrosis.

Selective AhR modulators (SAhRMs), which exhibit agonist and antagonist activity in a tissue- or cell-specific manner, are of therapeutic interest. SAhRMs induce beneficial anti-inflammatory or protective responses without the extreme toxicity associated with classical ligands such as TCDD [98,394,395].

Currently, several ligands have been identified, which exhibit tissue-specific or selective activity in the respiratory system [98]. Endogenous compounds such as kynurenine, FICZ, and ITE can naturally activate AhR and regulate the barrier integrity and immune homeostasis in the lungs [130,224,396,397]. Preclinical studies have shown that kynurenine can significantly reduce lung injury and reverse advanced fibrosis [398], and FICZ is able to reduce pulmonary fibrosis and improve survival via regulating the overexpression of pro-inflammatory cytokines [359,361,399].

Naturally occurring AhR ligands such as resveratrol and quercetin have been shown to provide lung protection against oxidative stress and environmental toxins, while flavipin and punicalagin, derived from pomegranate, have shown promising results in regulating macrophage-mediated inflammation [233]. Resveratrol is considered a potential therapeutic agent for diseases caused by excessive activation of immune and inflammatory processes, particularly severe asthma and air pollution-induced respiratory damage [400,401]. Some natural compounds, e.g., curcumin and flavonoids, belonging to AhR ligands are considered anti-asthmatic agents capable of modulating the Th2 response [402].

Research has identified several repurposed AhR agonists with potential for treating inflammatory diseases. These include the pharmaceutical SAhRMs, such as tranilast, omeprazole, and WAY-169916, which can be used as anti-inflammatory agents and potential treatments for COPD and asthma. The anti-allergy drug Tranilast, which acts as a selective AhR agonist, is used to reduce allergic inflammation in the lungs [403].

The proton pump inhibitor omeprazole, which is an atypical AhR ligand, can protect the lungs from oxidative damage [404]. The selective estrogen receptor modulator WAY-169916, which also acts as an SAhRM, can exert anti-inflammatory effects and suppress the expression of genes for the acute-phase inflammatory response [405].

A recent high-throughput screening study identified febuxostat, nitazoxanide, and teriflunomide among existing drugs, which have significant AhR agonist activity [406].

Thus, AhR is a therapeutic target worthy of further investigation. Specific AhR modulators may help reduce inflammation and protect lung tissue in the future. However, a significant challenge in developing SAhRMs is related to the fact that their selectivity as agonists or antagonists cannot be easily predicted, and large-scale studies are needed to identify the optimal ligand for specific clinical applications in inflammatory lung diseases.

## Figures and Tables

**Table 1:** AhR in the regulation of immune cell development and functions.

Activated AhR	Immunity	Innate	Cell type	Development/Function	Effects
			Mac	Differentiation Pro-inflammatory Anti-inflammatory	↑ M2, IL-10 ↑ M1, IL-1β, TNF-α
			DC	Differentiation Activation	↑ Monocytes → DC ↑ CD80, CD86, MHC II
			MDSC	Differentiation Immunosuppressive	↑ PMN-MDSCs ↑ CXCR2 ↑ MDSC mobilization
			NK	Cytotoxicity	Interferon-γ production ↑ IL12, IL10 ↓ CD56
			ILC	Development Survival Phenotype Change	↑ IL-C2 → IL-C3 ↑ ROR γt ↑ IL-22
		Adaptive	CD8^+^	Differentiation Depletion of Proliferation	↑ CD8^+^ T cell depletion ↑ Trm
			Native CD4^+^ T	Differentiation	↓ Th2, IL4, IL5 ↑↓ Th17, IL-22, IL17, ↑↓ Treg, CD30, IL10, IL21
			Treg	Differentiation Anti-inflammatory	↑ Foxp3 ↓ Pro-inflammatory cytokines
			Th17	Differentiation Pro-inflammatory Anti-inflammatory	↑ IL-22 ↑ IL-17A
			Th1	Differentiation	↑ IL10, CCR9
			Th2	Differentiation	↑ IL-4, IL-5, IL-13
			B	Differentiation Immunosuppressive Proliferation	↑ Breg ↑ IL-10 ↓ Plasma, early B and pre-B cells

Abbreviations: ↑, increase; ↓, decrease.

**Table 2:** AhR’s dual role in the immune and inflammatory systems of the lung.

AhR Agonists/ AhR Antagonists/ AhR Knockdown	Rodent Models/ Lung Cells/ Cell Lines/ Humans/Patients	Effects	Mechanism	Refs.
TCDD	Rodents with asthma	Induction of a distinct pro-inflammatory state	Reduction in the production of Th2 cytokines such as IL-4, IL-5, and IL-15.	[245,247]
TCDD	Mouse lungs	Increased inflammation	Increased the expression of TNF-α, IL-1β, MCP-1, mucin 5AC, and MMP-12 and MMP-13 through AhR activation via XREs in the promoter/enhancer regions of these genes.	[245]
TCDD or BaP	Lung epithelial cells	Increased inflammation	Increased expression of IL-8 and MUC5AC via the AhR/EGFR/MAPK pathways and antagonism of IFN and RA signaling have been demonstrated.	[312,313]
TCDD	*AhR*^−/−^ mice, asthma model induced by cockroach allergen	Suppressed inflammation	Cockroach allergens induce activation of AhR signaling in bone marrow-derived mesenchymal stem cells. AhR regulates MSC migration and protects lungs from cockroach allergen-induced inflammation though MSCs.	[258]
TCDD cockroach allergen	Mesenchymal stem cells (MSCs), *AhR*^−/−^ mice	Inhibited lung inflammation	Lung protection from allergen-induced inflammation by modulating MSC recruitment and its immune-suppressive activity.	[258]
TCDD or (1’H-indole-3’-carbonyl)-thiazole-4-carboxylic acid methyl ester	Bronchial epithelial cells (Beas-2B or HBE135-E6E7), asthmatic patients	AhR pathway is target for treating asthma	Increased MMP-1 expression in bronchial epithelial cells by AhR agonists. The upregulated MMP-1 further activated MMP-2 and MMP-9. Asthmatic patients have higher serum MMP-1 levels. AhR pathway involves CaMKII, MAPKs, and AP-1 elements.	[245,314,315,316,317]
TCDD, CH122319, AhR knockdown	Human lung fibroblasts from asthmatic and healthy individuals, human lung fibroblasts (WI-38) treated with cockroach extract (CRE)	Possible synergistic effect between exposure to allergens and environmental chemicals in the development of allergic diseases	Role of AhR in modulation of cockroach allergen-induced immune responses through controlling the active TGFβ1 release.	[353,354]
AhR knockdown	Lungs of idiopathic pulmonary fibrosis patients, bleomycin (BLM)-induced fibrosis in wild-type and *AhR*^−/−^ mice, Beas2B cells	Protective effect of AhR against pulmonary fibrosis	Knockout of AhR aggravated lung fibrosis but the IGF1R inhibitor and AhR agonist significantly attenuated such effects and inhibited TGF-β1-induced EMT in Beas2B cells. Both TGF-β1 and BLM markedly suppressed AhR expression through endoplasmic reticulum stress and, consequently, IGF1R activation. AhR and IGF1R play opposite roles via the TGF-β/Smad/STAT signaling cascade.	[357]
PAH mixture, Benzo(a)pyrene, FICZ	C57BL/6 mice, primary mouse alveolar epithelial cells (AECs), mouse MLE-15 epithelial cell line	Preservation of alveolar integrity by modulating inflammatory cytokine expression	Role of alveolar epithelial AHR activity in modulating inflammatory responses and maintaining alveolar integrity during lung injury and repair. AhR activation in AECs preserves alveolar integrity by modulating inflammatory cytokine expression while enhancing barrier function and limiting stress-induced expression of mesenchymal genes.	[224]
FICZ	Murine and human mast cells	Modulating allergic responses and contributing to the generation of inflammation-related diseases	Modulates acute and late mast cell responses.	[277,281]
FICZ	BALB/c mice	Anti-asthmatic effects on ovalbumin-induced allergic asthma	Suppresses pulmonary eosinophilia and Th2-type cytokine production, regulating GATA-3 expression and SATA6 activation. AhR is functional under the Th2 differentiation condition, inducing Cyp1A1 expression.	[277,278,285]
FICZ	BLM-induced fibrosis model	Reduction in fibrosis	AhR stimulation attenuated lung fibrosis by increasing Tregs and suppressing inflammatory T cells.	[359]
Highly polluted areas, diesel exhaust particles (DEPs)	Bronchial biopsies for allergic severe asthma and mild asthma subjects, primary epithelial cells	AhR activation by DEP mediates upregulation of IL-33, IL-25, and TSLP with Th2 activation, potentially linking environmental pollution and allergic severe asthma	Allergic severe asthma with high AhR nuclear translocation had higher bronchial gene and protein expression of IL-33, IL-25, and TSLP. Upregulation of IL-33, IL-25, and TSLP in primary bronchial epithelial cells exposed to DEP.	[341,342]
DEP-PAH, Benzo[a]pyrene	Peripheral blood mononuclear cells from allergic asthmatic subjects exhibiting an increased Th17/Th22-type profile	Increased IL22 production in both healthy and asthmatic subjects under some of the PAH action	DEP-PAH and B[a]P may contribute to increased IL22 production in both healthy and asthmatic subjects through mechanisms involving both AhR-dependent and -independent pathways. IL-17 production was inversely downregulated in asthmatic as well as healthy subjects.	[352]
Benzo(a)pyrene co-exposure with allergen Der f 1	C57BL/6 mice	Facilitated Der f 1-induced asthma	BaP facilitates Der f 1-induced epithelial cytokine release through the AhR-ROS axis.	[275,286]
Environmental pollutants (vehicular exhaust particles, Indeno [1,2,3-cd]pyrene, ultrafine particles)	In vitro culture assays, in vivo murine models, asthmatic subjects	Allergic airway inflammation	Activated AhR causes gene transcription and ultimately oxidative stress, inflammation and cellular change.	[268,269,270,271]
Standard Reference Material (SRM) 1649b urban dust, freshly collected samples of diesel exhaust and cigarette smoke	*AhR*^−/−^ and *DREC*^−/−^, wild-type C57BL/mice	Airway pathology	Increased T cell differentiation (increased generation of IL-17A) and increased populations of IL-17-producing cells in wild-type but not *AhR*^−/−^ mice. PM directly acts on the AHR in T cells, leading to enhanced Th17 differentiation.	[274]
PM2.5 or IP	C57BL/6 mice	Aggravated cockroach extract (CRE)-induced asthma	AhR-HIF 1α pathway mediates pulmonary responses through the ability to disturb the balance of Th17/Treg cells.	[279]
PM2.5 or IP	PBMCs of healthy human volunteers	Posed a significant risk for asthma	AhR affects T_H_17/Treg cell imbalance.	[279]
PM 2.5 co-exposure with house dust mite (HDM)	BALB/c mice	Enhanced the pulmonary allergic immune response of HDM-induced asthma	PM 2.5 enhances the pulmonary allergic immune response to HDM by augmenting Th2- and Th17-immune responses.	[275,276]
Indeno [1,2,3-cd]pyrene (IP)	C57BL/6 mice, DC-*AhR*^−/−^ mice	Exacerbated allergic inflammation (ovalbumin- sensitized allergic asthma)	IP affects DC functions through modulating AhR activity, leading to enhanced allergic inflammation. IP significantly enhances antigen-induced allergic inflammation (increased airway eosinophilia, Th2 cytokines IL-4 and IL-5, and specific IgE level), which was absent in DC-*AhR*^−/−^ mice.	[269]
Omeprazole	Mice with hyperoxic lung injury	Attenuated lung inflammation	Reduced neutrophil infiltration and MCP-1 expression.	[246]
Tobacco smoke or LPS	Mouse *AhR*^−/−^	Increased lung inflammation	RelB is prematurely degraded, resulting in heightened inflammatory responses to multiple pro-inflammatory stimuli.	[250,260]
Cigarette smoke, transient transfection of an AhR expression plasmid into AhR^−/−^ fibroblasts	Culture of primary fibroblasts of *AhR*^−/−^ C57BL/6 mice	AhR^−/−^ mice produce a heightened inflammatory response	Significantly attenuated the smoke-induced COX-2 and PG by maintaining RelB expression.	[248]
Cigarette smoke extract, AhR antagonists	Human lung fibroblasts	Lung inflammation	Inhibited AhR nuclear translocation and COX-2, mPGES-1, and prostaglandin E production.	[249]
Cigarette smoke for 2 weeks or 4 months	Mouse *AhR*^−/−^	Increased lung inflammation	Neutrophilia.	[250]
Cigarette smoke acute and subchronic exposure	Mouse *AhR*^−/−^	Increased lung inflammation	Suppressed pulmonary neutrophilia.	[250,251,252]
Cigarette smoke	Primary lung fibroblasts generated from *AhR*^−/−^ C57BL/6 mice	Reduced pulmonary cell survival	Significantly decreased MnSOD and CuZn-SOD expression (that protect against oxidative stress).	[254]
Cigarette smoke LPS	C57BL/6 mice	Exacerbated the inflammatory response and induced diffuse structural changes in the alveolar parenchyma	Increased the numbers of neutrophils, macrophages, and CD4^+^ and CD8^+^ T cells.	[255]
Cigarette smoke	Primary lung fibroblasts were generated from *AhR*^+/+^, AhR heterozygous (*AhR*^+/−^) and *AhR*^−/−^ C57BL/6 mice; primary human lung fibroblasts	AhR plays an important role in suppressing the expression of inflammatory proteins	Activated AhR destabilizes *COX-2* mRNA by retaining HuR within the nucleus, suppressing an exaggerated increase in COX-2 protein expression. The AhR retention of nuclear human antigen R (HuR) and subsequent suppression of COX-2 protein does not involve classic AhR:DNA binding.	[248,250,251,252,253,260,314,332,333]
Cigarette smoke extract (CSE)	Primary lung fibroblasts derived from *AhR*(^+/+^) and *AhR*(^−/−^) mice, lung fibroblasts derived from COPD subjects	Low AhR expression may facilitate the development or progression of COPD	AhR prevents smoke-induced apoptosis by regulating oxidative stress. CSE induced a significant increase in the mRNA expression of key antioxidant genes, including *Nqo1* and *Srxn1*, predominantly in *AhR*(^+/+^) fibroblasts, with significantly less induction in *AhR*(^−/−^) cells. COPD-derived fibroblasts were less robust in their induction of both *Nqo1* and *Srxn1* mRNA after exposure to CSE.	[316,334,335,336]
Cigarette smoke	Bone-marrow-derived macrophage (BMDM) from *AhR*^−/−^ mice	AhR protects against the damaging effects of cigarette smoke by attenuating pulmonary neutrophilia	AhR promotes the resolution of cigarette smoke-induced inflammation in mice via enhanced efferocytosis. The ability of macrophages to engulf apoptotic neutrophils in the lungs is due to a non-genomic AhR pathway that involves signaling through the IL-10/JAK/STAT pathway. Nontoxic endogenous AhR ligand FICZ promotes macrophage uptake of neutrophils.	[330]
Cigarette smoke	*AhR*^−/−^ mice (strain B6.129-AhR^tm1Bra^), smokers with COPD	Protection in the pathogenesis of smoke-induced emphysema/COPD	AhR-deficient mice chronically exposed to cigarette smoke develop airspace enlargement concomitant with a decline in lung function, and also demonstrate cleaved caspase-3, lowered SOD2 expression, altered MMP9 and increased TIMP-1 levels. People with COPD have reduced expression of pulmonary and systemic AHR, with systemic AHR mRNA levels positively correlating with lung function. Systemic AHR was also lower in never-smokers with COPD.	[254,321]
Aqueous ragweed pollen extracts	C57BL/6 mouse *AhR*^−/−^, *CYP1A1*^−/−^, *CYP1A2*^−/−^ and *CYP1B1*^−/−^; epithelial cells (ECs)	Enhanced allergic airway inflammation in *AhR*^−/−^ and *CYP1B1*^−/−^ but not *CYP1A1*^−/−^ or *CYP1A2*^−/−^ animals	Enhancement of CYP1A1 activity in *CYP1B1*^−/−^ ECs and alteration of tryptophane (Trp) metabolite levels as a possible mechanism responsible for the allergic airway inflammation.	[257]
Cockroach extract (CRE B46), CH223191 (AhR antagonist)	Mesenchymal stem cells (MSCs), macrophages of C57BL/6 mice	Increased inflammation in CRE-induced asthma	Enhanced mRNA expression of AhR, CYP1a1, and CYP1b1 during asthma pathogenesis increased the expression of M1 macrophage markers. Modulation of macrophage polarization by MSC in part via activation of AhR signaling.	[259]
Indoxyl-3-sulfate (I3S)	C57BL/6 mice	Lessened ovalbumin-sensitized allergic asthma	I3S inhibits Th2 differentiation but shows little or no effect on Th1 differentiation. Effects of I3S on Th2 differentiation are suppressed by AhR antagonist α-naphthoflavone.	[277]
Indoxyl-3-carbinol e	C57BL/6 mice or conditional AhR-deficient mice (DC- or IEC-specific)	Supported oral tolerance against ovalbumin and improved peanut allergy symptoms	I3C activates AhR in the intestine and promotes the creation of an anti-inflammatory environment in the intestine.	[352]
CH223191 (antagonist AhR), siRNA against AhR transfection	Human bronchial epithelial cells, C57BL/6 mice and *AhR*^−/−^ mice on the C57BL/6 background	Protection against cockroach allergen-induced Muc5ac expression and airway inflammation	Role of functional axis of AhR-ROS-NLRP3 inflammasome in regulating Muc5ac expression and airway inflammation.	[287]
CH223191, siRNA transfection	Conditional AhR knockout mice in AT2 cells (Sftpc-Cre;AhRf/f), mouse AT2 cells, human bronchial epithelial cells (HBEC3-KT)	Protection against allergic airway inflammation mouse model of asthma with	Control of autophagy by AhR in alveolar epithelial cells. Autophagy inhibition attenuates cockroach allergen-induced airway inflammation and Th2-associated cytokines. Mechanistically identified a functional axis of AhR-TGF-β1 that is critical in driving allergen-induced cellular autophagy.	[288]
Transfection with siRNA against AhR, Arnt, antagonist α-naphthoflavone, exposure to CXCL8- and CCL5-inducing chemicals	BEAS-2B cells, an SV40 hybrid (Ad12SV40) transformed human bronchial epithelial cell line	regulation of inflammation	Suppression of the activation of p65 by constitutively active, unligated AhR, while it interferes with the action of Arnt-activated p65. AhR signaling may be a weak activator of p65 signaling that suppresses p65 activity induced by strong activators of NF-κB, but its anti-inflammatory properties also are due to interference with additional pathways.	[256]
Mannan-binding lectin (MBL)	Serum from silicosis patients, C57BL/6 mice, MBL-deficient (*MBL*^−/−^) mice	Potential modulating effect of MBL on Th17 immunity	MBL limited the Th17 immunity via controlling the AhR/STAT3 pathway. MBL inhibited the Th17 differentiation through limiting AhR expression and its nuclear translocation. MBL deficiency aggravates silica-induced pulmonary inflammation and fibrosis in mice, which is associated with the regulation of Th17 immunity.	[371]

## Data Availability

No new data were created or analyzed in this study.

## Abbreviations

The following abbreviations are used in this manuscript:

AhR	Aryl hydrocarbon receptor
COPD	Chronic obstructive pulmonary disease
COX2	Cyclooxygenase-2
DAMPs	Damage-associated molecular patterns
DCs	Dendritic cells
EGR1	Early growth response protein 1
FoxP3	Forkhead Box 3
HIF-1α	Hypoxia-inducible factor
I3S	Indoxyl-3-sulfate
IDO	Indoleamine 2,3-dioxygenase
IFN-γ	Interferon-γ
Ig	Immunoglobulin
IGF1R	Insulin-like growth factor 1
ILCs	Innate lymphoid cells
ILD	Interstitial lung disease
JAKs	Janus kinases
KLF6	Kruppel-like factor 6
KYN	Kynurenine
KYNA	Kynurenic acid
LPS	Lipopolysaccharide
MBL	Mannan-binding lectin
MDSCs	Myeloid-derived suppressor cells
MMPs	Matrix metalloproteinases
NF-κB	Nuclear factor κB
NK	Natural killer
NO	Nitric oxide
NRF2	Nuclear factor erythroid 2-related factor 2
PAMPs	Pathogen-associated molecular patterns
PGE2	Prostaglandin E2
PGG2	Prostaglandin G2
PM	Particulate matter
PMN-MDSC	Polymorphonuclear myeloid-derived suppressor cell
PRRs	Pattern recognition receptors
RA	Retinoic acid
ROREs	ROR response elements
RORs	Retinoic Acid-Related Orphan Receptors
RORγt	Retinoic Acid-Related Orphan Receptor γt
ROS	Reactive oxygen species
STAT	Signal Transducer and Activator of Transcription
TADs	Transactivation domains
TCDD	2,3,7,8-tetrachlorodibenzo-p-dioxin
Th	T helper
TLRs	Toll-like receptors
TNFα	Tumor necrosis factor α
Tr1	Type 1 regulatory T cells
Tregs	T-regulatory cells
TSLP	Thymic stromal lymphopoietin
XAP2	X-associated protein-2
XRE	Xenobiotic-responsive element

## References

[1] Beamer C.A., Shepherd D.M. (2013). Role of the aryl hydrocarbon receptor (AhR) in lung inflammation. Semin. Immunopathol..

[2] Cicchese J.M., Evans S., Hult C., Joslyn L.R., Wessler T., Millar J.A., Marino S., Cilfone N.A., Mattila J.T., Linderman J.J. (2018). Dynamic balance of pro- and anti-inflammatory signals controls disease and limits pathology. Immunol. Rev..

[3] Chen L., Deng H., Cui H., Fang J., Zuo Z., Deng J., Li Y., Wang X., Zhao L. (2018). Inflammatory responses and inflammation-associated diseases in organs. Oncotarget.

[4] Huang W., Rui K., Wang X., Peng N., Zhou W., Shi X., Lu L., Hu D., Tian J. (2023). The aryl hydrocarbon receptor in immune regulation and autoimmune pathogenesis. J. Autoimmun..

[5] Quintana F.J., Basso A.S., Iglesias A.H., Korn T., Farez M.F., Bettelli E., Caccamo M., Oukka M., Weiner H.L. (2008). Control of T(reg) and T(H)17 cell differentiation by the aryl hydrocarbon receptor. Nature.

[6] Veldhoen M., Hirota K., Westendorf A.M., Buer J., Dumoutier L., Renauld J.C., Stockinger B. (2008). The aryl hydrocarbon receptor links TH17-cell-mediated autoimmunity to environmental toxins. Nature.

[7] Shinde R., Hezaveh K., Halaby M.J., Kloetgen A., Chakravarthy A., da Silva Medina T., Deol R., Manion K.P., Baglaenko Y., Eldh M. (2018). Apoptotic cell-induced AhR activity is required for immunological tolerance and suppression of systemic lupus erythematosus in mice and humans. Nat. Immunol..

[8] Huang C.H., Chao J.Y., Ling T.C., Wu J.L., Sung J.M., Sun C.Y., Cheng Y.Y., Chang Y.T. (2023). Effect of dialysis modalities on risk of hospitalization for gastrointestinal bleeding. Sci. Rep..

[9] Gould G.S., Hurst J.R., Trofor A., Alison J.A., Fox G., Kulkarni M.M., Wheelock C.E., Clarke M., Kumar R. (2023). Recognising the importance of chronic lung disease: A consensus statement from the Global Alliance for Chronic Diseases (Lung Diseases group). Respir. Res..

[10] Ruvuna L., Sood A. (2020). Epidemiology of Chronic Obstructive Pulmonary Disease. Clin. Chest Med..

[11] Zhao G., Wang L., Lei S., Li Y., Li J., Feng Z. (2025). All-Cause and Cause-Specific Mortality in Chronic Obstructive Pulmonary Disease: A Systematic Review and Meta-Analysis. Int. J. Chron. Obs. Pulmon. Dis..

[12] Miao T.W., Xiao W., Du L.Y., Mao B., Huang W., Chen X.M., Li C., Wang Y., Fu J.J. (2021). High expression of SPP1 in patients with chronic obstructive pulmonary disease (COPD) is correlated with increased risk of lung cancer. FEBS Open Bio.

[13] Passi M., Shahid S., Chockalingam S., Sundar I.K., Packirisamy G. (2020). Conventional and Nanotechnology Based Approaches to Combat Chronic Obstructive Pulmonary Disease: Implications for Chronic Airway Diseases. Int. J. Nanomed..

[14] Adeloye D., Chua S., Lee C., Basquill C., Papana A., Theodoratou E., Nair H., Gasevic D., Sridhar D., Campbell H. (2015). Global and regional estimates of COPD prevalence: Systematic review and meta-analysis. J. Glob. Health.

[15] Lambrecht B.N., Hammad H., Fahy J.V. (2019). The Cytokines of Asthma. Immunity.

[16] Jiang M., Bu W., Wang X., Ruan J., Shi W., Yu S., Huang L., Xue P., Tang J., Zhao X. (2025). Pulmonary fibrosis: From mechanisms to therapies. J. Transl. Med..

[17] Furman D., Campisi J., Verdin E., Carrera-Bastos P., Targ S., Franceschi C., Ferrucci L., Gilroy D.W., Fasano A., Miller G.W. (2019). Chronic inflammation in the etiology of disease across the life span. Nat. Med..

[18] Chavda V.P., Feehan J., Apostolopoulos V. (2024). Inflammation: The Cause of All Diseases. Cells.

[19] Nathan C., Ding A. (2010). Nonresolving inflammation. Cell.

[20] Pahwa R., Goyal A., Jialal I. (2025). Chronic Inflammation. StatPearls.

[21] Zhang Z., Wang A., Wang Y., Sun W., Zhou X., Xu Q., Mao L., Zhang J. (2023). Canthin-6-Ones: Potential Drugs for Chronic Inflammatory Diseases by Targeting Multiple Inflammatory Mediators. Molecules.

[22] Stramer B.M., Mori R., Martin P. (2007). The inflammation-fibrosis link? A Jekyll and Hyde role for blood cells during wound repair. J. Investig. Dermatol..

[23] Wynn T.A., Chawla A., Pollard J.W. (2013). Macrophage biology in development, homeostasis and disease. Nature.

[24] Durai V., Murphy K.M. (2016). Functions of Murine Dendritic Cells. Immunity.

[25] Goudot C., Coillard A., Villani A.C., Gueguen P., Cros A., Sarkizova S., Tang-Huau T.L., Bohec M., Baulande S., Hacohen N. (2017). Aryl Hydrocarbon Receptor Controls Monocyte Differentiation into Dendritic Cells versus Macrophages. Immunity.

[26] Neamah W.H., Singh N.P., Alghetaa H., Abdulla O.A., Chatterjee S., Busbee P.B., Nagarkatti M., Nagarkatti P. (2019). AhR Activation Leads to Massive Mobilization of Myeloid-Derived Suppressor Cells with Immunosuppressive Activity through Regulation of CXCR2 and MicroRNA miR-150-5p and miR-543-3p That Target Anti-Inflammatory Genes. J. Immunol..

[27] Vivier E., Tomasello E., Baratin M., Walzer T., Ugolini S. (2008). Functions of natural killer cells. Nat. Immunol..

[28] Martin F., Chan A.C. (2006). B cell immunobiology in disease: Evolving concepts from the clinic. Annu. Rev. Immunol..

[29] Shao B.Z., Xu Z.Q., Han B.Z., Su D.F., Liu C. (2015). NLRP3 inflammasome and its inhibitors: A review. Front. Pharmacol..

[30] Newton K., Dixit V.M. (2012). Signaling in innate immunity and inflammation. Cold Spring Harb. Perspect. Biol..

[31] Wang N., Liang H., Zen K. (2014). Molecular mechanisms that influence the macrophage m1-m2 polarization balance. Front. Immunol..

[32] Wynn T.A., Vannella K.M. (2016). Macrophages in Tissue Repair, Regeneration, and Fibrosis. Immunity.

[33] Mantovani A., Sica A., Sozzani S., Allavena P., Vecchi A., Locati M. (2004). The chemokine system in diverse forms of macrophage activation and polarization. Trends Immunol..

[34] Mosser D.M. (2003). The many faces of macrophage activation. J. Leukoc. Biol..

[35] Hou F., Xiao K., Tang L., Xie L. (2021). Diversity of Macrophages in Lung Homeostasis and Diseases. Front. Immunol..

[36] Byrne A.J., Mathie S.A., Gregory L.G., Lloyd C.M. (2015). Pulmonary macrophages: Key players in the innate defence of the airways. Thorax.

[37] Bedoret D., Wallemacq H., Marichal T., Desmet C., Quesada Calvo F., Henry E., Closset R., Dewals B., Thielen C., Gustin P. (2009). Lung interstitial macrophages alter dendritic cell functions to prevent airway allergy in mice. J. Clin. Investig..

[38] Cabeza-Cabrerizo M., Cardoso A., Minutti C.M., Pereira da Costa M., Reis e Sousa C. (2021). Dendritic Cells Revisited. Annu. Rev. Immunol..

[39] Quintana F.J., Yeste A., Mascanfroni I.D. (2015). Role and therapeutic value of dendritic cells in central nervous system autoimmunity. Cell Death Differ..

[40] Takenaka M.C., Quintana F.J. (2017). Tolerogenic dendritic cells. Semin. Immunopathol..

[41] Veglia F., Perego M., Gabrilovich D. (2018). Myeloid-derived suppressor cells coming of age. Nat. Immunol..

[42] Mujal A.M., Delconte R.B., Sun J.C. (2021). Natural Killer Cells: From Innate to Adaptive Features. Annu. Rev. Immunol..

[43] Klose C.S.N., Flach M., Mohle L., Rogell L., Hoyler T., Ebert K., Fabiunke C., Pfeifer D., Sexl V., Fonseca-Pereira D. (2014). Differentiation of type 1 ILCs from a common progenitor to all helper-like innate lymphoid cell lineages. Cell.

[44] Zhong C., Cui K., Wilhelm C., Hu G., Mao K., Belkaid Y., Zhao K., Zhu J. (2016). Group 3 innate lymphoid cells continuously require the transcription factor GATA-3 after commitment. Nat. Immunol..

[45] Robinette M.L., Fuchs A., Cortez V.S., Lee J.S., Wang Y., Durum S.K., Gilfillan S., Colonna M., Immunological Genome C. (2015). Transcriptional programs define molecular characteristics of innate lymphoid cell classes and subsets. Nat. Immunol..

[46] Fuchs A., Vermi W., Lee J.S., Lonardi S., Gilfillan S., Newberry R.D., Cella M., Colonna M. (2013). Intraepithelial type 1 innate lymphoid cells are a unique subset of IL-12- and IL-15-responsive IFN-gamma-producing cells. Immunity.

[47] Hoyler T., Klose C.S., Souabni A., Turqueti-Neves A., Pfeifer D., Rawlins E.L., Voehringer D., Busslinger M., Diefenbach A. (2012). The transcription factor GATA-3 controls cell fate and maintenance of type 2 innate lymphoid cells. Immunity.

[48] Klein Wolterink R.G., Serafini N., van Nimwegen M., Vosshenrich C.A., de Bruijn M.J., Fonseca Pereira D., Veiga Fernandes H., Hendriks R.W., Di Santo J.P. (2013). Essential, dose-dependent role for the transcription factor Gata3 in the development of IL-5+ and IL-13+ type 2 innate lymphoid cells. Proc. Natl. Acad. Sci. USA.

[49] Satoh-Takayama N., Vosshenrich C.A., Lesjean-Pottier S., Sawa S., Lochner M., Rattis F., Mention J.J., Thiam K., Cerf-Bensussan N., Mandelboim O. (2008). Microbial flora drives interleukin 22 production in intestinal NKp46+ cells that provide innate mucosal immune defense. Immunity.

[50] Rankin L.C., Groom J.R., Chopin M., Herold M.J., Walker J.A., Mielke L.A., McKenzie A.N., Carotta S., Nutt S.L., Belz G.T. (2013). The transcription factor T-bet is essential for the development of NKp46+ innate lymphocytes via the Notch pathway. Nat. Immunol..

[51] Buonocore S., Ahern P.P., Uhlig H.H., Ivanov I.I., Littman D.R., Maloy K.J., Powrie F. (2010). Innate lymphoid cells drive interleukin-23-dependent innate intestinal pathology. Nature.

[52] Lo B., Hansen S., Evans K., Heath J.K., Wright J.R. (2008). Alveolar epithelial type II cells induce T cell tolerance to specific antigen. J. Immunol..

[53] Debbabi H., Ghosh S., Kamath A.B., Alt J., Demello D.E., Dunsmore S., Behar S.M. (2005). Primary type II alveolar epithelial cells present microbial antigens to antigen-specific CD4^+^ T cells. Am. J. Physiol. Lung Cell. Mol. Physiol..

[54] Dong C. (2008). TH17 cells in development: An updated view of their molecular identity and genetic programming. Nat. Rev. Immunol..

[55] Zeng J., Li M., Zhao Q., Chen M., Zhao L., Wei S., Yang H., Zhao Y., Wang A., Shen J. (2023). Small molecule inhibitors of RORgammat for Th17 regulation in inflammatory and autoimmune diseases. J. Pharm. Anal..

[56] Schnell A., Littman D.R., Kuchroo V.K. (2023). T(H)17 cell heterogeneity and its role in tissue inflammation. Nat. Immunol..

[57] Georgiev P., Charbonnier L.M., Chatila T.A. (2019). Regulatory T Cells: The Many Faces of Foxp3. J. Clin. Immunol..

[58] Benamar M., Chen Q., Martinez-Blanco M., Chatila T.A. (2023). Regulatory T cells in allergic inflammation. Semin. Immunol..

[59] Stavnezer J., Guikema J.E., Schrader C.E. (2008). Mechanism and regulation of class switch recombination. Annu. Rev. Immunol..

[60] Gopallawa I., Dehinwal R., Bhatia V., Gujar V., Chirmule N. (2023). A four-part guide to lung immunology: Invasion, inflammation, immunity, and intervention. Front. Immunol..

[61] Parker D., Prince A. (2011). Innate immunity in the respiratory epithelium. Am. J. Respir. Cell. Mol. Biol..

[62] Cole A.M., Waring A.J. (2002). The role of defensins in lung biology and therapy. Am. J. Respir. Med..

[63] Huang C., Sali A., Stevens R.L. (1998). Regulation and function of mast cell proteases in inflammation. J. Clin. Immunol..

[64] Wan M., Wang C., Cui J., Xia Q., Zhang L. (2024). Silencing KLF6 Alleviates Cigarette Smoke Extract-Induced Mitochondrial Dysfunction in Bronchial Epithelial Cells by SIRT4 Upregulation. Int. J. Chron. Obs. Pulmon. Dis..

[65] Gudernatsch V., Stefanczyk S.A., Mirakaj V. (2020). Novel Resolution Mediators of Severe Systemic Inflammation. Immunotargets Ther..

[66] McIntosh B.E., Hogenesch J.B., Bradfield C.A. (2010). Mammalian Per-Arnt-Sim proteins in environmental adaptation. Annu. Rev. Physiol..

[67] Nebert D.W. (2017). Aryl hydrocarbon receptor (AHR): “pioneer member” of the basic-helix/loop/helix per-Arnt-sim (bHLH/PAS) family of “sensors” of foreign and endogenous signals. Prog. Lipid Res..

[68] Kolonko-Adamska M., Uversky V.N., Greb-Markiewicz B. (2021). The Participation of the Intrinsically Disordered Regions of the bHLH-PAS Transcription Factors in Disease Development. Int. J. Mol. Sci..

[69] Schulte K.W., Green E., Wilz A., Platten M., Daumke O. (2017). Structural Basis for Aryl Hydrocarbon Receptor-Mediated Gene Activation. Structure.

[70] Sahoo M., Samanta L., Satpathy R. (2025). Exploring the role of toxic and therapeutic compounds in aryl hydrocarbon receptor (AhR) modulation: Insights from molecular modeling study. Discov. Chem..

[71] Yamamoto J., Ihara K., Nakayama H., Hikino S., Satoh K., Kubo N., Iida T., Fujii Y., Hara T. (2004). Characteristic expression of aryl hydrocarbon receptor repressor gene in human tissues: Organ-specific distribution and variable induction patterns in mononuclear cells. Life Sci..

[72] Pollenz R.S., Barbour E.R. (2000). Analysis of the complex relationship between nuclear export and aryl hydrocarbon receptor-mediated gene regulation. Mol. Cell. Biol..

[73] Fujii-Kuriyama Y., Kawajiri K. (2010). Molecular mechanisms of the physiological functions of the aryl hydrocarbon (dioxin) receptor, a multifunctional regulator that senses and responds to environmental stimuli. Proc. Jpn. Acad. Ser. B Phys. Biol. Sci..

[74] Haidar R., Shabo R., Moeser M., Luch A., Kugler J. (2023). The nuclear entry of the aryl hydrocarbon receptor (AHR) relies on the first nuclear localization signal and can be negatively regulated through IMPalpha/beta specific inhibitors. Sci. Rep..

[75] Tsuji N., Fukuda K., Nagata Y., Okada H., Haga A., Hatakeyama S., Yoshida S., Okamoto T., Hosaka M., Sekine K. (2014). The activation mechanism of the aryl hydrocarbon receptor (AhR) by molecular chaperone HSP90. FEBS Open Bio.

[76] Larigot L., Juricek L., Dairou J., Coumoul X. (2018). AhR signaling pathways and regulatory functions. Biochim. Open.

[77] Whitlock J.P. (1999). Induction of cytochrome P4501A1. Annu. Rev. Pharmacol. Toxicol..

[78] Meyer B.K., Pray-Grant M.G., Vanden Heuvel J.P., Perdew G.H. (1998). Hepatitis B virus X-associated protein 2 is a subunit of the unliganded aryl hydrocarbon receptor core complex and exhibits transcriptional enhancer activity. Mol. Cell. Biol..

[79] Rudyak S.G., Usakin L.A., Tverye E.A., Robertson E.D., Panteleyev A.A. (2023). Aryl hydrocarbon receptor is regulated via multiple mechanisms in human keratinocytes. Toxicol. Lett..

[80] Pollenz R.S. (2002). The mechanism of AH receptor protein down-regulation (degradation) and its impact on AH receptor-mediated gene regulation. Chem. Biol. Interact..

[81] Opitz C.A., Holfelder P., Prentzell M.T., Trump S. (2023). The complex biology of aryl hydrocarbon receptor activation in cancer and beyond. Biochem. Pharmacol..

[82] Hahn M.E., Allan L.L., Sherr D.H. (2009). Regulation of constitutive and inducible AHR signaling: Complex interactions involving the AHR repressor. Biochem. Pharmacol..

[83] Wuputra K., Ku C.C., Hsu W.H., Hsieh T.J., Tsai Y.C., Chen C.Y., Tanaka Y., Lin Y.C., Kuo C.H., Wu D.C. (2025). New insights into coordinated regulation of AHR promoter transcription; molecular mechanisms and therapeutic targets. Int. J. Biol. Sci..

[84] Denison M.S., Fisher J.M., Whitlock J.P. (1988). The DNA Recognition Site for the Dioxin-Ah Receptor Complex—Nucleotide-Sequence and Functional-Analysis. J. Biol. Chem..

[85] Denison M.S., Nagy S.R. (2003). Activation of the aryl hydrocarbon receptor by structurally diverse exogenous and endogenous chemicals. Annu. Rev. Pharmacol. Toxicol..

[86] Delescluse C., Lemaire G., de Sousa G., Rahmani R. (2000). Is CYP1A1 induction always related to AHR signaling pathway?. Toxicology.

[87] Abbott B.D., Perdew G.H., Buckalew A.R., Birnbaum L.S. (1994). Interactive Regulation of Ah and Glucocorticoid Receptors in the Synergistic Induction of Cleft-Palate by 2,3,7,8-Tetrachlorodibenzo-P-Dioxin and Hydrocortisone. Toxicol. Appl. Pharmacol..

[88] Birnbaum L.S. (1994). The Mechanism of Dioxin Toxicity—Relationship to Risk Assessment. Environ. Health Perspect..

[89] Ma Q., Baldwin K.T. (2000). 2,3,7,8-tetrachlorodibenzo-p-dioxin-induced degradation of aryl hydrocarbon receptor (AhR) by the ubiquitin-proteasome pathway—Role of the transcription activaton and DNA binding of AhR. J. Biol. Chem..

[90] Rothhammer V., Quintana F.J. (2019). The aryl hydrocarbon receptor: An environmental sensor integrating immune responses in health and disease. Nat. Rev. Immunol..

[91] Murray I.A., Patterson A.D., Perdew G.H. (2014). Aryl hydrocarbon receptor ligands in cancer: Friend and foe. Nat. Rev. Cancer.

[92] Hubbard T.D., Murray I.A., Perdew G.H. (2015). Indole and Tryptophan Metabolism: Endogenous and Dietary Routes to Ah Receptor Activation. Drug Metab. Dispos..

[93] Wirthgen E., Hoeflich A., Rebl A., Gunther J. (2017). Kynurenic Acid: The Janus-Faced Role of an Immunomodulatory Tryptophan Metabolite and Its Link to Pathological Conditions. Front. Immunol..

[94] Smirnova A., Wincent E., Vikstrom Bergander L., Alsberg T., Bergman J., Rannug A., Rannug U. (2016). Evidence for New Light-Independent Pathways for Generation of the Endogenous Aryl Hydrocarbon Receptor Agonist FICZ. Chem. Res. Toxicol..

[95] Anderson G., Maes M. (2017). Interactions of Tryptophan and Its Catabolites With Melatonin and the Alpha 7 Nicotinic Receptor in Central Nervous System and Psychiatric Disorders: Role of the Aryl Hydrocarbon Receptor and Direct Mitochondria Regulation. Int. J. Tryptophan Res..

[96] Schaldach C.M., Riby J., Bjeldanes L.F. (1999). Lipoxin A4: A new class of ligand for the Ah receptor. Biochemistry.

[97] Phelan D., Winter G.M., Rogers W.J., Lam J.C., Denison M.S. (1998). Activation of the Ah receptor signal transduction pathway by bilirubin and biliverdin. Arch. Biochem. Biophys..

[98] Perkins A., Phillips J.L., Kerkvliet N.I., Tanguay R.L., Perdew G.H., Kolluri S.K., Bisson W.H. (2014). A Structural Switch between Agonist and Antagonist Bound Conformations for a Ligand-Optimized Model of the Human Aryl Hydrocarbon Receptor Ligand Binding Domain. Biology.

[99] Safe S., Jin U.H., Park H., Chapkin R.S., Jayaraman A. (2020). Aryl Hydrocarbon Receptor (AHR) Ligands as Selective AHR Modulators (SAhRMs). Int. J. Mol. Sci..

[100] Perdew G.H. (1988). Association of the Ah receptor with the 90-kDa heat shock protein. J. Biol. Chem..

[101] Kazlauskas A., Poellinger L., Pongratz I. (1999). Evidence that the co-chaperone p23 regulates ligand responsiveness of the dioxin (Aryl hydrocarbon) receptor. J. Biol. Chem..

[102] Enan E., Matsumura F. (1996). Identification of c-Src as the integral component of the cytosolic Ah receptor complex, transducing the signal of 2,3,7,8-tetrachlorodibenzo-p-dioxin (TCDD) through the protein phosphorylation pathway. Biochem. Pharmacol..

[103] Pongratz I., Mason G.G., Poellinger L. (1992). Dual roles of the 90-kDa heat shock protein hsp90 in modulating functional activities of the dioxin receptor. Evidence that the dioxin receptor functionally belongs to a subclass of nuclear receptors which require hsp90 both for ligand binding activity and repression of intrinsic DNA binding activity. J. Biol. Chem..

[104] Ikuta T., Eguchi H., Tachibana T., Yoneda Y., Kawajiri K. (1998). Nuclear localization and export signals of the human aryl hydrocarbon receptor. J. Biol. Chem..

[105] Kudo I., Hosaka M., Haga A., Tsuji N., Nagata Y., Okada H., Fukuda K., Kakizaki Y., Okamoto T., Grave E. (2018). The regulation mechanisms of AhR by molecular chaperone complex. J. Biochem..

[106] Ma Q. (2001). Induction of CYP1A1. The AhR/DRE paradigm: Transcription, receptor regulation, and expanding biological roles. Curr. Drug Metab..

[107] Kawajiri K., Fujii-Kuriyama Y. (2017). The aryl hydrocarbon receptor: A multifunctional chemical sensor for host defense and homeostatic maintenance. Exp. Anim..

[108] Puga A., Barnes S.J., Dalton T.P., Chang C., Knudsen E.S., Maier M.A. (2000). Aromatic hydrocarbon receptor interaction with the retinoblastoma protein potentiates repression of E2F-dependent transcription and cell cycle arrest. J. Biol. Chem..

[109] Go R.E., Hwang K.A., Choi K.C. (2015). Cytochrome P450 1 family and cancers. J. Steroid Biochem. Mol. Biol..

[110] Kuramoto N., Baba K., Gion K., Sugiyama C., Taniura H., Yoneda Y. (2003). Xenobiotic response element binding enriched in both nuclear and microsomal fractions of rat cerebellum. J. Neurochem..

[111] Jackson D.P., Joshi A.D., Elferink C.J. (2015). Ah Receptor Pathway Intricacies; Signaling Through Diverse Protein Partners and DNA-Motifs. Toxicol. Res..

[112] Beischlag T.V., Luis Morales J., Hollingshead B.D., Perdew G.H. (2008). The aryl hydrocarbon receptor complex and the control of gene expression. Crit. Rev. Eukaryot. Gene Expr..

[113] Joshi A.D., Hossain E., Elferink C.J. (2017). Epigenetic Regulation by Agonist-Specific Aryl Hydrocarbon Receptor Recruitment of Metastasis-Associated Protein 2 Selectively Induces Stanniocalcin 2 Expression. Mol. Pharmacol..

[114] Habano W., Miura T., Terashima J., Ozawa S. (2022). Aryl hydrocarbon receptor as a DNA methylation reader in the stress response pathway. Toxicology.

[115] Jiang J., Zhu J., Liu Q., Zhang T., Wen J., Xia J., Deng Y. (2021). Role of DNA methylation-related chromatin remodeling in aryl hydrocarbon receptor-dependent regulation of T-2 toxin highly inducible Cytochrome P450 1A4 gene. FASEB J..

[116] Mulero-Navarro S., Fernandez-Salguero P.M. (2016). New Trends in Aryl Hydrocarbon Receptor Biology. Front. Cell Dev. Biol..

[117] Singh N.P., Singh U.P., Singh B., Price R.L., Nagarkatti M., Nagarkatti P.S. (2011). Activation of aryl hydrocarbon receptor (AhR) leads to reciprocal epigenetic regulation of FoxP3 and IL-17 expression and amelioration of experimental colitis. PLoS ONE.

[118] Tian J., Feng Y., Fu H., Xie H.Q., Jiang J.X., Zhao B. (2015). The Aryl Hydrocarbon Receptor: A Key Bridging Molecule of External and Internal Chemical Signals. Environ. Sci. Technol..

[119] Grishanova A.Y., Klyushova L.S., Perepechaeva M.L. (2023). AhR and Wnt/beta-Catenin Signaling Pathways and Their Interplay. Curr. Issues Mol. Biol..

[120] Gorelik A., Dukic N., Smith R., Chatrin C., Suyari O., Matthews J., Ahel I. (2026). Ubiquitin pathway blockade reveals endogenous ADP-ribosylation marking PARP7 and AHR for degradation. EMBO J..

[121] Hahn M.E., Sherr D.H. (2024). The enigmatic AHRR: Beyond aryl hydrocarbon receptor repression. J. Leukoc. Biol..

[122] Grimaldi G., Rajendra S., Matthews J. (2018). The aryl hydrocarbon receptor regulates the expression of TIPARP and its cis long non-coding RNA, TIPARP-AS1. Biochem. Biophys. Res. Commun..

[123] Chen H., Gou X., Mao Y., O’Leary P.C., Diolaiti M.E., Ashworth A. (2025). PARP7 Inhibitors and AHR Agonists Act Synergistically across a Wide Range of Cancer Models. Mol. Cancer Ther..

[124] Hao N., Whitelaw M.L. (2013). The emerging roles of AhR in physiology and immunity. Biochem. Pharmacol..

[125] Esser C., Rannug A. (2015). The aryl hydrocarbon receptor in barrier organ physiology, immunology, and toxicology. Pharmacol. Rev..

[126] Denison M.S., Faber S.C. (2017). And Now for Something Completely Different: Diversity in Ligand-Dependent Activation of Ah Receptor Responses. Curr. Opin. Toxicol..

[127] Wilson S.R., Joshi A.D., Elferink C.J. (2013). The tumor suppressor Kruppel-like factor 6 is a novel aryl hydrocarbon receptor DNA binding partner. J. Pharmacol. Exp. Ther..

[128] Chang X., Fan Y., Karyala S., Schwemberger S., Tomlinson C.R., Sartor M.A., Puga A. (2007). Ligand-independent regulation of transforming growth factor beta1 expression and cell cycle progression by the aryl hydrocarbon receptor. Mol. Cell. Biol..

[129] Xiao W., Son J., Vorrink S.U., Domann F.E., Goswami P.C. (2015). Ligand-independent activation of aryl hydrocarbon receptor signaling in PCB3-quinone treated HaCaT human keratinocytes. Toxicol. Lett..

[130] Coumoul X., Barouki R., Esser C., Haarmann-Stemmann T., Lawrence B.P., Lehmann J., Moura-Alves P., Murray I.A., Opitz C.A., Perdew G.H. (2026). The aryl hydrocarbon receptor: Structure, signaling, physiology and pathology. Signal Transduct. Target. Ther..

[131] Sondermann N.C., Fassbender S., Hartung F., Hatala A.M., Rolfes K.M., Vogel C.F.A., Haarmann-Stemmann T. (2023). Functions of the aryl hydrocarbon receptor (AHR) beyond the canonical AHR/ARNT signaling pathway. Biochem. Pharmacol..

[132] Puga A., Ma C., Marlowe J.L. (2009). The aryl hydrocarbon receptor cross-talks with multiple signal transduction pathways. Biochem. Pharmacol..

[133] Button E.L., Bersten D.C., Whitelaw M.L. (2017). HIF has Biff—Crosstalk between HIF1a and the family of bHLH/PAS proteins. Exp. Cell Res..

[134] Vorrink S.U., Domann F.E. (2014). Regulatory crosstalk and interference between the xenobiotic and hypoxia sensing pathways at the AhR-ARNT-HIF1 alpha signaling node. Chem. -Biol. Interact..

[135] Vogel C.F., Wu D., Goth S.R., Baek J., Lollies A., Domhardt R., Grindel A., Pessah I.N. (2013). Aryl hydrocarbon receptor signaling regulates NF-kappaB RelB activation during dendritic-cell differentiation. Immunol. Cell Biol..

[136] Zhang Q., Lenardo M.J., Baltimore D. (2017). 30 Years of NF-kappaB: A Blossoming of Relevance to Human Pathobiology. Cell.

[137] Ishihara Y., Kado S.Y., Hoeper C., Harel S., Vogel C.F.A. (2019). Role of NF-kB RelB in Aryl Hydrocarbon Receptor-Mediated Ligand Specific Effects. Int. J. Mol. Sci..

[138] Kim D.W., Gazourian L., Quadri S.A., Romieu-Mourez R., Sherr D.H., Sonenshein G.E. (2000). The RelA NF-kappaB subunit and the aryl hydrocarbon receptor (AhR) cooperate to transactivate the c-myc promoter in mammary cells. Oncogene.

[139] Kimura A., Naka T., Nohara K., Fujii-Kuriyama Y., Kishimoto T. (2008). Aryl hydrocarbon receptor regulates Stat1 activation and participates in the development of Th17 cells. Proc. Natl. Acad. Sci. USA.

[140] Vogel C.F., Khan E.M., Leung P.S., Gershwin M.E., Chang W.L., Wu D., Haarmann-Stemmann T., Hoffmann A., Denison M.S. (2014). Cross-talk between aryl hydrocarbon receptor and the inflammatory response: A role for nuclear factor-kappaB. J. Biol. Chem..

[141] Vogel C.F., Matsumura F. (2009). A new cross-talk between the aryl hydrocarbon receptor and RelB, a member of the NF-kappaB family. Biochem. Pharmacol..

[142] Guarnieri T. (2020). Aryl Hydrocarbon Receptor Connects Inflammation to Breast Cancer. Int. J. Mol. Sci..

[143] Chiba T., Uchi H., Yasukawa F., Furue M. (2011). Role of the arylhydrocarbon receptor in lung disease. Int. Arch. Allergy Immunol..

[144] Trikha P., Lee D.A. (2020). The role of AhR in transcriptional regulation of immune cell development and function. Biochim. Biophys. Acta Rev. Cancer.

[145] Kerkvliet N.I. (2009). AHR-mediated immunomodulation: The role of altered gene transcription. Biochem. Pharmacol..

[146] Pot C. (2012). Aryl hydrocarbon receptor controls regulatory CD4^+^ T cell function. Swiss Med. Wkly..

[147] Stockinger B., Di Meglio P., Gialitakis M., Duarte J.H. (2014). The aryl hydrocarbon receptor: Multitasking in the immune system. Annu. Rev. Immunol..

[148] Veldhoen M., Hirota K., Christensen J., O’Garra A., Stockinger B. (2009). Natural agonists for aryl hydrocarbon receptor in culture medium are essential for optimal differentiation of Th17 T cells. J. Exp. Med..

[149] Stockinger B., Hirota K., Duarte J., Veldhoen M. (2011). External influences on the immune system via activation of the aryl hydrocarbon receptor. Semin. Immunol..

[150] Riaz F., Pan F., Wei P. (2022). Aryl hydrocarbon receptor: The master regulator of immune responses in allergic diseases. Front. Immunol..

[151] Li J., Phadnis-Moghe A.S., Crawford R.B., Kaminski N.E. (2017). Aryl hydrocarbon receptor activation by 2,3,7,8-tetrachlorodibenzo-p-dioxin impairs human B lymphopoiesis. Toxicology.

[152] Zhou Y., Tung H.Y., Tsai Y.M., Hsu S.C., Chang H.W., Kawasaki H., Tseng H.C., Plunkett B., Gao P., Hung C.H. (2013). Aryl hydrocarbon receptor controls murine mast cell homeostasis. Blood.

[153] Kawasaki H., Chang H.W., Tseng H.C., Hsu S.C., Yang S.J., Hung C.H., Zhou Y., Huang S.K. (2014). A tryptophan metabolite, kynurenine, promotes mast cell activation through aryl hydrocarbon receptor. Allergy.

[154] Nguyen N.T., Kimura A., Nakahama T., Chinen I., Masuda K., Nohara K., Fujii-Kuriyama Y., Kishimoto T. (2010). Aryl hydrocarbon receptor negatively regulates dendritic cell immunogenicity via a kynurenine-dependent mechanism. Proc. Natl. Acad. Sci. USA.

[155] Li Y., Innocentin S., Withers D.R., Roberts N.A., Gallagher A.R., Grigorieva E.F., Wilhelm C., Veldhoen M. (2011). Exogenous stimuli maintain intraepithelial lymphocytes via aryl hydrocarbon receptor activation. Cell.

[156] Dean J.W., Zhou L. (2022). Cell-intrinsic view of the aryl hydrocarbon receptor in tumor immunity. Trends Immunol..

[157] Baricza E., Tamasi V., Marton N., Buzas E.I., Nagy G. (2016). The emerging role of aryl hydrocarbon receptor in the activation and differentiation of Th17 cells. Cell. Mol. Life Sci..

[158] Stone T.W., Williams R.O. (2023). Modulation of T cells by tryptophan metabolites in the kynurenine pathway. Trends Pharmacol. Sci..

[159] Mascanfroni I.D., Takenaka M.C., Yeste A., Patel B., Wu Y., Kenison J.E., Siddiqui S., Basso A.S., Otterbein L.E., Pardoll D.M. (2015). Metabolic control of type 1 regulatory T cell differentiation by AHR and HIF1-alpha. Nat. Med..

[160] Takami M., Fujimaki K., Nishimura M.I., Iwashima M. (2015). Cutting Edge: AhR Is a Molecular Target of Calcitriol in Human T Cells. J. Immunol..

[161] Mandal P.K. (2005). Dioxin: A review of its environmental effects and its aryl hydrocarbon receptor biology. J. Comp. Physiol. B.

[162] Ambrosio L.F., Volpini X., Quiroz J.N., Brugo M.B., Knubel C.P., Herrera M.R., Fozzatti L., Avila Pacheco J., Clish C.B., Takenaka M.C. (2023). Association between altered tryptophan metabolism, plasma aryl hydrocarbon receptor agonists, and inflammatory Chagas disease. Front. Immunol..

[163] Thatcher T.H., Williams M.A., Pollock S.J., McCarthy C.E., Lacy S.H., Phipps R.P., Sime P.J. (2016). Endogenous ligands of the aryl hydrocarbon receptor regulate lung dendritic cell function. Immunology.

[164] Poulain-Godefroy O., Boute M., Carrard J., Alvarez-Simon D., Tsicopoulos A., de Nadai P. (2020). The Aryl Hydrocarbon Receptor in Asthma: Friend or Foe?. Int. J. Mol. Sci..

[165] Bankoti J., Rase B., Simones T., Shepherd D.M. (2010). Functional and phenotypic effects of AhR activation in inflammatory dendritic cells. Toxicol. Appl. Pharmacol..

[166] Quintana F.J., Murugaiyan G., Farez M.F., Mitsdoerffer M., Tukpah A.M., Burns E.J., Weiner H.L. (2010). An endogenous aryl hydrocarbon receptor ligand acts on dendritic cells and T cells to suppress experimental autoimmune encephalomyelitis. Proc. Natl. Acad. Sci. USA.

[167] Li S., Bostick J.W., Zhou L. (2017). Regulation of Innate Lymphoid Cells by Aryl Hydrocarbon Receptor. Front. Immunol..

[168] Marshall N.B., Kerkvliet N.I. (2010). Dioxin and immune regulation: Emerging role of aryl hydrocarbon receptor in the generation of regulatory T cells. Ann. N. Y. Acad. Sci..

[169] Kalinski P. (2012). Regulation of immune responses by prostaglandin E2. J. Immunol..

[170] Fiore A., Murray P.J. (2021). Tryptophan and indole metabolism in immune regulation. Curr. Opin. Immunol..

[171] Wagage S., Hunter C.A. (2015). Interrelated Roles for the Aryl Hydrocarbon Receptor and Hypoxia Inducible Factor-1α in the Immune Response to Infection. Curr. Med. Chem..

[172] Wourms M.J., Sulentic C.E. (2015). The aryl hydrocarbon receptor regulates an essential transcriptional element in the immunoglobulin heavy chain gene. Cell. Immunol..

[173] Sulentic C.E., Kaminski N.E. (2011). The long winding road toward understanding the molecular mechanisms for B-cell suppression by 2,3,7,8-tetrachlorodibenzo-p-dioxin. Toxicol. Sci..

[174] Blevins L.K., Zhou J., Crawford R.B., Kaminski N.E. (2021). Identification of a Sensitive Human Immunological Target of Aryl Hydrocarbon Receptor Activation: CD5(+) Innate-Like B Cells. Front. Immunol..

[175] Snyder A.D., Ochs S.D., Johnson B.E., Sulentic C.E.W. (2020). Aryl hydrocarbon receptor-induced activation of the human IGH hs1.2 enhancer: Mutational analysis of putative regulatory binding motifs. Mol. Immunol..

[176] Qiu J., Heller J.J., Guo X., Chen Z.M., Fish K., Fu Y.X., Zhou L. (2012). The aryl hydrocarbon receptor regulates gut immunity through modulation of innate lymphoid cells. Immunity.

[177] Lee J.S., Cella M., McDonald K.G., Garlanda C., Kennedy G.D., Nukaya M., Mantovani A., Kopan R., Bradfield C.A., Newberry R.D. (2011). AHR drives the development of gut ILC22 cells and postnatal lymphoid tissues via pathways dependent on and independent of Notch. Nat. Immunol..

[178] Kiss E.A., Vonarbourg C., Kopfmann S., Hobeika E., Finke D., Esser C., Diefenbach A. (2011). Natural aryl hydrocarbon receptor ligands control organogenesis of intestinal lymphoid follicles. Science.

[179] Li S., Bostick J.W., Ye J., Qiu J., Zhang B., Urban J.F., Avram D., Zhou L. (2018). Aryl Hydrocarbon Receptor Signaling Cell Intrinsically Inhibits Intestinal Group 2 Innate Lymphoid Cell Function. Immunity.

[180] Wen Y., Zeng Q., Luo X., Ma R., Tang Y., Liu W. (2020). Leptin Promoted IL-17 Production from ILC2s in Allergic Rhinitis. Mediat. Inflamm..

[181] Cai T., Qiu J., Ji Y., Li W., Ding Z., Suo C., Chang J., Wang J., He R., Qian Y. (2019). IL-17-producing ST2(+) group 2 innate lymphoid cells play a pathogenic role in lung inflammation. J. Allergy Clin. Immunol..

[182] Hughes T., Briercheck E.L., Freud A.G., Trotta R., McClory S., Scoville S.D., Keller K., Deng Y., Cole J., Harrison N. (2014). The transcription Factor AHR prevents the differentiation of a stage 3 innate lymphoid cell subset to natural killer cells. Cell Rep..

[183] NF-kB Transcription Factors—Inducers Physical Stress. http://www.bu.edu/nf-kb/physiological-mediators/inducers.

[184] Liu T., Zhang L., Joo D., Sun S.C. (2017). NF-kappaB signaling in inflammation. Signal Transduct. Target. Ther..

[185] Almowallad S., Alqahtani L.S., Mobashir M. (2022). NF-kB in Signaling Patterns and Its Temporal Dynamics Encode/Decode Human Diseases. Life.

[186] Chen P.H., Chang H., Chang J.T., Lin P. (2012). Aryl hydrocarbon receptor in association with RelA modulates IL-6 expression in non-smoking lung cancer. Oncogene.

[187] Takenaka M.C., Gabriely G., Rothhammer V., Mascanfroni I.D., Wheeler M.A., Chao C.C., Gutierrez-Vazquez C., Kenison J., Tjon E.C., Barroso A. (2019). Control of tumor-associated macrophages and T cells in glioblastoma via AHR and CD39. Nat. Neurosci..

[188] Yang X.O., Nurieva R., Martinez G.J., Kang H.S., Chung Y., Pappu B.P., Shah B., Chang S.H., Schluns K.S., Watowich S.S. (2008). Molecular antagonism and plasticity of regulatory and inflammatory T cell programs. Immunity.

[189] Wolf J., Rose-John S., Garbers C. (2014). Interleukin-6 and its receptors: A highly regulated and dynamic system. Cytokine.

[190] Babon J.J., Varghese L.N., Nicola N.A. (2014). Inhibition of IL-6 family cytokines by SOCS3. Semin. Immunol..

[191] Hu X., Li J., Fu M., Zhao X., Wang W. (2021). The JAK/STAT signaling pathway: From bench to clinic. Signal Transduct. Target. Ther..

[192] Bahman F., Choudhry K., Al-Rashed F., Al-Mulla F., Sindhu S., Ahmad R. (2024). Aryl hydrocarbon receptor: Current perspectives on key signaling partners and immunoregulatory role in inflammatory diseases. Front. Immunol..

[193] Grishanova A.Y., Perepechaeva M.L. (2024). Kynurenic Acid/AhR Signaling at the Junction of Inflammation and Cardiovascular Diseases. Int. J. Mol. Sci..

[194] Shadboorestan A., Koual M., Dairou J., Coumoul X. (2023). The Role of the Kynurenine/AhR Pathway in Diseases Related to Metabolism and Cancer. Int. J. Tryptophan Res..

[195] Bessede A., Gargaro M., Pallotta M.T., Matino D., Servillo G., Brunacci C., Bicciato S., Mazza E.M., Macchiarulo A., Vacca C. (2014). Aryl hydrocarbon receptor control of a disease tolerance defence pathway. Nature.

[196] Guarnieri T., Abruzzo P.M., Bolotta A. (2020). More than a cell biosensor: Aryl hydrocarbon receptor at the intersection of physiology and inflammation. Am. J. Physiol. Cell Physiol..

[197] Kimura A., Naka T., Nakahama T., Chinen I., Masuda K., Nohara K., Fujii-Kuriyama Y., Kishimoto T. (2009). Aryl hydrocarbon receptor in combination with Stat1 regulates LPS-induced inflammatory responses. J. Exp. Med..

[198] Trujillo-Ochoa J.L., Kazemian M., Afzali B. (2023). The role of transcription factors in shaping regulatory T cell identity. Nat. Rev. Immunol..

[199] Quintana F.J. (2013). The aryl hydrocarbon receptor: A molecular pathway for the environmental control of the immune response. Immunology.

[200] Wang X.S., Cao F., Zhang Y., Pan H.F. (2020). Therapeutic potential of aryl hydrocarbon receptor in autoimmunity. Inflammopharmacology.

[201] Funatake C.J., Marshall N.B., Steppan L.B., Mourich D.V., Kerkvliet N.I. (2005). Cutting edge: Activation of the aryl hydrocarbon receptor by 2,3,7,8-tetrachlorodibenzo-p-dioxin generates a population of CD4^+^ CD25^+^ cells with characteristics of regulatory T cells. J. Immunol..

[202] Joller N., Lozano E., Burkett P.R., Patel B., Xiao S., Zhu C., Xia J., Tan T.G., Sefik E., Yajnik V. (2014). Treg cells expressing the coinhibitory molecule TIGIT selectively inhibit proinflammatory Th1 and Th17 cell responses. Immunity.

[203] Gutierrez-Vazquez C., Quintana F.J. (2018). Regulation of the Immune Response by the Aryl Hydrocarbon Receptor. Immunity.

[204] Apetoh L., Quintana F.J., Pot C., Joller N., Xiao S., Kumar D., Burns E.J., Sherr D.H., Weiner H.L., Kuchroo V.K. (2010). The aryl hydrocarbon receptor interacts with c-Maf to promote the differentiation of type 1 regulatory T cells induced by IL-27. Nat. Immunol..

[205] Imbratta C., Hussein H., Andris F., Verdeil G. (2020). c-MAF, a Swiss Army Knife for Tolerance in Lymphocytes. Front. Immunol..

[206] Tian Y., Wu Y., Ni B. (2015). Signaling Pathways and Epigenetic Regulations in the Control of RORgammat Expression in T Helper 17 Cells. Int. Rev. Immunol..

[207] Rutz S., Noubade R., Eidenschenk C., Ota N., Zeng W., Zheng Y., Hackney J., Ding J., Singh H., Ouyang W. (2011). Transcription factor c-Maf mediates the TGF-beta-dependent suppression of IL-22 production in T(H)17 cells. Nat. Immunol..

[208] Cook D.N., Kang H.S., Jetten A.M. (2015). Retinoic Acid-Related Orphan Receptors (RORs): Regulatory Functions in Immunity, Development, Circadian Rhythm, and Metabolism. Nucl. Recept. Res..

[209] Qiu J., Zhou L. (2013). Aryl hydrocarbon receptor promotes RORgammat(+) group 3 ILCs and controls intestinal immunity and inflammation. Semin. Immunopathol..

[210] Kumar R., Theiss A.L., Venuprasad K. (2021). RORgammat protein modifications and IL-17-mediated inflammation. Trends Immunol..

[211] Zhou L. (2016). AHR Function in Lymphocytes: Emerging Concepts. Trends Immunol..

[212] Helm E.Y., Zhou L. (2023). Transcriptional regulation of innate lymphoid cells and T cells by aryl hydrocarbon receptor. Front. Immunol..

[213] Saha K., Subramenium Ganapathy A., Wang A., Arumugam P., Michael Morris N., Harris L., Yochum G., Koltun W., Perdew G.H., Nighot M. (2023). Alpha-tocopherylquinone-mediated activation of the Aryl Hydrocarbon Receptor regulates the production of inflammation-inducing cytokines and ameliorates intestinal inflammation. Mucosal Immunol..

[214] Ivanov I.I., McKenzie B.S., Zhou L., Tadokoro C.E., Lepelley A., Lafaille J.J., Cua D.J., Littman D.R. (2006). The orphan nuclear receptor RORgammat directs the differentiation program of proinflammatory IL-17+ T helper cells. Cell.

[215] de Lima K.A., Donate P.B., Talbot J., Davoli-Ferreira M., Peres R.S., Cunha T.M., Alves-Filho J.C., Cunha F.Q. (2018). TGFbeta1 signaling sustains aryl hydrocarbon receptor (AHR) expression and restrains the pathogenic potential of T(H)17 cells by an AHR-independent mechanism. Cell Death Dis..

[216] Nakano N., Sakata N., Katsu Y., Nochise D., Sato E., Takahashi Y., Yamaguchi S., Haga Y., Ikeno S., Motizuki M. (2020). Dissociation of the AhR/ARNT complex by TGF-beta/Smad signaling represses CYP1A1 gene expression and inhibits benze[a]pyrene-mediated cytotoxicity. J. Biol. Chem..

[217] Silginer M., Burghardt I., Gramatzki D., Bunse L., Leske H., Rushing E.J., Hao N., Platten M., Weller M., Roth P. (2016). The aryl hydrocarbon receptor links integrin signaling to the TGF-beta pathway. Oncogene.

[218] Kung T., Murphy K.A., White L.A. (2009). The aryl hydrocarbon receptor (AhR) pathway as a regulatory pathway for cell adhesion and matrix metabolism. Biochem. Pharmacol..

[219] Starsichova A., Hruba E., Slabakova E., Pernicova Z., Prochazkova J., Pencikova K., Seda V., Kabatkova M., Vondracek J., Kozubik A. (2012). TGF-beta1 signaling plays a dominant role in the crosstalk between TGF-beta1 and the aryl hydrocarbon receptor ligand in prostate epithelial cells. Cell Signal.

[220] Worthington J.J., Fenton T.M., Czajkowska B.I., Klementowicz J.E., Travis M.A. (2012). Regulation of TGFbeta in the immune system: An emerging role for integrins and dendritic cells. Immunobiology.

[221] Deng Z., Fan T., Xiao C., Tian H., Zheng Y., Li C., He J. (2024). TGF-beta signaling in health, disease, and therapeutics. Signal Transduct. Target. Ther..

[222] Chi Y., Jiang H., Yin Y., Zhou X., Shao Y., Li Y., Rao J. (2025). Macrophage Signaling Pathways in Health and Disease: From Bench to Bedside Applications. MedComm.

[223] Kurose H. (2021). Cardiac Fibrosis and Fibroblasts. Cells.

[224] Zimmerman E., Sturrock A., Reilly C.A., Burrell-Gerbers K.L., Warren K., Mir-Kasimov M., Zhang M.A., Pierce M.S., Helms M.N., Paine R. (2024). Aryl Hydrocarbon Receptor Activation in Pulmonary Alveolar Epithelial Cells Limits Inflammation and Preserves Lung Epithelial Cell Integrity. J. Immunol..

[225] Saito A., Horie M., Nagase T. (2018). TGF-beta Signaling in Lung Health and Disease. Int. J. Mol. Sci..

[226] Sanjabi S., Zenewicz L.A., Kamanaka M., Flavell R.A. (2009). Anti-inflammatory and pro-inflammatory roles of TGF-beta, IL-10, and IL-22 in immunity and autoimmunity. Curr. Opin. Pharmacol..

[227] Yu H.X., Feng Z., Lin W., Yang K., Liu R.Q., Li J.Q., Liu X.Y., Pei M., Yang H.T. (2022). Ongoing Clinical Trials in Aging-Related Tissue Fibrosis and New Findings Related to AhR Pathways. Aging Dis..

[228] Curran C.S., Kopp J.B. (2022). Aryl Hydrocarbon Receptor Mechanisms Affecting Chronic Kidney Disease. Front. Pharmacol..

[229] Mezrich J.D., Fechner J.H., Zhang X., Johnson B.P., Burlingham W.J., Bradfield C.A. (2010). An interaction between kynurenine and the aryl hydrocarbon receptor can generate regulatory T cells. J. Immunol..

[230] Xu L., Kitani A., Strober W. (2010). Molecular mechanisms regulating TGF-beta-induced Foxp3 expression. Mucosal Immunol..

[231] Chen W., Konkel J.E. (2010). TGF-beta and ‘adaptive’ Foxp3(+) regulatory T cells. J. Mol. Cell. Biol..

[232] Polonio C.M., McHale K.A., Sherr D.H., Rubenstein D., Quintana F.J. (2025). The aryl hydrocarbon receptor: A rehabilitated target for therapeutic immune modulation. Nat. Rev. Drug Discov..

[233] Li H., Fan Y., Liu J., Dong S., Wen B., Zhang Y., Wang X., Duan X., Hu Y., Yan Z. (2025). Aryl Hydrocarbon Receptor in Health and Disease. MedComm.

[234] Hanieh H. (2014). Toward understanding the role of aryl hydrocarbon receptor in the immune system: Current progress and future trends. Biomed. Res. Int..

[235] Hagan A.S., Williams S., Mathison C.J.N., Yan S., Nguyen B., Federe G.C., Kuzu G., Siefert J.C., Hampton J., Chichkov V. (2025). Triggering AHR resolves TGF-beta1 induced fibroblast activation and promotes AT1 cell regeneration in alveolar organoids. Commun. Biol..

[236] Minacori M., Fiorini S., Perugini M., Iannetta A., Meschiari G., Chichiarelli S., Altieri F., Natali P.G., Eufemi M. (2025). AhR and STAT3: A Dangerous Duo in Chemical Carcinogenesis. Int. J. Mol. Sci..

[237] Quintana F.J. (2014). LeA(H)Rning self-control. Cell Res..

[238] Wang Y., Yang B.H., Li H., Cao S., Ren X.B., Yu J.P. (2013). IDO(+) DCs and signalling pathways. Curr. Cancer Drug Targets.

[239] Yu J., Wang Y., Yan F., Zhang P., Li H., Zhao H., Yan C., Yan F., Ren X. (2014). Noncanonical NF-kappaB activation mediates STAT3-stimulated IDO upregulation in myeloid-derived suppressor cells in breast cancer. J. Immunol..

[240] Riaz F., Wei P., Pan F. (2022). Fine-tuning of regulatory T cells is indispensable for the metabolic steatosis-related hepatocellular carcinoma: A review. Front. Cell Dev. Biol..

[241] Walczak K., Wnorowski A., Turski W.A., Plech T. (2020). Kynurenic acid and cancer: Facts and controversies. Cell. Mol. Life Sci..

[242] Chavda V.P., Bezbaruah R., Ahmed N., Alom S., Bhattacharjee B., Nalla L.V., Rynjah D., Gadanec L.K., Apostolopoulos V. (2025). Proinflammatory Cytokines in Chronic Respiratory Diseases and Their Management. Cells.

[243] Liu F., Dai W., Li C., Lu X., Chen Y., Weng D., Chen J. (2016). Role of IL-10-producing regulatory B cells in modulating T-helper cell immune responses during silica-induced lung inflammation and fibrosis. Sci. Rep..

[244] Komai T., Inoue M., Okamura T., Morita K., Iwasaki Y., Sumitomo S., Shoda H., Yamamoto K., Fujio K. (2018). Transforming Growth Factor-beta and Interleukin-10 Synergistically Regulate Humoral Immunity via Modulating Metabolic Signals. Front. Immunol..

[245] Wong P.S., Vogel C.F., Kokosinski K., Matsumura F. (2010). Arylhydrocarbon receptor activation in NCI-H441 cells and C57BL/6 mice: Possible mechanisms for lung dysfunction. Am. J. Respir. Cell Mol. Biol..

[246] Shivanna B., Jiang W., Wang L., Couroucli X.I., Moorthy B. (2011). Omeprazole attenuates hyperoxic lung injury in mice via aryl hydrocarbon receptor activation and is associated with increased expression of cytochrome P4501A enzymes. J. Pharmacol. Exp. Ther..

[247] Moon D.O., Kim M.O., Lee H.J., Choi Y.H., Park Y.M., Heo M.S., Kim G.Y. (2008). Curcumin attenuates ovalbumin-induced airway inflammation by regulating nitric oxide. Biochem. Biophys. Res. Commun..

[248] Baglole C.J., Maggirwar S.B., Gasiewicz T.A., Thatcher T.H., Phipps R.P., Sime P.J. (2008). The aryl hydrocarbon receptor attenuates tobacco smoke-induced cyclooxygenase-2 and prostaglandin production in lung fibroblasts through regulation of the NF-kappaB family member RelB. J. Biol. Chem..

[249] Martey C.A., Baglole C.J., Gasiewicz T.A., Sime P.J., Phipps R.P. (2005). The aryl hydrocarbon receptor is a regulator of cigarette smoke induction of the cyclooxygenase and prostaglandin pathways in human lung fibroblasts. Am. J. Physiol. Lung Cell. Mol. Physiol..

[250] Thatcher T.H., Maggirwar S.B., Baglole C.J., Lakatos H.F., Gasiewicz T.A., Phipps R.P., Sime P.J. (2007). Aryl hydrocarbon receptor-deficient mice develop heightened inflammatory responses to cigarette smoke and endotoxin associated with rapid loss of the nuclear factor-kappaB component RelB. Am. J. Pathol..

[251] Guerrina N., Traboulsi H., Eidelman D.H., Baglole C.J. (2021). The Aryl Hydrocarbon Receptor Suppresses Chronic Smoke-Induced Pulmonary Inflammation. Front. Toxicol..

[252] Rico de Souza A., Traboulsi H., Wang X., Fritz J.H., Eidelman D.H., Baglole C.J. (2021). The Aryl Hydrocarbon Receptor Attenuates Acute Cigarette Smoke-Induced Airway Neutrophilia Independent of the Dioxin Response Element. Front. Immunol..

[253] de Souza A.R., Zago M., Eidelman D.H., Hamid Q., Baglole C.J. (2014). Aryl hydrocarbon receptor (AhR) attenuation of subchronic cigarette smoke-induced pulmonary neutrophilia is associated with retention of nuclear RelB and suppression of intercellular adhesion molecule-1 (ICAM-1). Toxicol. Sci..

[254] Rico de Souza A., Zago M., Pollock S.J., Sime P.J., Phipps R.P., Baglole C.J. (2011). Genetic ablation of the aryl hydrocarbon receptor causes cigarette smoke-induced mitochondrial dysfunction and apoptosis. J. Biol. Chem..

[255] Cervilha D.A.B., Ito J.T., Lourenco J.D., Olivo C.R., Saraiva-Romanholo B.M., Volpini R.A., Oliveira-Junior M.C., Mauad T., Martins M.A., Tiberio I. (2019). The Th17/Treg Cytokine Imbalance in Chronic Obstructive Pulmonary Disease Exacerbation in an Animal Model of Cigarette Smoke Exposure and Lipopolysaccharide Challenge Association. Sci. Rep..

[256] Ovrevik J., Lag M., Lecureur V., Gilot D., Lagadic-Gossmann D., Refsnes M., Schwarze P.E., Skuland T., Becher R., Holme J.A. (2014). AhR and Arnt differentially regulate NF-kappaB signaling and chemokine responses in human bronchial epithelial cells. Cell Commun. Signal.

[257] Alessandrini F., de Jong R., Wimmer M., Maier A.M., Fernandez I., Hils M., Buters J.T., Biedermann T., Zissler U.M., Hoffmann C. (2022). Lung Epithelial CYP1 Activity Regulates Aryl Hydrocarbon Receptor Dependent Allergic Airway Inflammation. Front. Immunol..

[258] Xu T., Zhou Y., Qiu L., Do D.C., Zhao Y., Cui Z., Wang H., Liu X., Saradna A., Cao X. (2015). Aryl Hydrocarbon Receptor Protects Lungs from Cockroach Allergen-Induced Inflammation by Modulating Mesenchymal Stem Cells. J. Immunol..

[259] Cui Z., Feng Y., Li D., Li T., Gao P., Xu T. (2020). Activation of aryl hydrocarbon receptor (AhR) in mesenchymal stem cells modulates macrophage polarization in asthma. J. Immunotoxicol..

[260] Luebke R.W., Copeland C.B., Daniels M., Lambert A.L., Gilmour M.I. (2001). Suppression of allergic immune responses to house dust mite (HDM) in rats exposed to 2,3,7,8-TCDD. Toxicol. Sci..

[261] Steering Committee A., Review Panel M. (2020). A WAO—ARIA—GA(2)LEN consensus document on molecular-based allergy diagnosis (PAMD@): Update 2020. World Allergy Organ J..

[262] Matricardi P.M., Kleine-Tebbe J., Hoffmann H.J., Valenta R., Hilger C., Hofmaier S., Aalberse R.C., Agache I., Asero R., Ballmer-Weber B. (2016). EAACI Molecular Allergology User’s Guide. Pediatr. Allergy Immunol..

[263] von Garnier C., Wikstrom M.E., Zosky G., Turner D.J., Sly P.D., Smith M., Thomas J.A., Judd S.R., Strickland D.H., Holt P.G. (2007). Allergic airways disease develops after an increase in allergen capture and processing in the airway mucosa. J. Immunol..

[264] Bacchetta R., Gambineri E., Roncarolo M.G. (2007). Role of regulatory T cells and FOXP3 in human diseases. J. Allergy Clin. Immunol..

[265] Bacher P., Heinrich F., Stervbo U., Nienen M., Vahldieck M., Iwert C., Vogt K., Kollet J., Babel N., Sawitzki B. (2016). Regulatory T Cell Specificity Directs Tolerance versus Allergy against Aeroantigens in Humans. Cell.

[266] Larche M. (2007). Regulatory T cells in allergy and asthma. Chest.

[267] Matucci A., Vultaggio A., Maggi E., Kasujee I. (2018). Is IgE or eosinophils the key player in allergic asthma pathogenesis? Are we asking the right question?. Respir. Res..

[268] Xia M., Viera-Hutchins L., Garcia-Lloret M., Noval Rivas M., Wise P., McGhee S.A., Chatila Z.K., Daher N., Sioutas C., Chatila T.A. (2015). Vehicular exhaust particles promote allergic airway inflammation through an aryl hydrocarbon receptor-notch signaling cascade. J. Allergy Clin. Immunol..

[269] Wong T.H., Lee C.L., Su H.H., Lee C.L., Wu C.C., Wang C.C., Sheu C.C., Lai R.S., Leung S.Y., Lin C.C. (2018). A prominent air pollutant, Indeno [1,2,3-cd]pyrene, enhances allergic lung inflammation via aryl hydrocarbon receptor. Sci. Rep..

[270] Xia M., Harb H., Saffari A., Sioutas C., Chatila T.A. (2018). A Jagged 1-Notch 4 molecular switch mediates airway inflammation induced by ultrafine particles. J. Allergy Clin. Immunol..

[271] Vogel C.F.A., Van Winkle L.S., Esser C., Haarmann-Stemmann T. (2020). The aryl hydrocarbon receptor as a target of environmental stressors—Implications for pollution mediated stress and inflammatory responses. Redox Biol..

[272] Vestbo J., Hurd S.S., Agusti A.G., Jones P.W., Vogelmeier C., Anzueto A., Barnes P.J., Fabbri L.M., Martinez F.J., Nishimura M. (2013). Global strategy for the diagnosis, management, and prevention of chronic obstructive pulmonary disease: GOLD executive summary. Am. J. Respir. Crit. Care Med..

[273] Takenaka H., Zhang K., Diaz-Sanchez D., Tsien A., Saxon A. (1995). Enhanced human IgE production results from exposure to the aromatic hydrocarbons from diesel exhaust: Direct effects on B-cell IgE production. J. Allergy Clin. Immunol..

[274] van Voorhis M., Knopp S., Julliard W., Fechner J.H., Zhang X., Schauer J.J., Mezrich J.D. (2013). Exposure to atmospheric particulate matter enhances Th17 polarization through the aryl hydrocarbon receptor. PLoS ONE.

[275] Wang E., Liu X., Tu W., Do D.C., Yu H., Yang L., Zhou Y., Xu D., Huang S.K., Yang P. (2019). Benzo(a)pyrene facilitates dermatophagoides group 1 (Der f 1)-induced epithelial cytokine release through aryl hydrocarbon receptor in asthma. Allergy.

[276] Castaneda A.R., Vogel C.F.A., Bein K.J., Hughes H.K., Smiley-Jewell S., Pinkerton K.E. (2018). Ambient particulate matter enhances the pulmonary allergic immune response to house dust mite in a BALB/c mouse model by augmenting Th2- and Th17-immune responses. Physiol. Rep..

[277] Hwang Y.J., Yun M.O., Jeong K.T., Park J.H. (2014). Uremic toxin indoxyl 3-sulfate regulates the differentiation of Th2 but not of Th1 cells to lessen allergic asthma. Toxicol. Lett..

[278] Li X.M., Peng J., Gu W., Guo X.J. (2016). TCDD-Induced Activation of Aryl Hydrocarbon Receptor Inhibits Th17 Polarization and Regulates Non-Eosinophilic Airway Inflammation in Asthma. PLoS ONE.

[279] Sun L., Fu J., Lin S.H., Sun J.L., Xia L., Lin C.H., Liu L., Zhang C., Yang L., Xue P. (2020). Particulate matter of 2.5 mum or less in diameter disturbs the balance of T(H)17/regulatory T cells by targeting glutamate oxaloacetate transaminase 1 and hypoxia-inducible factor 1alpha in an asthma model. J. Allergy Clin. Immunol..

[280] Hammerschmidt-Kamper C., Biljes D., Merches K., Steiner I., Daldrup T., Bol-Schoenmakers M., Pieters R.H.H., Esser C. (2017). Indole-3-carbinol, a plant nutrient and AhR-Ligand precursor, supports oral tolerance against OVA and improves peanut allergy symptoms in mice. PLoS ONE.

[281] Sibilano R., Frossi B., Calvaruso M., Danelli L., Betto E., Dall’Agnese A., Tripodo C., Colombo M.P., Pucillo C.E., Gri G. (2012). The aryl hydrocarbon receptor modulates acute and late mast cell responses. J. Immunol..

[282] Schulz V.J., Smit J.J., Huijgen V., Bol-Schoenmakers M., van Roest M., Kruijssen L.J., Fiechter D., Hassing I., Bleumink R., Safe S. (2012). Non-dioxin-like AhR ligands in a mouse peanut allergy model. Toxicol. Sci..

[283] Janssen-Weets B., Kerff F., Swiontek K., Kler S., Czolk R., Revets D., Kuehn A., Bindslev-Jensen C., Ollert M., Hilger C. (2022). Mammalian derived lipocalin and secretoglobin respiratory allergens strongly bind ligands with potentially immune modulating properties. Front. Allergy.

[284] Roth-Walter F., Afify S.M., Pacios L.F., Blokhuis B.R., Redegeld F., Regner A., Petje L.M., Fiocchi A., Untersmayr E., Dvorak Z. (2021). Cow’s milk protein beta-lactoglobulin confers resilience against allergy by targeting complexed iron into immune cells. J. Allergy Clin. Immunol..

[285] Jeong K.T., Hwang S.J., Oh G.S., Park J.H. (2012). FICZ, a tryptophan photoproduct, suppresses pulmonary eosinophilia and Th2-type cytokine production in a mouse model of ovalbumin-induced allergic asthma. Int. Immunopharmacol..

[286] Wang E., Tu W., Do D.C., Xiao X., Bhatti S.B., Yang L., Sun X., Xu D., Yang P., Huang S.K. (2021). Benzo(a)pyrene Enhanced Dermatophagoides Group 1 (Der f 1)-Induced TGFbeta1 Signaling Activation Through the Aryl Hydrocarbon Receptor-RhoA Axis in Asthma. Front. Immunol..

[287] Hu X., Shen Y., Zhao Y., Wang J., Zhang X., Tu W., Kaufman W., Feng J., Gao P. (2021). Epithelial Aryl Hydrocarbon Receptor Protects From Mucus Production by Inhibiting ROS-Triggered NLRP3 Inflammasome in Asthma. Front. Immunol..

[288] Wang J., Zhao Y., Zhang X., Tu W., Wan R., Shen Y., Zhang Y., Trivedi R., Gao P. (2022). Type II alveolar epithelial cell aryl hydrocarbon receptor protects against allergic airway inflammation through controlling cell autophagy. Front. Immunol..

[289] Xu X., Zhang X., Yuan Y., Zhao Y., Fares H.M., Yang M., Wen Q., Taha R., Sun L. (2021). Species-Specific Differences in Aryl Hydrocarbon Receptor Responses: How and Why?. Int. J. Mol. Sci..

[290] Flaveny C.A., Murray I.A., Chiaro C.R., Perdew G.H. (2009). Ligand selectivity and gene regulation by the human aryl hydrocarbon receptor in transgenic mice. Mol. Pharmacol..

[291] Vogel C.F.A., Lazennec G., Kado S.Y., Dahlem C., He Y., Castaneda A., Ishihara Y., Vogeley C., Rossi A., Haarmann-Stemmann T. (2021). Targeting the Aryl Hydrocarbon Receptor Signaling Pathway in Breast Cancer Development. Front. Immunol..

[292] Flaveny C.A., Murray I.A., Perdew G.H. (2010). Differential gene regulation by the human and mouse aryl hydrocarbon receptor. Toxicol. Sci..

[293] Garcia G.R., Shankar P., Dunham C.L., Garcia A., La Du J.K., Truong L., Tilton S.C., Tanguay R.L. (2018). Signaling Events Downstream of AHR Activation That Contribute to Toxic Responses: The Functional Role of an AHR-Dependent Long Noncoding RNA (slincR) Using the Zebrafish Model. Environ. Health Perspect..

[294] Flaveny C.A., Perdew G.H. (2009). Transgenic Humanized AHR Mouse Reveals Differences between Human and Mouse AHR Ligand Selectivity. Mol. Cell. Pharmacol..

[295] Pathak U., Gupta N.C., Suri J.C. (2020). Risk of COPD due to indoor air pollution from biomass cooking fuel: A systematic review and meta-analysis. Int. J. Environ. Health Res..

[296] Brunekreef B., Forsberg B. (2005). Epidemiological evidence of effects of coarse airborne particles on health. Eur. Respir. J..

[297] Consonni D., Pesatori A.C., Zocchetti C., Sindaco R., D’Oro L.C., Rubagotti M., Bertazzi P.A. (2008). Mortality in a population exposed to dioxin after the Seveso, Italy, accident in 1976: 25 years of follow-up. Am. J. Epidemiol..

[298] Riedl M.A., Nel A.E. (2008). Importance of oxidative stress in the pathogenesis and treatment of asthma. Curr. Opin. Allergy Clin. Immunol..

[299] Barnes P.J. (1990). Reactive oxygen species and airway inflammation. Free Radic. Biol. Med..

[300] De Grove K.C., Provoost S., Brusselle G.G., Joos G.F., Maes T. (2018). Insights in particulate matter-induced allergic airway inflammation: Focus on the epithelium. Clin. Exp. Allergy.

[301] Holme J.A., Vondracek J., Machala M., Lagadic-Gossmann D., Vogel C.F.A., Le Ferrec E., Sparfel L., Ovrevik J. (2023). Lung cancer associated with combustion particles and fine particulate matter (PM(2.5))—The roles of polycyclic aromatic hydrocarbons (PAHs) and the aryl hydrocarbon receptor (AhR). Biochem. Pharmacol..

[302] Li L., Yang D.C., Chen C.H. (2021). Metabolic reprogramming: A driver of cigarette smoke-induced inflammatory lung diseases. Free Radic. Biol. Med..

[303] Tan W.C., Sin D.D., Bourbeau J., Hernandez P., Chapman K.R., Cowie R., FitzGerald J.M., Marciniuk D.D., Maltais F., Buist A.S. (2015). Characteristics of COPD in never-smokers and ever-smokers in the general population: Results from the CanCOLD study. Thorax.

[304] Tamimi A., Serdarevic D., Hanania N.A. (2012). The effects of cigarette smoke on airway inflammation in asthma and COPD: Therapeutic implications. Respir. Med..

[305] Hogg J.C., Chu F., Utokaparch S., Woods R., Elliott W.M., Buzatu L., Cherniack R.M., Rogers R.M., Sciurba F.C., Coxson H.O. (2004). The nature of small-airway obstruction in chronic obstructive pulmonary disease. N. Engl. J. Med..

[306] Lane N., Robins R.A., Corne J., Fairclough L. (2010). Regulation in chronic obstructive pulmonary disease: The role of regulatory T-cells and Th17 cells. Clin. Sci..

[307] MacNee W. (2005). Pathogenesis of chronic obstructive pulmonary disease. Proc. Am. Thorac. Soc..

[308] Sales D.S., Ito J.T., Zanchetta I.A., Annoni R., Aun M.V., Ferraz L.F.S., Cervilha D.A.B., Negri E., Mauad T., Martins M.A. (2017). Regulatory T-Cell Distribution within Lung Compartments in COPD. COPD J. Chronic Obstr. Pulm. Dis..

[309] Silva L.E.F., Lourenco J.D., Silva K.R., Santana F.P.R., Kohler J.B., Moreira A.R., Velosa A.P.P., Prado C.M., Vieira R.P., Aun M.V. (2020). Th17/Treg imbalance in COPD development: Suppressors of cytokine signaling and signal transducers and activators of transcription proteins. Sci. Rep..

[310] Lourenco J.D., Teodoro W.R., Barbeiro D.F., Velosa A.P.P., Silva L.E.F., Kohler J.B., Moreira A.R., Aun M.V., da Silva I.C., Fernandes F.L.A. (2021). Th17/Treg-Related Intracellular Signaling in Patients with Chronic Obstructive Pulmonary Disease: Comparison between Local and Systemic Responses. Cells.

[311] Mortaz E., Folkerts G., Redegeld F. (2011). Mast cells and COPD. Pulm. Pharmacol. Ther..

[312] Chiba T., Uchi H., Tsuji G., Gondo H., Moroi Y., Furue M. (2011). Arylhydrocarbon receptor (AhR) activation in airway epithelial cells induces MUC5AC via reactive oxygen species (ROS) production. Pulm. Pharmacol. Ther..

[313] Martinez J.M., Afshari C.A., Bushel P.R., Masuda A., Takahashi T., Walker N.J. (2002). Differential toxicogenomic responses to 2,3,7,8-tetrachlorodibenzo-p-dioxin in malignant and nonmalignant human airway epithelial cells. Toxicol. Sci..

[314] Sheridan J.A., Zago M., Nair P., Li P.Z., Bourbeau J., Tan W.C., Hamid Q., Eidelman D.H., Benedetti A.L., Baglole C.J. (2015). Decreased expression of the NF-kappaB family member RelB in lung fibroblasts from Smokers with and without COPD potentiates cigarette smoke-induced COX-2 expression. Respir. Res..

[315] Tsai M.J., Hsu Y.L., Wang T.N., Wu L.Y., Lien C.T., Hung C.H., Kuo P.L., Huang M.S. (2014). Aryl hydrocarbon receptor (AhR) agonists increase airway epithelial matrix metalloproteinase activity. J. Mol. Med..

[316] Guerrina N., Traboulsi H., Eidelman D.H., Baglole C.J. (2018). The Aryl Hydrocarbon Receptor and the Maintenance of Lung Health. Int. J. Mol. Sci..

[317] Thorley A.J., Tetley T.D. (2007). Pulmonary epithelium, cigarette smoke, and chronic obstructive pulmonary disease. Int. J. Chron. Obs. Pulmon Dis..

[318] Casara A., Turato G., Marin-Oto M., Semenzato U., Biondini D., Tine M., Bernardinello N., Cocconcelli E., Cubero P., Balestro E. (2022). Chronic Bronchitis Affects Outcomes in Smokers without Chronic Obstructive Pulmonary Disease (COPD). J. Clin. Med..

[319] Laniado-Laborin R. (2009). Smoking and chronic obstructive pulmonary disease (COPD). Parallel epidemics of the 21^st^ century. Int. J. Environ. Res. Public Health.

[320] Onozuka D., Nakamura Y., Tsuji G., Furue M. (2020). Mortality in Yusho patients exposed to polychlorinated biphenyls and polychlorinated dibenzofurans: A 50-year retrospective cohort study. Environ. Health.

[321] Guerrina N., Traboulsi H., Rico de Souza A., Bosse Y., Thatcher T.H., Robichaud A., Ding J., Li P.Z., Simon L., Pareek S. (2021). Aryl hydrocarbon receptor deficiency causes the development of chronic obstructive pulmonary disease through the integration of multiple pathogenic mechanisms. FASEB J..

[322] Sadr M., Asgari A., Assadiasl S., Mojtahedi H., Abdollahi A., Nicknam M.H., Edalatifard M., Soleimanifar N. (2025). Decreased Aryl Hydrocarbon Receptor Gene Expression and Polymorphic Variants Association with Increase COPD Risk. Int. J. Chron. Obs. Pulmon. Dis..

[323] Rogers S., de Souza A.R., Zago M., Iu M., Guerrina N., Gomez A., Matthews J., Baglole C.J. (2017). Aryl hydrocarbon receptor (AhR)-dependent regulation of pulmonary miRNA by chronic cigarette smoke exposure. Sci. Rep..

[324] Shivanna B., Chu C., Moorthy B. (2022). The Aryl Hydrocarbon Receptor (AHR): A Novel Therapeutic Target for Pulmonary Diseases?. Int. J. Mol. Sci..

[325] Lo C.Y., Huang H.Y., He J.R., Huang T.T., Heh C.C., Sheng T.F., Chung K.F., Kuo H.P., Wang C.H. (2018). Increased matrix metalloproteinase-9 to tissue inhibitor of metalloproteinase-1 ratio in smokers with airway hyperresponsiveness and accelerated lung function decline. Int. J. Chron. Obs. Pulmon Dis..

[326] Chen G., Ge D., Zhu B., Shi H., Ma Q. (2020). Upregulation of matrix metalloproteinase 9 (MMP9)/tissue inhibitor of metalloproteinase 1 (TIMP1) and MMP2/TIMP2 ratios may be involved in lipopolysaccharide-induced acute lung injury. J. Int. Med. Res..

[327] Aoshiba K., Yokohori N., Nagai A. (2003). Alveolar wall apoptosis causes lung destruction and emphysematous changes. Am. J. Respir. Cell Mol. Biol..

[328] Marin-Hinojosa C., Eraso C.C., Sanchez-Lopez V., Hernandez L.C., Otero-Candelera R., Lopez-Campos J.L. (2021). Nutriepigenomics and chronic obstructive pulmonary disease: Potential role of dietary and epigenetics factors in disease development and management. Am. J. Clin. Nutr..

[329] Chen Q., Jia Z., Qu C. (2022). Inhibition of KLF6 reduces the inflammation and apoptosis of type II alveolar epithelial cells in acute lung injury. Allergol. Immunopathol..

[330] Traboulsi H., Heimbach N.S., Wilson E.T., Eidelman D.H., Baglole C.J. (2026). The aryl hydrocarbon receptor promotes the resolution of pulmonary neutrophilia via regulation of macrophage efferocytosis. J. Immunol..

[331] Zhu J., Luo L., Tian L., Yin S., Ma X., Cheng S., Tang W., Yu J., Ma W., Zhou X. (2018). Aryl Hydrocarbon Receptor Promotes IL-10 Expression in Inflammatory Macrophages Through Src-STAT3 Signaling Pathway. Front. Immunol..

[332] Zago M., Sheridan J.A., Nair P., Rico de Souza A., Gallouzi I.E., Rousseau S., Di Marco S., Hamid Q., Eidelman D.H., Baglole C.J. (2013). Aryl hydrocarbon receptor-dependent retention of nuclear HuR suppresses cigarette smoke-induced cyclooxygenase-2 expression independent of DNA-binding. PLoS ONE.

[333] Martel-Pelletier J., Lajeunesse D., Reboul P., Pelletier J.P. (2003). Therapeutic role of dual inhibitors of 5-LOX and COX, selective and non-selective non-steroidal anti-inflammatory drugs. Ann. Rheum. Dis..

[334] Dietrich C. (2016). Antioxidant Functions of the Aryl Hydrocarbon Receptor. Stem Cells Int..

[335] Grishanova A.Y., Perepechaeva M.L. (2022). Aryl Hydrocarbon Receptor in Oxidative Stress as a Double Agent and Its Biological and Therapeutic Significance. Int. J. Mol. Sci..

[336] Sarill M., Zago M., Sheridan J.A., Nair P., Matthews J., Gomez A., Roussel L., Rousseau S., Hamid Q., Eidelman D.H. (2015). The aryl hydrocarbon receptor suppresses cigarette-smoke-induced oxidative stress in association with dioxin response element (DRE)-independent regulation of sulfiredoxin 1. Free Radic. Biol. Med..

[337] Thome T., Miguez K., Willms A.J., Burke S.K., Chandran V., de Souza A.R., Fitzgerald L.F., Baglole C., Anagnostou M.E., Bourbeau J. (2022). Chronic aryl hydrocarbon receptor activity phenocopies smoking-induced skeletal muscle impairment. J. Cachexia Sarcopenia Muscle.

[338] Chiba T., Chihara J., Furue M. (2012). Role of the Arylhydrocarbon Receptor (AhR) in the Pathology of Asthma and COPD. J. Allergy.

[339] Duarte-Hospital C., Tete A., Brial F., Benoit L., Koual M., Tomkiewicz C., Kim M.J., Blanc E.B., Coumoul X., Bortoli S. (2021). Mitochondrial Dysfunction as a Hallmark of Environmental Injury. Cells.

[340] Cheng T.Y., Luo C.S., Feng P.H., Chen K.Y., Chang C.C., Van Hiep N., Chen Y.H., Yeh Y.K., Wu S.M. (2025). Polycyclic aromatic hydrocarbon-aryl hydrocarbon receptor signaling regulates chronic inflammation in lung-gut axis. Toxicol. Appl. Pharmacol..

[341] Weng C.M., Wang C.H., Lee M.J., He J.R., Huang H.Y., Chao M.W., Chung K.F., Kuo H.P. (2018). Aryl hydrocarbon receptor activation by diesel exhaust particles mediates epithelium-derived cytokines expression in severe allergic asthma. Allergy.

[342] Holgate S.T. (2012). Innate and adaptive immune responses in asthma. Nat. Med..

[343] Lopes F., Tiberio I., Leme A., Fairclough L. (2022). Editorial: The importance of Th17/Treg imbalance in asthma and COPD development and progression. Front. Immunol..

[344] Guan Y., Ma Y., Tang Y., Liu X., Zhao Y., An L. (2021). MiRNA-221-5p suppressed the Th17/Treg ratio in asthma via RORgammat/Foxp3 by targeting SOCS1. Allergy Asthma Clin. Immunol..

[345] Finkelman F.D., Hogan S.P., Hershey G.K., Rothenberg M.E., Wills-Karp M. (2010). Importance of cytokines in murine allergic airway disease and human asthma. J. Immunol..

[346] McGrath K.W., Icitovic N., Boushey H.A., Lazarus S.C., Sutherland E.R., Chinchilli V.M., Fahy J.V., Asthma Clinical Research Network of the National Heart, Lung, and Blood Institute (2012). A large subgroup of mild-to-moderate asthma is persistently noneosinophilic. Am. J. Respir. Crit. Care Med..

[347] Aujla S.J., Alcorn J.F. (2011). T(H)17 cells in asthma and inflammation. Biochim. Biophys. Acta.

[348] Trejo Bittar H.E., Yousem S.A., Wenzel S.E. (2015). Pathobiology of severe asthma. Annu. Rev. Pathol..

[349] Matsumoto K., Inoue H., Fukuyama S., Kan O.K., Eguchi-Tsuda M., Matsumoto T., Moriwaki A., Nakano T., Nakanishi Y. (2009). Frequency of Foxp3^+^CD4^+^CD25^+^ T cells is associated with the phenotypes of allergic asthma. Respirology.

[350] Palomares O., Martin-Fontecha M., Lauener R., Traidl-Hoffmann C., Cavkaytar O., Akdis M., Akdis C.A. (2014). Regulatory T cells and immune regulation of allergic diseases: Roles of IL-10 and TGF-beta. Genes. Immun..

[351] Vroman H., van den Blink B., Kool M. (2015). Mode of dendritic cell activation: The decisive hand in Th2/Th17 cell differentiation. Implications in asthma severity?. Immunobiology.

[352] Ple C., Fan Y., Ait Yahia S., Vorng H., Everaere L., Chenivesse C., Balsamelli J., Azzaoui I., de Nadai P., Wallaert B. (2015). Polycyclic aromatic hydrocarbons reciprocally regulate IL-22 and IL-17 cytokines in peripheral blood mononuclear cells from both healthy and asthmatic subjects. PLoS ONE.

[353] Zhou Y., Mirza S., Xu T., Tripathi P., Plunkett B., Myers A., Gao P. (2014). Aryl hydrocarbon receptor (AhR) modulates cockroach allergen-induced immune responses through active TGFbeta1 release. Mediat. Inflamm..

[354] Reza M.I., Kumar A., Britt R.D., Sathish V. (2026). The Role of Aryl Hydrocarbon Receptor in Airway Remodeling: Mechanistic Insights Across Cellular Functions. Clin. Rev. Allergy Immunol..

[355] Vavilin V.A., Makarova S.I., Lyakhovich V.V., Gavalov S.M. (2002). Polymorphic Genes of Xenobiotic-Metabolizing Enzymes Associated with Predisposition to Bronchial Asthma in Hereditarily Burdened and Nonburdened Children. Russ. J. Genet..

[356] Samara K.D., Margaritopoulos G., Wells A.U., Siafakas N.M., Antoniou K.M. (2011). Smoking and pulmonary fibrosis: Novel insights. Pulm. Med..

[357] Wu S.M., Tsai J.J., Pan H.C., Arbiser J.L., Elia L., Sheu M.L. (2022). Aggravation of pulmonary fibrosis after knocking down the aryl hydrocarbon receptor in the insulin-like growth factor 1 receptor pathway. Br. J. Pharmacol..

[358] Gurczynski S.J., Moore B.B. (2018). IL-17 in the lung: The good, the bad, and the ugly. Am. J. Physiol. Lung Cell. Mol. Physiol..

[359] Takei H., Yasuoka H., Yoshimoto K., Takeuchi T. (2020). Aryl hydrocarbon receptor signals attenuate lung fibrosis in the bleomycin-induced mouse model for pulmonary fibrosis through increase of regulatory T cells. Arthritis Res. Ther..

[360] Antoniou K.M., Margaritopoulos G.A., Tomassetti S., Bonella F., Costabel U., Poletti V. (2014). Interstitial lung disease. Eur. Respir. Rev..

[361] Xu J., Li C., Zhao J., Mu R. (2025). Shared immunological pathways in rheumatoid arthritis-related interstitial lung disease. Front. Immunol..

[362] Al-Baldawi S., Zuniga Salazar G., Zuniga D., Balasubramanian S., Mehmood K.T. (2024). Interstitial Lung Disease in Rheumatoid Arthritis: A Review. Cureus.

[363] Yue T., Sun F., Yang C., Wang F., Luo J., Yang P., Xiong F., Zhang S., Yu Q., Wang C.Y. (2020). The AHR Signaling Attenuates Autoimmune Responses During the Development of Type 1 Diabetes. Front. Immunol..

[364] Cannon A.S., Nagarkatti P.S., Nagarkatti M. (2021). Targeting AhR as a Novel Therapeutic Modality against Inflammatory Diseases. Int. J. Mol. Sci..

[365] Wajda A., Paradowska-Gorycka A., Esser C. (2025). Systemic sclerosis and AHR: Shedding light on a hidden connections. Autoimmun. Rev..

[366] Takei H., Yasuoka H., Yoshimoto K., Yamaoka K., Takeuchi T. (2018). Aryl hydrocarbon receptor expression is associated with lung involvement in systemic sclerosis. Ann. Rheum. Dis..

[367] O’Driscoll C.A., Mezrich J.D. (2018). The Aryl Hydrocarbon Receptor as an Immune-Modulator of Atmospheric Particulate Matter-Mediated Autoimmunity. Front. Immunol..

[368] Kadura S., Raghu G. (2021). Rheumatoid arthritis-interstitial lung disease: Manifestations and current concepts in pathogenesis and management. Eur. Respir. Rev..

[369] Cheng L., Qian L., Xu Z.Z., Tan Y., Luo C.Y. (2019). 10.1016/j.jbspin.2018.03.001Aromatic hydrocarbon receptor provides a link between smoking and rheumatoid arthritis in peripheral blood mononuclear cells. Clin. Exp. Rheumatol..

[370] Sigaux J., Biton J., Andre E., Semerano L., Boissier M.C. (2019). Air pollution as a determinant of rheumatoid arthritis. Jt. Bone Spine.

[371] Liu Y., Zhao N., Xu Q., Deng F., Wang P., Dong L., Lu X., Xia L., Wang M., Chen Z. (2022). MBL Binding with AhR Controls Th17 Immunity in Silicosis-Associated Lung Inflammation and Fibrosis. J. Inflamm. Res..

[372] Beamer C.A., Seaver B.P., Shepherd D.M. (2012). Aryl hydrocarbon receptor (AhR) regulates silica-induced inflammation but not fibrosis. Toxicol. Sci..

[373] Song L., Weng D., Dai W., Tang W., Chen S., Li C., Chen Y., Liu F., Chen J. (2014). Th17 can regulate silica-induced lung inflammation through an IL-1beta-dependent mechanism. J. Cell Mol. Med..

[374] Handra C.M., Gurzu I.L., Chirila M., Ghita I. (2023). Silicosis: New Challenges from an Old Inflammatory and Fibrotic Disease. Front. Biosci.-Landmark.

[375] Song L., Weng D., Liu F., Chen Y., Li C., Dong L., Tang W., Chen J. (2012). Tregs promote the differentiation of Th17 cells in silica-induced lung fibrosis in mice. PLoS ONE.

[376] Lu Y., Liu F., Li C., Chen Y., Weng D., Chen J. (2017). IL-10-Producing B Cells Suppress Effector T Cells Activation and Promote Regulatory T Cells in Crystalline Silica-Induced Inflammatory Response In Vitro. Mediat. Inflamm..

[377] Haarmann-Stemmann T., Reichert D., Coumoul X., Lawrence B.P., Perdew G.H., Sherr D.H., Weighardt H., Rolfes K.M., Esser C. (2025). The Janus-facedness of the aryl hydrocarbon receptor pathway Report of the 6th International AHR Meeting: Research, Prevention, Therapy. Biochem. Pharmacol..

[378] Shinde R., McGaha T.L. (2018). The Aryl Hydrocarbon Receptor: Connecting Immunity to the Microenvironment. Trends Immunol..

[379] Griffith B.D., Frankel T.L. (2024). The Aryl Hydrocarbon Receptor: Impact on the Tumor Immune Microenvironment and Modulation as a Potential Therapy. Cancers.

[380] Dawe H.R., Di Meglio P. (2025). The Aryl Hydrocarbon Receptor (AHR): Peacekeeper of the Skin. Int. J. Mol. Sci..

[381] Gargaro M., Scalisi G., Manni G., Mondanelli G., Grohmann U., Fallarino F. (2021). The Landscape of AhR Regulators and Coregulators to Fine-Tune AhR Functions. Int. J. Mol. Sci..

[382] Stockinger B., Diaz O.E., Wincent E. (2024). The influence of AHR on immune and tissue biology. EMBO Mol. Med..

[383] Lawrence B.P., Vorderstrasse B.A. (2013). New insights into the aryl hydrocarbon receptor as a modulator of host responses to infection. Semin. Immunopathol..

[384] Szelest M., Walczak K., Plech T. (2021). A New Insight into the Potential Role of Tryptophan-Derived AhR Ligands in Skin Physiological and Pathological Processes. Int. J. Mol. Sci..

[385] Major J., Crotta S., Finsterbusch K., Chakravarty P., Shah K., Frederico B., D’Antuono R., Green M., Meader L., Suarez-Bonnet A. (2023). Endothelial AHR activity prevents lung barrier disruption in viral infection. Nature.

[386] Sulentic C.E.W., Kaplan B.L.F., Lawrence B.P. (2025). Using the Key Characteristics Framework to Unlock the Mysteries of Aryl Hydrocarbon Receptor-Mediated Effects on the Immune System. Annu. Rev. Immunol..

[387] Kang H., Chen Z., Wang B., Chen Z. (2025). The AhR/IL-22 axis in chronic gut inflammation: Unraveling mechanisms and therapeutic prospects. Front. Immunol..

[388] Zhu C., Xie Q., Zhao B. (2014). The role of AhR in autoimmune regulation and its potential as a therapeutic target against CD4 T cell mediated inflammatory disorder. Int. J. Mol. Sci..

[389] Kou Z., Dai W. (2021). Aryl hydrocarbon receptor: Its roles in physiology. Biochem. Pharmacol..

[390] Simones T., Shepherd D.M. (2011). Consequences of AhR activation in steady-state dendritic cells. Toxicol. Sci..

[391] Ran L., Li C., Wang P., Tang J., Qu Z., Hao Y., Zhang Y. (2026). Aryl hydrocarbon receptor: A potential target for natural products in the treatment of inflammatory bowel disease. Front. Immunol..

[392] Bock K.W. (2020). Aryl hydrocarbon receptor (AHR)-mediated inflammation and resolution: Non-genomic and genomic signaling. Biochem. Pharmacol..

[393] Pan Y., Deng Y., Yang H., Yu M. (2025). The aryl hydrocarbon receptor: A promising target for intestinal fibrosis therapy. Pharmacol. Res..

[394] Dolciami D., Ballarotto M., Gargaro M., Lopez-Cara L.C., Fallarino F., Macchiarulo A. (2020). Targeting Aryl hydrocarbon receptor for next-generation immunotherapies: Selective modulators (SAhRMs) versus rapidly metabolized ligands (RMAhRLs). Eur. J. Med. Chem..

[395] Safe S., Han H., Goldsby J., Mohankumar K., Chapkin R.S. (2018). Aryl Hydrocarbon Receptor (AhR) Ligands as Selective AhR Modulators: Genomic Studies. Curr. Opin. Toxicol..

[396] Hou S., Yue Q., Hou X., Wu Q. (2025). Targeting the Aryl Hydrocarbon Receptor: The Potential of Indole Compounds in the Treatment of Cystic Fibrosis. Int. J. Mol. Sci..

[397] Jeong E., Hong H., Lee Y.A., Kim K.S. (2024). Potential Rheumatoid Arthritis-Associated Interstitial Lung Disease Treatment and Computational Approach for Future Drug Development. Int. J. Mol. Sci..

[398] Wang Y., Wu G.R., Yue H., Zhou Q., Zhang L., He L., Gu W., Gao R., Dong L., Zhang H. (2026). Corrigendum to “Kynurenine acts as a signaling molecule to attenuate pulmonary fibrosis by enhancing the AHR-PTEN axis” [J. Adv. Res. 71 (2025) 521-532]. J. Adv. Res..

[399] Cavagna L., Monti S., Grosso V., Boffini N., Scorletti E., Crepaldi G., Caporali R. (2013). The multifaceted aspects of interstitial lung disease in rheumatoid arthritis. Biomed. Res. Int..

[400] Zhu X.D., Lei X.P., Dong W.B. (2017). Resveratrol as a potential therapeutic drug for respiratory system diseases. Drug Des. Dev. Ther..

[401] Ding S., Wang H., Wang M., Bai L., Yu P., Wu W. (2019). Resveratrol alleviates chronic “real-world” ambient particulate matter-induced lung inflammation and fibrosis by inhibiting NLRP3 inflammasome activation in mice. Ecotoxicol. Environ. Saf..

[402] Busbee P.B., Rouse M., Nagarkatti M., Nagarkatti P.S. (2013). Use of natural AhR ligands as potential therapeutic modalities against inflammatory disorders. Nutr. Rev..

[403] Lou Y., Huang Z., Wu H., Zhou Y. (2022). Tranilast attenuates lipopolysaccharide-induced lung injury via the CXCR4/JAK2/STAT3 signaling pathway. Mol. Med. Rep..

[404] Talebpour Amiri F., Asghari M., Hosseinimehr S.J. (2025). Omeprazole attenuates irradiation-induced lung injury through the suppression of apoptosis and oxidative stress in mice. Med. Oncol..

[405] Shanle E.K., Xu W. (2011). Endocrine disrupting chemicals targeting estrogen receptor signaling: Identification and mechanisms of action. Chem. Res. Toxicol..

[406] Haupt J., Keminer O., Neser C., Windshugel B., Wiltzsch V., Schmidt J.R., Pliushcheuskaya P., Kunze G., Scholz U., Muller C. (2025). Novel aryl hydrocarbon receptor agonists as potential anti-inflammatory therapeutics: Identification and validation through drug repurposing. Biochem. Pharmacol..

